# A Guide to Design Functional Molecular Liquids with Tailorable Properties using Pyrene-Fluorescence as a Probe

**DOI:** 10.1038/s41598-017-03584-1

**Published:** 2017-06-13

**Authors:** Fengniu Lu, Tomohisa Takaya, Koichi Iwata, Izuru Kawamura, Akinori Saeki, Masashi Ishii, Kazuhiko Nagura, Takashi Nakanishi

**Affiliations:** 1 Frontier Molecules Group, International Center for Materials Nanoarchitectonics (WPI-MANA), National Institute for Materials Science (NIMS), 1-1 Namiki, Tsukuba, 305-0044 Japan; 2 Department of Chemistry, Faculty of Science, Gakushuin University, 1-5-1 Mejiro, Toshima-ku, Tokyo, 171-8588 Japan; 3 Graduate School of Engineering, Yokohama National University, 79-5 Tokiwadai, Hodogaya-ku, Yokohama, 240-8501 Japan; 4 Department of Applied Chemistry, Graduate School of Engineering, Osaka University, 2-1 Yamadaoka, Suita, Osaka 565-0871 Japan; 5 Surface Physics and Characterization Group, Research Center for Advanced Measurement and Characterization, 1-2-1 Sengen, Tsukuba, 305-0047 Japan; 6 International Center for Young Scientists, NIMS, 1-2-1 Sengen, Tsukuba, 305-0047 Japan

## Abstract

Solvent-free, nonvolatile, room-temperature alkylated-π functional molecular liquids (FMLs) are rapidly emerging as a new generation of fluid matter. However, precision design to tune their physicochemical properties remains a serious challenge because the properties are governed by subtle π-π interactions among functional π-units, which are very hard to control and characterize. Herein, we address the issue by probing π-π interactions with highly sensitive pyrene-fluorescence. A series of alkylated pyrene FMLs were synthesized. The photophysical properties were artfully engineered with rational modulation of the number, length, and substituent motif of alkyl chains attached to the pyrene unit. The different emission from the excimer to uncommon intermediate to the monomer scaled the pyrene-pyrene interactions in a clear trend, from stronger to weaker to negligible. Synchronously, the physical nature of these FMLs was regulated from inhomogeneous to isotropic. The inhomogeneity, unexplored before, was thoroughly investigated by ultrafast time-resolved spectroscopy techniques. The result provides a clearer image of liquid matter. Our methodology demonstrates a potential to unambiguously determine local molecular organizations of amorphous materials, which cannot be achieved by conventional structural analysis. Therefore this study provides a guide to design alkylated-π FMLs with tailorable physicochemical properties.

## Introduction

Soft matter, *e*.*g*., polymers, colloids, foams, emulsions, gels, liquid crystals, biomembranes, and complex fluids, is essential for supporting life^1–6^. Among them, liquids, including water, fuels, lubricants, solvents, and ionic liquids, are most widely used in industry, daily life, and scientific research. The vast application potentials of liquids are guaranteed by their fluidity, versatility, and compatibility with other materials, which is currently attracting wide attention. Recently, solvent-free, nonvolatile, room-temperature functional molecular liquids (FMLs)^7^ have emerged as a new generation of liquid materials. With an excellent processability, free deformability, and high thermal stability, FMLs have gained extensive interest in wide areas, including luminescence^8–14^, photoconduction^15–18^, molecular recognition^19, 20^, energy storage^21, 22^, and biological systems^23, 24^. Aiming for applications in flexible and bendable optoelectronics, we^8–10, 15, 16, 25, 26^ and others^11–13, 21, 27–32^ have developed the alkyl-π engineering strategy^33^ to prepare various alkylated-π FMLs by attaching bulky and flexible alkyl chains to optoelectronically active π-conjugated units. The suppressed electronic interactions among π-conjugated units (π-π interactions) by alkyl chain wrapping, collaborating with the molten feature of alkyl chains, produce an entropy-driven fluid state at room temperature. The easily adaptable methodology and the existence of numerous π-conjugated molecules have remarkably extended the scope of current FMLs.

Precise tailoring of the bulk functions with rational molecular design is a great challenge because of the rapid but unguided development of alkylated-π FMLs. In the condensed state, random aggregation of π-conjugated units, driven by π-π interactions, always produce unexpected optical and electronic properties. For example, the molecular inherent optical character in a dilute solution can ideally be transferred equally into the solvent-free bulk liquid^8, 9^. Nevertheless, unexpected optical properties, such as emission quenching^31^ or peak shifting^10, 32^, are frequently encountered. Additionally, the liquid-physical properties can also be influenced by feeble π-π interactions. However, there is no thorough understanding of the liquid nature of FMLs because of the technical difficulty in determining the local molecular organization of the amorphous materials. Therefore, engineering weak π-π interactions in alkylated-π FMLs is essential to regulate optoelectronic functions, and understanding their impact on the liquid-physical properties may illuminate the true liquid-physical nature. Nevertheless, it is not straightforward to control and characterize these weak π-π interactions.

We devised an informative monomer-excimer dual fluorescence of pyrene^34, 35^ that can sensitively indicate the extent of interactions between two adjacent pyrene chromophores. Additionally, the fluorescence feature can be highlighted in condensed states^36–38^. A series of alkylated pyrenes, which have branched alkyl chains of different substitution patterns, is employed as a rational FML model. All these compounds are solvent-free liquids without long-range ordering at room temperature. With mature modulation on the number, length, and substituent motif of the alkyl side chains, these FMLs exhibit different fluorescence (from excimer to uncommon intermediate to monomer). The emission evolution probes a gradual weakening of the pyrene-pyrene interactions in a clear trend, from stronger to weaker to negligible. Synchronously, engineering pyrene-pyrene interactions has regulated the liquid-physical properties from inhomogeneous to isotropic. The inhomogeneity in locally stacked species is evidenced for most compounds by ultrafast time-resolved spectroscopy techniques, although the mechanism for the formation of intermediate species remains an open question that needs further studies. The discovery of these inhomogeneous FMLs using unconventional techniques nearly dispels the unclear understanding regarding the homogeneity/inhomogeneity in amorphous soft-materials, *i*.*e*., liquid matter. We believe our new approach will provide a guide to design alkylated-π FMLs with tailorable π-unit-derived optoelectronic functions and a predictable liquid nature.

## Results

### Molecular Design

**Figure 1:**
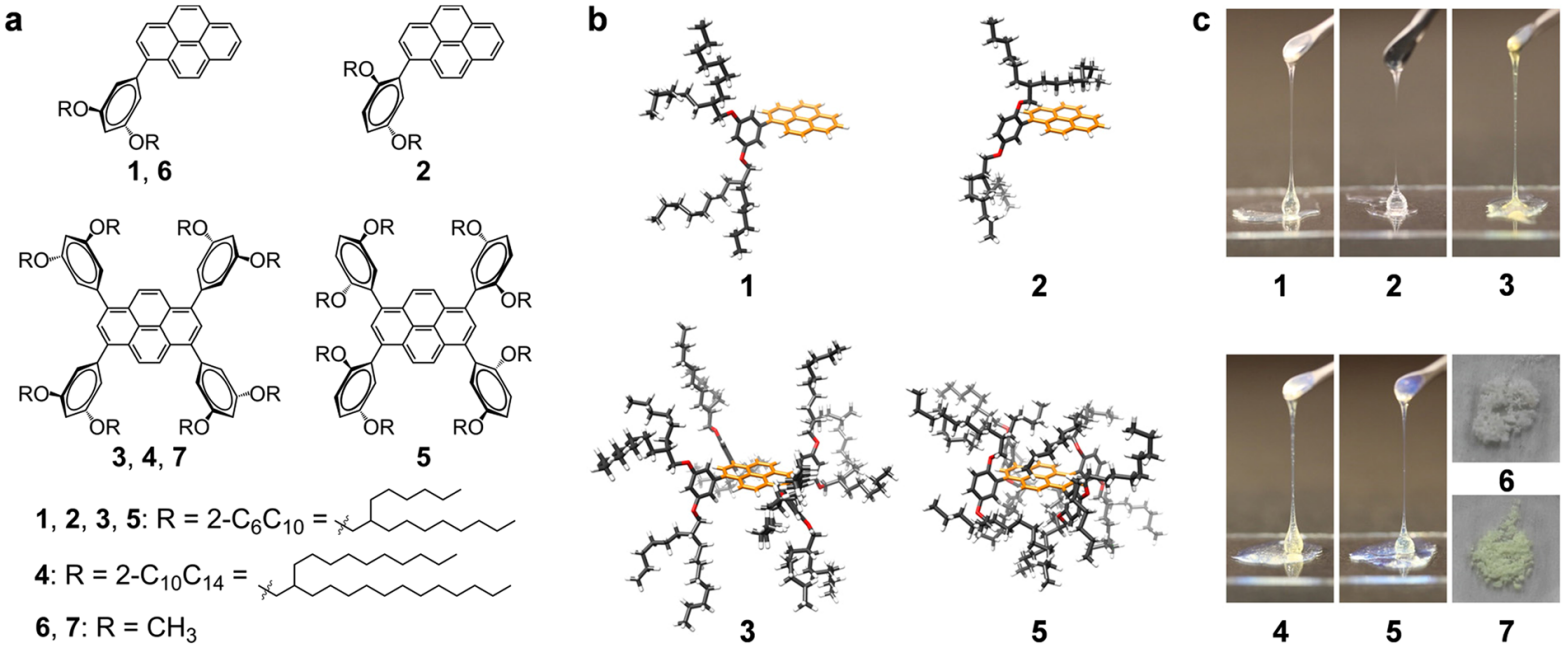
Molecular design. (**a**) Chemical structures of pyrene derivatives **1**–**7**. (**b**) Schematic drawing of 3D model structures of **1**, **2**, **3** and **5** illustrating their distinct local microenvironments at periphery of pyrene moiety. Alkyl chains inserted at 2-position on phenyl substituent unit are possibly located closer on pyrene moiety. (**c**) Photo images of fluids **1**–**5** and solid powder **6** and **7** under daylight at ambient temperature.

We prepared a series of pyrene derivatives (**1**–**5**, Fig. 1a) substituted with different Guerbet alcohol-based^39^ branched alkyl chains. The effects of the number, length, and substituent motif of the alkyl side chains were considered to gain a comprehensive understanding of the correlation between molecular structure and pyrene-pyrene interactions. Different alkyl chains are anticipated to generate distinct local microenvironments at the periphery of the pyrene moiety (Fig. 1b) and characteristic fluorescence properties. The fluorescence information can therefore probe different extents of interactions among adjacent pyrene units, whose correlation with the alkyl chain substitution pattern will be addressed.

Pyrenes **1**–**7** (Fig. 1a) were synthesized by Suzuki coupling reactions (Supplementary Figs S1–S24). **1–5** were obtained as transparent viscous fluids at room temperature, while reference compounds **6** ^40^ and **7** (with methoxy substitutions) appeared as solid powders (Fig. 1c). **5** exhibits complex and broad signals in the ^1^H and ^13^C NMR spectra (Supplementary Figs. S19 and S20), which are probably due to conformational isomerism originated from the asymmetric (2,5)-alkyl chain substitution motif on the phenyl units (Supplementary Fig. S25). The ^1^H NMR spectra and thermogravimetric analysis (TGA) (Supplementary Fig. S26) confirmed the absence of residual solvent in neat samples **1**–**5**.

### Phase Behavior

**Figure 2:**
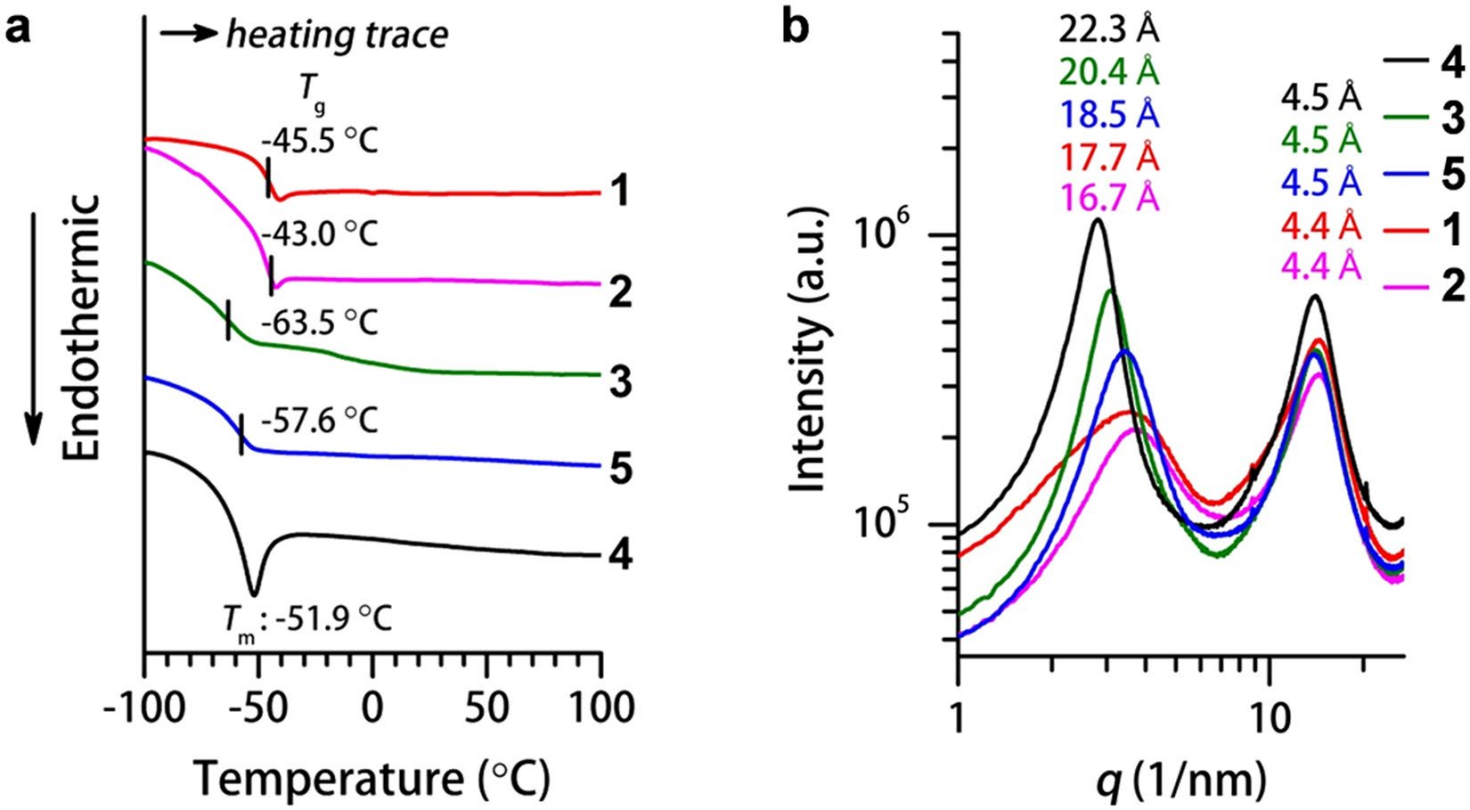
Phase properties and structural analysis of **1**–**5**. (**a**) Differential scanning calorimetry thermograms of **1** (red), **2** (pink), **3** (green), **4** (black) and **5** (blue) in the second heating trace at a scan rate of 10 °C min^−1^ showing the *glass*-to-*isotropic* transition (*T*_g_) for **1**, **2**, **3** and **5**, and melting point (*T*_m_) for **4**. (**b**) Small and wide-angle X-ray scattering profiles of **1** (red), **2** (pink), **3** (green), **4** (black) and **5** (blue) at 25 °C.

The phase transition temperatures of fluids **1**–**5** were determined by differential scanning calorimetry (DSC). **1**–**5** show glassy transitions or melting temperatures from −63.5 to −43.0 °C (Figs 2a and S27 and S28), which are different from the high melting points of solid compounds **6** (113–115 °C) and **7** (316–319 °C) (Table 1). The low phase transition temperatures of **1**–**5**, together with their high thermal stabilities indicated by TGA (Table 1, Supplementary Fig. S26), allow **1**–**5** to exist as solvent-free fluids over a wide range of temperatures (∼−40 to ∼375 °C). Notably, **4** displays a relatively clear endothermic solid-to-isotropic liquid melting transition at −51.9 °C (Figs 2a and S27). The melting (rather than glassy) transition of **4** originates from its favorable crystallization tendency, which is facilitated by the symmetric molecular skeleton and longer substituted 2-decyltetradecyl chains than the 2-hexyldecyl chains in other molecules.

**Table 1:** Physical characteristics (*T*_g_: glass transition temperature; *C*_mol_: molar heat capacity; *T*_m_: melting point; *T*_d95%_: decomposition temperature at 95% weight loss; η*: complex viscosity) of **1**–**7** in solvent-free state.

Compound	***T***_g_ (°C)^*a*^	***C***_mol_ (J mol^−1^ K^−1^)	***T***_m_ (°C)	***T***_d95%_ (°C)^*d*^	***ƞ*** * (Pa · s)^*e*^
1	−45.5	259.1	—	390	6.1
2	−43.0	100.0	—	375	2.3
3	−63.5	984.2	—	417	58.0
4	—	—^*b*^	−51.9^*a*^	406	7.5
5	−57.6	743.7	—	400	6.2
6	—	—	113–115^*c*^	305	—
7	—	—	316–319^*c*^	455	—

^a^Determined by the second heating scan of a DSC measurement. Scan rate: 10 °C min^−1^ under N_2_.

^b^Enthalpy and entropy of **4** at melting were determined as Δ*H* = 38.6 kJ mol^−1^ and Δ*S* = 174.5 J mol^−1^ K^−1^, respectively.

^c^Determined by melting point apparatus.

^d^Decomposition temperatures at which 5% weight loss occurred on TGA.

^e^Determined at angular frequency *ω* = 10 rad s^−1^ at 25 °C.

### Amorphous Feature

Polarized optical microscopy (POM) images of fluids **1**–**5** at room temperature (Supplementary Fig. S29) do not show any texture, indicating a lack of long-range ordered and assembled domains. Absence of long-range order is supported by small and wide-angle X-ray scattering (SWAXS). **1**–**5** exhibit only two broad halos (Fig. 2b), which are distinct from the sharp crystalline-like reflection peaks of relatively ordered solids **6** and **7** (Supplementary Fig. S30). The halo in the small-angle region (*q* ≈ 2.8–3.8 nm^−1^) corresponds to the distances between adjacent aromatic moieties (16.7–22.3 Å, taken from the top of the halo). The halo that appeared in the wide-angle region (*q* ≈ 14.5 nm^−1^, 4.4–4.5 Å in distance) indicates the presence of disordered molten alkyl chains. The molten feature of the alkyl chains was also confirmed by Fourier transform infrared spectroscopy (FTIR) (Supplementary Fig. S31). The average distance between aromatic moieties is larger for **3**–**5** (18.5–22.3 Å) than **1** and **2** (16.7–17.7 Å) because the increased number of bulky alkyl chains in **3**–**5** fill the spaces between neighboring aromatic units. A closer examination on the small-angle region reveals broader aromatic halos for **1** and **2** than **3**–**5** (Supplementary Table S1), which can be ascribed to the asymmetric molecular skeleton of **1** and **2**. Asymmetric molecular structures generate more conformations and greater entropy, and could display broader aromatic halos. Correspondingly, **5** has an asymmetric structure on the (2,5)-alkyl chain substituent motif and exhibits a broader aromatic halo than **3** and **4**.

The random orientation of pyrene cores and the absence of long-range ordered structures in **1**–**5** are further evidenced by flash-photolysis time-resolved microwave conductivity (FP-TRMC)^41^. FP-TRMC is an advanced technique capable of evaluating the nanometer-scale mobility of charge carriers. According to FP-TRMC profiles (Supplementary Fig. S32), **1–5** show pseudo-photoconductivity maxima on the order of 10^−5^–10^−4^ cm^2^ V^−1^ s^−1^ and a prompt deactivation faster than 0.3 μs. These values are considerably lower than those reported for rubrene crystals (10^−3^ cm^2^ V^−1^ s^−1^, >10 μs)^42^ and liquid crystalline compounds (*e*.*g*., fused oligothiophenes, 10^−4^ cm^2^ V^−1^ s^−1^, >10 μs)^43^ using the same technique. DSC, POM, SWAXS, FTIR, and FP-TRMC analyses all indicate that **1**–**5** are amorphous fluids with random molecular orientations at room temperature, even though they have different alkyl chain substituent styles.

### Liquid Physical Properties

**Figure 3:**
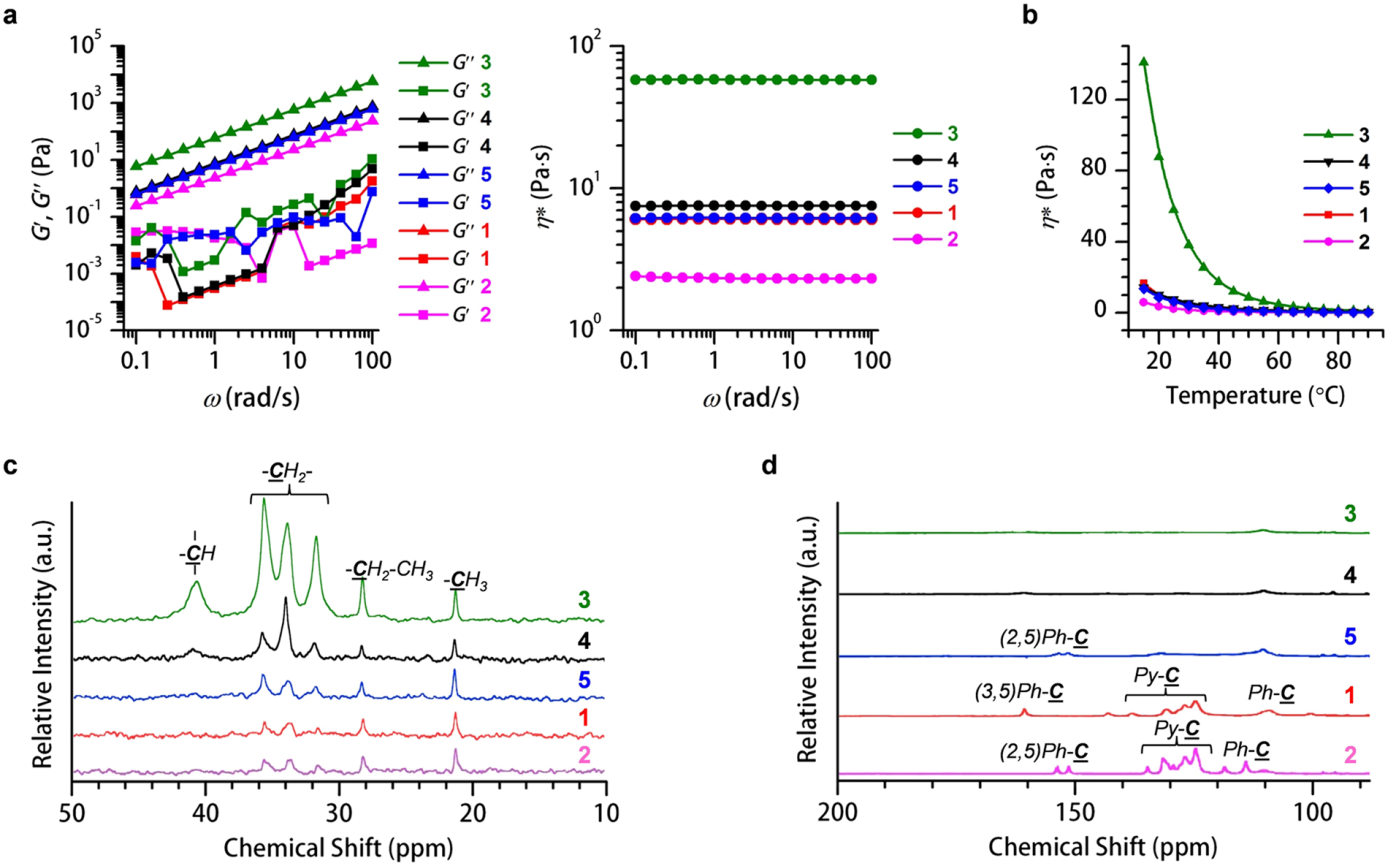
Liquid-physical properties of **1**–**5**. (**a**) Variation of storage elastic modulus (*G*′, square), viscous loss modulus (*G*″, triangle) (left) and complex viscosity (ƞ*, circle) (right) as a function of angular frequency (*ω*) for neat samples of **1** (red), **2** (pink), **3** (green), **4** (black) and **5** (blue) with a strain amplitude (*γ*) = 0.25 at 25 °C. The viscosities are ordered as: **3** > **4** > **5** ≥ **1** > **2**. (**b**) ƞ* as a function of temperature. *γ* = 0.25, *ω* = 10 rad s^−1^. Solid-state ^13^C NMR spectra, (**c**) CP-MAS and (**d**) DD-MAS mode of **1** (red), **2** (pink), **3** (green), **4** (black) and **5** (blue) measured at 27 °C. The intensity of CP-MAS follows an order of **3** > **4** > **5** ≥ **1** > **2** while the intensity of DD-MAS is on an order of **3** < **4** < **5** < **1** < **2**.

The rheological behaviors of **1**–**5** were studied to investigate their fluid properties. All fluids exhibit a higher viscous loss modulus (*G*″) than storage elastic modulus (*G*′) throughout the measured angular frequency (*ω*) (Fig. 3a, left) and amplitude (*γ*) sweeps (Supplementary Fig. S33), demonstrating liquid-like behaviors. Moreover, their viscosities decreased after heating (Fig. 3b), which is another representative feature for liquid matter. **1**–**5** are regarded as Newtonian-type liquids because their complex viscosity (η*) is independent of *ω* (Figs 3a and right) and *γ* (Supplementary Fig. S34).

Liquids **3**–**5** (attached by eight alkyl chains) exhibit higher values of η* than **1** and **2**, which are appended with two alkyl chains (Fig. 3a and Table 1). Therefore, alkyl chains are dominant in determining the liquid viscosity. Generally, the outstretched alkyl chains of the (3,5)-substituent motif on the phenyl unit facilitate intermolecular alkyl chain friction. Whereas the (2,5)-motif, with the 2-substituted alkyl chain extending inward to the pyrene core and 5-substituted alkyl chain stretching outward (Fig. 1b), suffer less intermolecular alkyl chain friction. As a result, **1** (or **3** and **4**) ((3,5)-motif) exhibits a higher value of η* than **2** (or **5**) ((2,5)-motif). Notably, the difference in η* between **1** (or **3**) and **2** (or **5**) is also influenced by richer pyrene-pyrene interactions in **1** (or **3**) than **2** (or **5**) (*vide infra*).

Solid-state NMR (SSNMR) is a powerful tool to characterize molecular dynamics and local chemical environments for rigid solid substances^44^ and soft biomolecules^45^. We used SSNMR to inspect the microphysical properties of FMLs for the first time. Two different SSNMR techniques were used: cross polarization-magic angle spinning (CP-MAS)^46^, which is favorable for rigid samples (10^−2^–10^−4^ s in molecular motion), and single pulse excitation with proton decoupling-MAS (DD-MAS)^47^, which is appropriate for mobile samples (10^−5^–10^−7^ s in molecular motion). When applying CP-MAS, the most viscous liquid, **3**, shows the strongest signals (Fig. 3c). In contrast, when DD-MAS is adopted, lower viscosity liquids **1** and **2** exhibit sharper signals (Fig. 3d). Both CP-MAS and DD-MAS outputs agree with the order of η* (**3** > **4** > **5** ≥ **1** > **2**) determined by rheological studies (Fig. 3a and Table 1).

The liquid-physical properties determined by rheology and SSNMR suggest that alkyl chain friction is vital for mastering molecular motions in alkylated-π FMLs. Indeed, pyrene-pyrene interactions also contribute to liquid-physical properties (described in the section of excited-state dynamics).

### Steady-State Photophysical Properties

**Figure 4:**
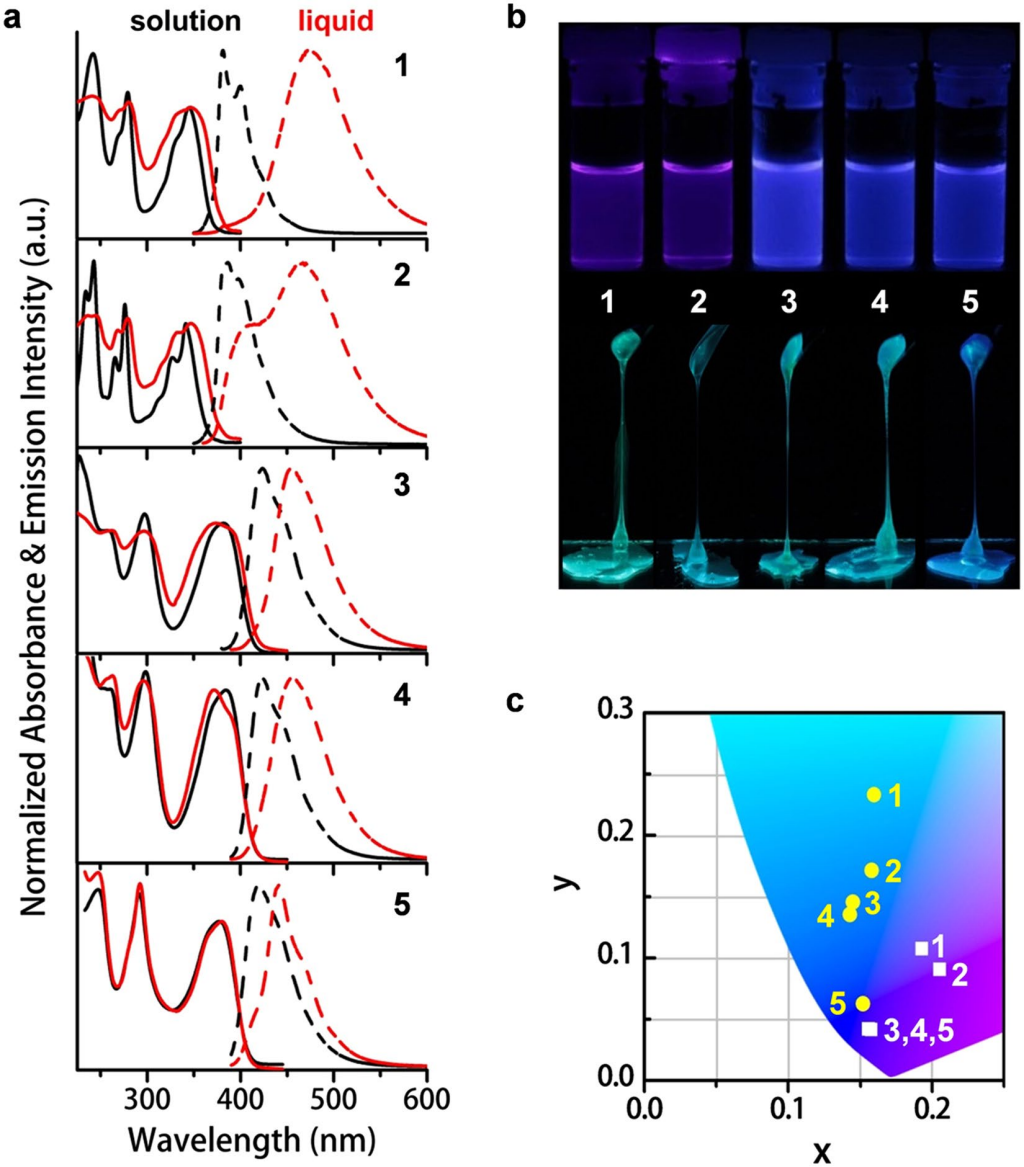
Photophysical properties of **1**–**5** in solution and solvent-free liquid state. (**a**) Normalized UV-vis absorption (solid lines) and fluorescence (dashed lines) spectra of **1**–**5** in solution (black) (UV-vis: 10 μM; fluorescence: 1 μM) and solvent-free liquid state (red) at 25 °C. Solvent: **2**, *n*-hexane; others, dichloromethane. The liquid samples were sandwiched between two quartz plates. *λ*_ex_: **1** and **2**, 344 nm; **3**, **4** and **5**, 378 nm. (**b**) Photo images of solution (top) and liquid (bottom) samples under handy UV lamp irradiation at 365 nm. (**c**) A section of CIE 1931 chromaticity diagram of **1**–**5** in both solution (white squares) and liquid state (yellow circles) at 25 °C. Only liquid **5** exhibits similar luminescent color to its solution, while liquids **1**–**4** showed different emission colors to their solutions.

Steady-state UV-vis absorption and fluorescence spectroscopies of **1**–**5** in dilute solutions and solvent-free liquids were utilized to elucidate a clear relevance between molecular structure and pyrene-pyrene interactions. In solution, all samples exhibit sharp, intense, and vibronic monomeric spectral feature (Fig. 4a and Supplementary Table S2). The more twisted conformation of **2** (or **5**) (Supplementary Fig. S35) slightly blue shifts the spectra with respect to that for **1** (or **3**). Unlike its sharp absorption bands in solution, **1** displays prominently broadened spectra in the liquid state. The broadening effect implies the existence of random pyrene-pyrene interactions in the ground state to different extents. Evidently, the attachment of two branched alkyl chains in **1** is not bulky enough to hamper pyrene-pyrene interactions in its condensed liquid state. As approved by fluorescence spectroscopy, liquid **1** exhibits a characteristic feature of excimer emission, *i*.*e*., a broad, structureless, and 90-nm red-shifted band (375–600 nm; ${\lambda }_{{\rm{\max }}}^{{\rm{em}}}$ = 472 nm) compared to that in the monomeric solution (375–500 nm; ${\lambda }_{{\rm{\max }}}^{{\rm{em}}}$ = 382 nm). The excimer nature can be further proven by coincident fluorescence features of liquid **1** (Fig. 4a) with compound **6** ^48^ (Supplementary Fig. S36a).

Suppression of pyrene-pyrene interactions occurs by increasing the number of steric phenyl units and bulky alkyl chains. **3** possesses eight alkyl chains and exhibits a broader absorption spectrum in liquid than in solution (Fig. 4a). However, the broadening effect is less significant than for liquid **1** (with two alkyl chains). This indicates that liquid **3** has weaker pyrene-pyrene interactions in the ground state. As reflected in the fluorescence spectra, **3** displays a less broadened emission band and a smaller red-shifted peak from solution (390–550 nm; ${\lambda }_{{\rm{\max }}}^{{\rm{em}}}$ = 424 nm) to liquid (390–600 nm; ${\lambda }_{{\rm{\max }}}^{{\rm{em}}}$ = 456 nm) than **1**. A similar fluorescence spectral red shift, from solution to solid, is observed for **7** (Supplementary Fig. S36a) and its analogs^49^.

The length of the alkyl side chains in **3** was increased from 2-hexyldecyl to 2-decyltetradecyl to further weaken the pyrene-pyrene interactions. However, the resulting liquid, **4**, shows similar fluorescence spectral features to those of **3**. Therefore, pyrene-pyrene interactions are not weakened by increasing the chain length on the (3,5)-motif in the same molecular skeleton.

In contrast, by altering the substituent position of alkyl chains from the (3,5)- to (2,5)-motif on the phenyl units of **3**, the resulting liquid, **5**, exhibits an almost identical absorption spectrum to that for its solution. Its sharp and vibronic emission band in the liquid (390–550 nm; ${\lambda }_{{\rm{\max }}}^{{\rm{em}}}$ = 441 nm) is comparable to that in solution (390–550 nm; ${\lambda }_{{\rm{\max }}}^{{\rm{em}}}$ = 419 nm). Although there is a 22-nm shift in the fluorescent band peaks from solution to liquid, both spectra exhibit vibronic sub-peaks with positions around 420, 443, and 470 nm. The coincidence of the positions supports that **5** exists as monomers in neat liquid. The red shift of the emission peak is caused by changes in the pattern of vibronic structures from solution to liquid, as indicated by the absence of mirror symmetry of the neat-state fluorescence spectrum with respect to the absorption spectrum. The less clear sub-peaks in the solution spectrum than those in the liquid indicate more vibronic structures exist for **5** as a liquid than in solution. The different vibronic structures can be attributed to different surroundings of the pyrene unit, *i*.*e*., solvent in solution *vs*. liquid molecules in the neat state. Therefore, both the absorption and emission spectra of liquid **5** have monomeric character. In other words, the pyrene core in **5** is well shielded by surrounding alkyl chains, and the pyrene-pyrene interactions are efficiently hampered. The better isolation of the pyrene core by the (2,5)-motif than the (3,5)-motif is also manifested by a spectral comparison between **2** and **1**. Compared to **1**, liquid **2** shows less broadening in the absorption spectrum than that in solution. The fluorescence spectrum of liquid **2** can be distinguished as a mixture of monomer (398 nm) and excimer (467 nm) bands. Therefore, the pyrene-pyrene interactions in **2** are partially suppressed. The suppression is derived from steric effects of the alkyl chains substituted at the 2-position on the phenyl unit, which can be located closer to the pyrene surface (Fig. 1b). Proof of the intramolecular closeness was given by the ^1^H NMR spectra (Supplementary Fig. S37). Protons on the alkyl chains at the 2-position on the phenyl unit of **2** and **5** are well shielded by the ring current of the pyrene unit, even in solution.

Liquids **1**–**5** show high values of *Φ*_FL_ (57–85%) (Supplementary Table S2) and display individual bluish fluorescence (Fig. 4b and c). The fluorescence color is quite stable and reliable because of the amorphous features. From this viewpoint, these alkylated liquid-pyrenes are advantageous over solid^49–51^ and liquid crystalline^52, 53^ polymorphic pyrenes whose fluorescence color is sensitive to the molecular packing structure in different metastable states (Supplementary Fig. S36). In addition, the characteristic fluorescence features of **1**–**5** (excimer (**1**), mixture of excimer and monomer (**2**), intermediate (**3** and **4**), and monomer (**5**) emissions) have well probed the extents of pyrene-pyrene interactions (with an order of **1** > **2** > **3** ≈ **4** > **5**). The direct correlation between molecular structure and the extent of pyrene-pyrene interactions is clarified because the emission properties are finely regulated with different alkyl chain substituent patterns.

### Excited-State Dynamics

**Figure 5:**
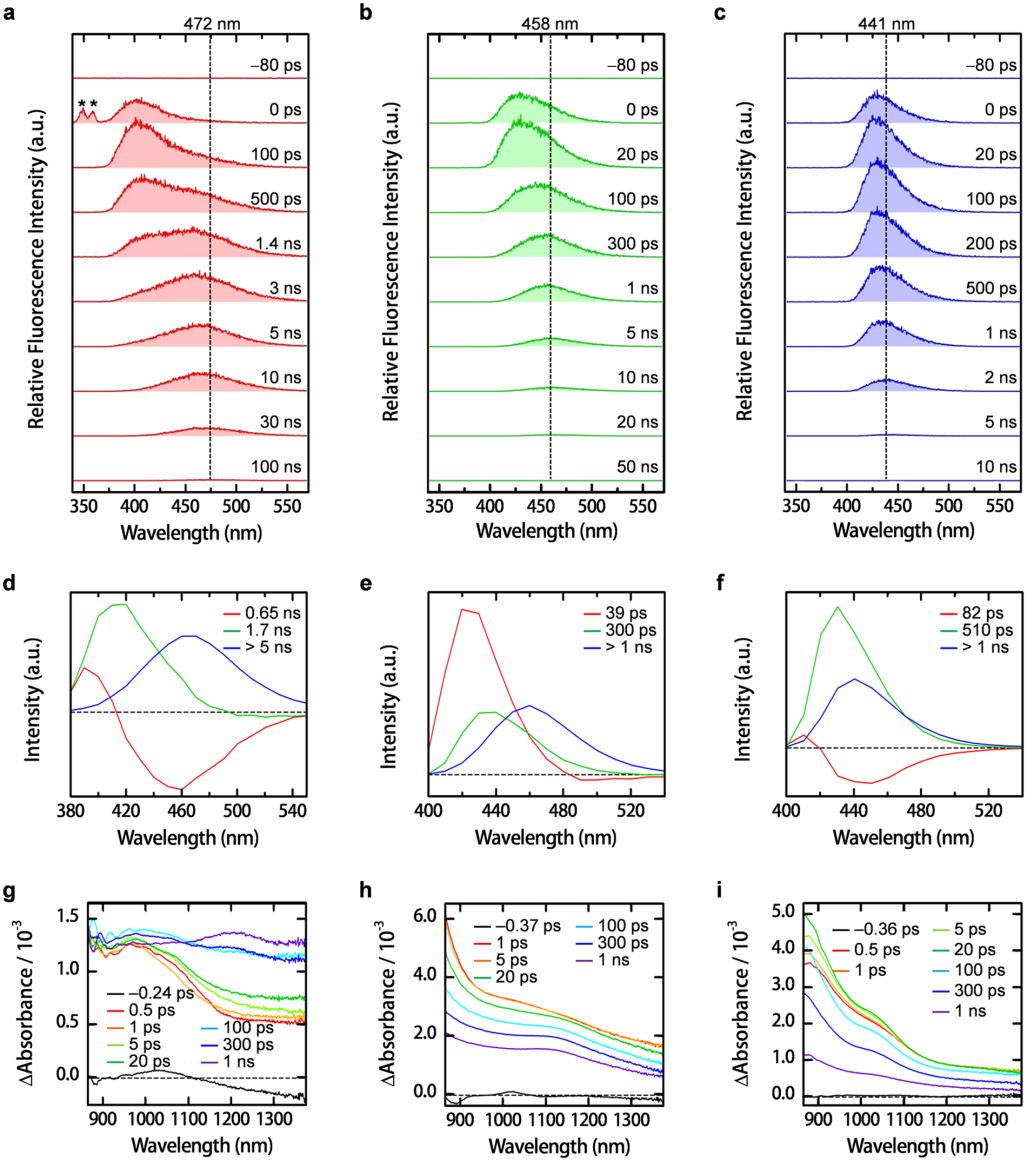
Excited-state dynamics of liquids **1**, **3** and **5**. Picosecond time-resolved fluorescence spectra of liquids **1**, **3** and **5** in (**a**,**b** and **c**) at time delays as denoted insets. Asterisks in (**a)** (at 0 ps) denote the stray light signals caused by the excitation pulse. *λ*_ex_: **1**, 345 nm; **3**, 378 nm; **5**, 382 nm. Decay-associated spectra of **1**, **3** and **5** in (**d**,**e** and **f**) obtained by global least-squares fitting analysis with three exponential functions. Femtosecond time-resolved near-IR absorption spectra of liquids **1**, **3** and **5** in (**g**,**h** and **i**) for at time delays as denoted insets. *λ*_ex_: **1**, 345 nm; **3** and **5**, 380 nm. Pulse duration, ~100 fs.

Two ultrafast spectroscopic experiments were performed to gain deeper insights into the initial photophysics of liquid pyrenes. First, picosecond TRFL spectroscopy with a streak camera was employed to observe the excited-state dynamics in the picosecond to sub-nanosecond regime. The temporal fluorescence spectral evolution of **1** (Fig. 5a) shows a slow peak shift from 400 to 472 nm within nanoseconds, demonstrating the formation of excimers from excited monomers. The excimer fluorescence decays with a smaller time constant than that obtained from the nanosecond TRFL measurement. The difference is caused by a higher excitation intensity applied to picosecond TRFL measurements, which may cause singlet-singlet annihilation in the nanosecond time regime. When superimposed from 0.4–4.0 ns, the picosecond TRFL spectra reveal that only part of the monomers form excimers. The time constant for the formation of excimers is 0.65 ns, as evidenced by the spectral evolution with an isoemissive point (Supplementary Fig. S38a). The rest of the monomers directly decay without contributing to the excimer formation from 1.4–4.0 ns (Supplementary Fig. S38b). The observed spectral changes are well reproduced by the global least squares fitting analysis with three exponential functions from 380–550 nm (Figs 5d and S39a). The decay-associated spectrum with the smallest time constant (0.65 ns) is composed of positive (380–415 nm) and negative (415–540 nm) components. The former suggests a decay of monomers, whereas the latter indicates the formation of excimers. The spectrum with the second smallest time constant (1.7 ns) is assigned to the decay of monomers without forming excimers because it has an almost identical band shape to that of the monomers. The spectrum with the largest time constant (>5 ns) is assigned to the decay of excimers.

The excimer formation dynamics was clearly observed using femtosecond time-resolved near-IR (TRNIR) spectroscopy. An absorption band characteristic of photoexcited pyrene monomers^54^ is observed 0.5 ps after the photoexcitation in the near-IR region around 1000 nm, with a broad feature between 1150 and 1400 nm (Fig. 5g). When most of the monomer absorption decays, a broad and structureless absorption band of pyrene excimers^55, 56^ gradually increases with a time constant of 120 ps. Though the time constant does not fully agree with the result of the picosecond TRFL measurement because of the large pump pulse energy for the TRNIR measurement, the TRNIR spectra support fast excimer formation in neat liquid **1**.

**Figure 6:**
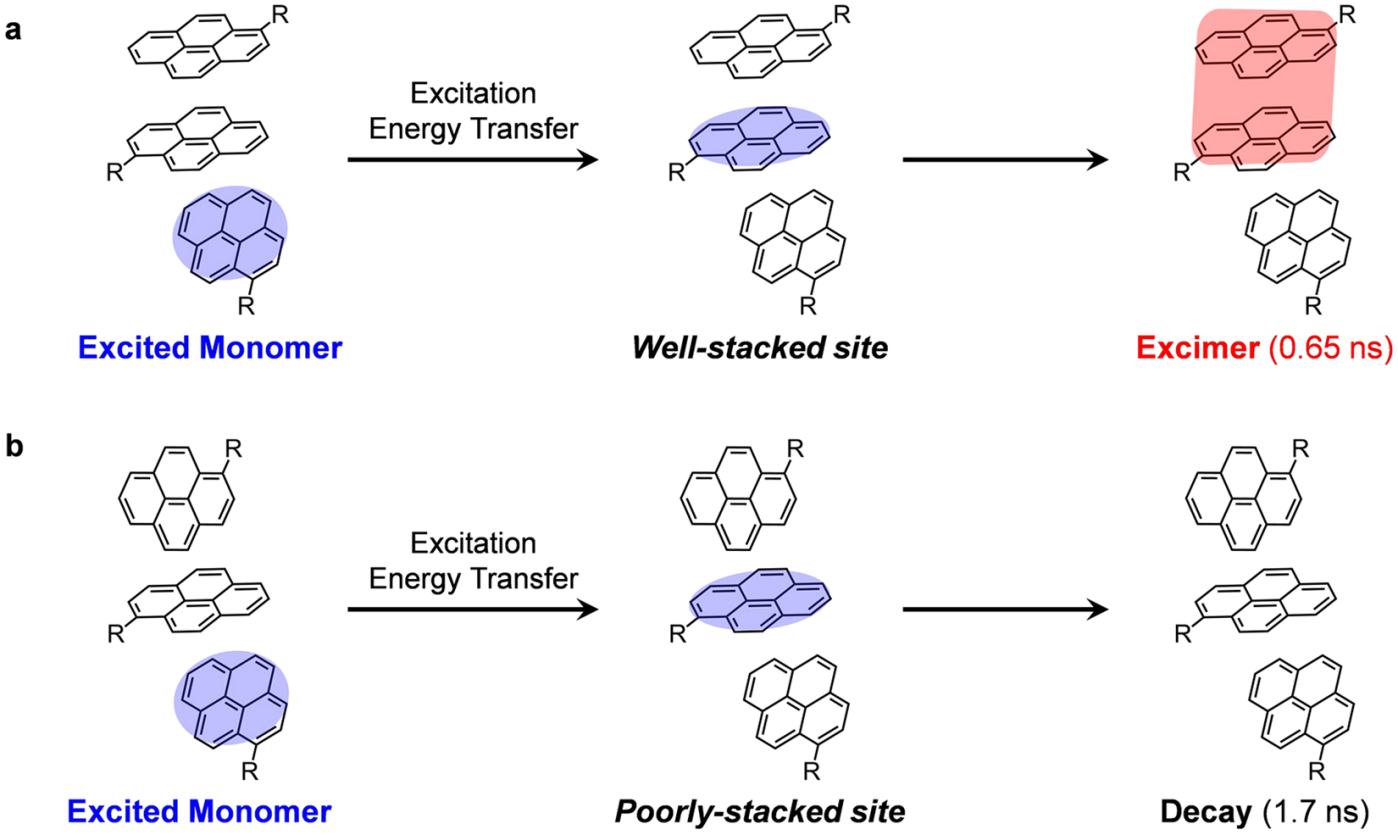
Mechanism for excitation energy transfer of liquid **1**. (**a**) A proposed diagram illustrating the formation an excimer from a ground-state monomer and its well-stacked excited-state monomer formed through excitation energy transfer (EET). (**b**) A proposed diagram illustrating the decay of an excited monomer without forming an excimer.

The excimer formation in liquid **1** (time constant of 0.65 ns) is much faster than that formed through conventional diffusion-controlled reactions. For pyrene in solutions, excimers are formed through collisions between excited and ground state monomers during their rotational and translational diffusions. However, in neat liquid the high viscosity prevents molecules from the rotational and translational motions in the nanosecond time scale. Even if these motions are restricted, excitations can still promptly diffuse by excitation energy transfer (EET) through dipole coupling. EET could occur multiple times before excimer formation or monomer decay. Therefore, within the monomer lifetime, an excimer can form if the excitation energy is transferred to a well-stacked molecule in a face-to-face configuration (Fig. 6a). Once the excitation energy reaches that of a molecule associated with its neighbor in another configuration, the associated species may decay with extremely short lifetimes or the molecule itself directly decays as a monomer (Fig. 6b). The global analysis indicates that only the monomer and excimer bands can be retrieved. The mechanism is also supported by temperature-dependent spectroscopic studies. A blue shift in the emission spectra occurred upon cooling **1** (Supplementary Fig. S40a). With decreased temperature, the number of configurations favorable for excimer formation decreases. Therefore, the excimer formation is somewhat suppressed.

The relative amplitude of the two monomer components at 380 nm (Fig. 5d), where monomer fluorescence is predominantly observed, indicates that around 50% of excited monomers are converted to excimers. This result excludes the hypothesis that excimers form predominantly from direct photoexcitation of pre-stacked structure in the ground state. If excimers were created in this way, their high yield (50%) would suggest a large fraction of pre-stacked structure in **1**. However, the SWAXS result for **1** (Fig. 2b) indicates absence of specific stacked pyrene structures and the coexistence of many different conformations in the ground state. In contrast, the EET mechanism explains the yield of excimers that are consistent with the results of SWAXS and other measurements. Therefore, EET is reasonable to illuminate the formation of excimer in **1**.

Liquid **5** exhibits a much smaller peak shift from 430 to 440 nm and more negligible band broadening in the picosecond TRFL spectra than **1** and **3** (Figs 5c and S41). Global analysis gives three exponential functions that are well fitted to the time evolution between 400 and 540 nm (Figs 5f and S39c). The decay-associated spectrum with a time constant of 82 ps shows a negative component, which is assignable to monomers instead of excimers. The assignment is supported by the similar band shape of the two decay spectra with time constants of 510 ps and >1 ns. Moreover, femtosecond TRNIR spectroscopy also shows the absence of spectral changes from 0.5 ps to 1 ns (Fig. 5i). The negative component is probably due to very weak associations between the monomers and their adjacent pyrene cores through dipole coupling. Therefore, the excited molecules in neat liquid **5** hold the monomer character.

The picosecond TRFL spectra of **3** exhibit a 32-nm peak shift from 426 to 458 nm within 300 ps of photoexcitation (Fig. 5b). The TRFL spectra can be well reproduced by three decay-associated spectra with time constants of 39 ps, 300 ps, and >1 ns (global least squares fitting; Figs 5e and S39b). No spectra contain negative components that correspond to the rise of a transient. The spectra with time constants of 39 and 300 ps are assigned to monomers, which likely interact with adjacent pyrene cores at different magnitudes. The spectrum with a time constant >1 ns is ascribed to intermediate species between monomers and excimers. These intermediate species form within the instrument response time of the picosecond TRFL spectrometer, *i*.*e*., 20 ps (Supplementary Fig. S42). Such an ultrafast formation is experimentally detected as a difference in the spectral shape between **3** and the other compounds at 0 ps (Supplementary Fig. S43). Thus, the 32-nm red shift in Fig. 5b can be explained by the decay of monomers.

An unambiguous assignment of the intermediate is difficult from the time-resolved fluorescence and near-IR spectra. TRNIR spectra do not show a significant change in 1 ns (Fig. 5h), while the monomer fluorescence almost completely decays in the same time range. A characteristic absorption of excimers, as indicated by those of **1**, is not observed. The absence of a large spectral change implies that the intermediate species are excited monomers interacting strongly with surrounding molecules. The strong intermolecular interaction may promote the formation of an unusual aggregate structure that significantly lowers energy of excited states. The presence of the aggregate is further evidenced by the lower stability of the ground-state-dimers. Upon heating, there is an obvious change in the shape of the absorption band, and the emission peak blue-shifted by 16 nm for liquid **3** (Supplementary Fig. S40c). The final spectra agree with the monomeric spectra of **3** in solution (Fig. 4a), suggesting a heat-induced dissociation of weakly aggregated structures into monomers. In contrast, liquids **1** and **5** maintained a similar spectral shape before and after heating (Supplementary Fig. S40b and d).

Such an intermediate emission of pyrene is seldom discovered in solution. It is occasionally reported for pyrenes in the solid state, but it is generally described as monomer emission^49, 57^. Here, we propose the origin of the pyrene intermediate emission. We hypothesize that the metastable intermediate species can be immobilized and stabilized by a high-viscosity, solvent-free liquid. Thus, the uncommon emission of neither monomers nor conventional excimers can be captured. Otherwise, in solution, the high molecular mobility facilitates quick re-orientation of these species. Thus, they may either form excimers or revert to monomers.

Excited-state dynamics confirms the origin of emission features for liquids **1**–**5** and provides solid evidence for the particular emitting species in each liquid. All liquids, except **5**, contain various emitting species. Liquid **1** includes molecules stacked with distinct configurations, which decay as either monomers or excimers. Similarly, liquid **2**, exhibiting a mixture of monomer and excimer bands, would consist of different local species. Liquids **3** and **4** contain both monomers and ground-state-dimers. Therefore, liquids **1**–**4** are intrinsically inhomogeneous. Liquid **5**, as an excellent proof-of-concept, consists solely of monomers and behaves homogeneously as an isotropic liquid. Indeed, there have been continuous arguments on the physical category of FMLs because it is difficult to characterize their local molecular organization using conventional structural analysis. Our concerted utilization of ultrafast time-resolved spectroscopy and molecular motion dynamics provides the first deep insights into the physical nature of FMLs. Liquids **1**–**4** contain randomly stacked, locally “inhomogeneous” species in the absence of long-range ordered molecular organization. These inhomogeneous liquids represent an uncommon fluid matter that may find a space between relatively disordered nematic liquid crystals and totally disordered isotropic liquids. Since the inhomogeneity comes from the subtle π-π interactions in these alkylated-π FMLs, substances with sufficiently promoted π-π interactions could preferably form LC phases^52, 53, 58^, whereas those with significantly frustrated π-π interactions could behave in an isotropic manner, as for **5**.

## Discussion

A series of alkylated-pyrenes, with a pyrene unit bearing flexible and bulky branched alkyl chains of different numbers, chain lengths, and substitution positions were synthesized. All compounds behave as solvent-free Newtonian-type liquids at room temperature. Despite the lack of long-range ordered pyrene-assembly, most of these liquids are intrinsically inhomogeneous with distinct local pyrene-pyrene associations. The inhomogeneity, evidenced firmly by ultrafast time-resolved spectroscopy techniques, has advanced a better understanding on the true physical nature of FMLs. Additionally, our strategy for finding evidence of microscopic inhomogeneity has cast a new light on characterizing the local molecular arrangement, a long-standing challenge for amorphous materials. Thorough investigation of the photophysical properties well interrelate alkyl chain substituent patterns and the trend of pyrene-pyrene interactions. Both the alkyl chain number and substituent motif play crucial roles in engineering the interactions among functional π-units and regulating liquid-physical properties and π-unit-derived optoelectronic functions. To equally transfer molecular inherent properties to a bulk liquid, *i*.*e*., sufficiently isolated π-conjugated units with negligible π-π interactions, a certain number of flexible and bulky branched alkyl chains with the (2,5)-motif is recommended. As demonstrated by liquid **5**, a stable, reliable, and predictable blue-luminescent color with ultrahigh purity and uniformity independent of conditions is guaranteed. Fewer alkyl chains with the (3,5)-motif are favored to design liquids with properties derived from relatively strong π-π interactions. Our attempt for understanding the influence of molecular structure on the trend of π-π interactions and physicochemical properties provides a valid guide to design alkylated-π FMLs with predictable liquid-physical properties and tailorable optoelectronic functions. This study will lead to innovative liquid chemistry and promote profitable development of liquid-based advanced materials with well-tailored functions. Moreover, our artful regulation on the distance and relative orientation between π-conjugated units may provide an efficacious strategy for designing excited-state energy transfer in biological photosynthetic systems.

## Methods

### Solid-state NMR measurements

Solid (neat)-state NMR measurements were carried out on a Bruker Avance III 600 MHz spectrometer (Varian, Palo Alto, CA), with around 50 mg of liquid sample filling into a zirconia rotor of 4.0-mm outer diameter which was tightly sealed with a glass rod as a cap. ^13^C NMR spectra were recorded at the resonance frequencies of 150.1 MHz and the chemical shifts were externally referenced to 176.03 ppm of glycine carbonyl carbon with respect to tetramethylsilane (0.0 ppm). Both cross polarization-magic angle spinning (CP-MAS) and single pulse excitation with dipolar decoupling-magic angle spinning (DD-MAS) were used to record ^13^C NMR spectra. Typically, 10000 transients were accumulated and 10 Hz of exponential line broadening function was applied to Fourier transformation. The spinning frequency was set to 10 kHz and the duration of 90° pulse for the observed ^13^C nucleus was 5.0 us. In CP-MAS and DD-MAS experiments, two pulses phase modulation (TPPM) proton decoupling^59^ was used, and the contact and repetition times were 1 ms and 4 s, respectively.

### Picosecond time-resolved fluorescence measurements

Picosecond time-resolved fluorescence spectra were measured with a lab-built time-resolved fluorimeter using a femtosecond laser and a streak camera^60^. The excitation pulse was generated using an optical parametric amplifier (Coherent, OPerA Solo) pumped by an amplified Ti:sapphire laser (Coherent, Micra-5/Legend Elite-USP, 800 nm, 30 fs, 1 kHz). The excitation wavelength was set at 345, 378, and 382 nm for the compounds **1**, **3**, and **5**, respectively. The pulse energy of the excitation pulse was adjusted to be 50 pJ. Fluorescence from the sample was detected with a spectrograph (Princeton Instruments, SP-2356) and a streak camera (Hamamatsu Photonics, C10627) after it passes through a polarization analyzer and a polarization scrambler. The polarization analyzer was set at 54.7° with respect to the polarization of the excitation pulse for eliminating the effects of rotational relaxation. The sample was held between two quartz plates and translated to different spots during the measurements for avoiding accumulation of photodamaged species. Typical time resolution of the fluorimeter was estimated to be 20 ps.

### Femtosecond time-resolved near-IR absorption measurements

Femtosecond time-resolved near-IR absorption measurements were carried out using a lab-built time-resolved near-IR absorption spectrometer based on the pump-probe technique^61^. The pump pulse was generated using an optical parametric amplifier (Coherent, OPerA) pumped by an amplified Ti:sapphire laser (Coherent, Vitesse/Legend Elite-HE, 800 nm, 100 fs, 1 kHz). The excitation wavelength was set at 345 nm for the compound **1** and 380 nm for the compounds **3** and **5**. The samples were irradiated with the pump pulse with pulse energy of 0.5 μJ and then with the probe pulse after a variable time delay from the pump pulse. Intensity changes of the probe pulse induced by transient absorption were detected using a spectrograph (HORIBA Jobin Yvon, iHR320) and an InGaAs array detector (HORIBA, Symphony IGA, 512 channels). The polarization of the probe pulse was set at 54.7° with respect to the polarization of the pump pulse for eliminating the effects of rotational relaxation. The sample was held between two quartz plates and translated to different spots during the measurements. Typical time resolution of the spectrometer was estimated to be 200 fs.

All other experimental details, including materials, detailed synthesis and characterizations, techniques used and details of model fitting are provided in the Supplementary Methods.

## Electronic supplementary material

Supplementary Information
