# Supplementary material for: A Guide to Design Functional Molecular Liquids with Tailorable Properties using Pyrene-Fluorescence as a Probe
Source: Sci Rep. 2017 Jun 13;7:3416. doi: 10.1038/s41598-017-03584-1 (PMC5469858; doi:10.1038/s41598-017-03584-1)
Supplement: Supplementary file 1 — Supplementary Information [file 41598_2017_3584_MOESM1_ESM.pdf]

Supplementary Information

A Guide to Design Functional Molecular Liquids with Tailorable Properties using Pyrene-Fluorescence as a Probe

Fengniu Lu[1], Tomohisa Takaya[2,*], Koichi Iwata[2], Izuru Kawamura[3], Akinori Saeki[4], Masashi Ishii[5], Kazuhiko Nagura[6], Takashi Nakanishi[1,*]

[1]Frontier Molecules Group, International Center for Materials Nanoarchitectonics (WPI-MANA), National Institute for Materials Science (NIMS), 1-1 Namiki, Tsukuba 305-0044, Japan

[2]Department of Chemistry, Faculty of Science, Gakushuin University, 1-5-1 Mejiro, Toshima-ku, Tokyo 171-8588, Japan

[3]Graduate School of Engineering, Yokohama National University, 79-5 Tokiwadai, Hodogaya-ku, Yokohama 240-8501, Japan

[4]Department of Applied Chemistry, Graduate School of Engineering, Osaka University, 2-1 Yamadaoka, Suita, Osaka 565-0871, Japan

[5]Materials Advanced Database Group, Materials Data Platform Center, Research and Services Division of Materials Data and Integrated System (MaDIS), NIMS, 1-2-1 Sengen, Tsukuba 305-0047, Japan

[6]International Center for Young Scientists, NIMS, 1-2-1 Sengen, Tsukuba 305-0047, Japan

*E-mail: tomohisa.takaya@gakushuin.ac.jp (TT), nakanishi.takashi@nims.go.jp (TN)

Table of Contents

1. Supplementary Methods **2**
1.1 Materials 2
1.2 Synthesis and Characterization 2
1.2.1 Synthesis of **1c**, **2c** and **4c** 2
Supplementary Figures 1–6 NMR spectra 5
1.2.2 Synthesis of **1** and **2** 8
Supplementary Figures 7–12 NMR spectra and MALDI-TOF MS 9
1.2.3 Synthesis of **3**, **4**, **5** and **7** 12
Supplementary Figures 13–24 NMR spectra and MALDI-TOF MS 13
1.2.4 NMR discussion 22
Supplementary Figure 25 20
1.3 Techniques 21
2. Supplementary Tables **23**
Supplementary Table 1 23
Supplementary Table 2 24
Supplementary Table 3 25
Supplementary Table 4 25
Supplementary Table 5 26
Supplementary Table 6 27
3. Supplementary Figures **29**
Supplementary Figure 26 29
Supplementary Figure 27 29
Supplementary Figure 28 30
Supplementary Figure 29 31
Supplementary Figure 30 31
Supplementary Figure 31 32
Supplementary Figure 32 33
Supplementary Figure 33 34
Supplementary Figure 34 35
Supplementary Figure 35 36
Supplementary Figure 36 37
Supplementary Figure 37 38
Supplementary Figure 38 39
Supplementary Figure 39 39
Supplementary Figure 40 40
Supplementary Figure 41 41
Supplementary Figure 42 41
Supplementary Figure 43 42
4. Supplementary References **43**

1. Supplementary Methods

1.1 Materials

All starting materials and reagents, unless otherwise specified, were purchased from commercial suppliers (where noted) and used without further purification. 1-Bromo-2-hexyl-decane (**BrC$_6$C$_{10}$**)[1], 1-bromo-2-decyl-tetradecane (**BrC$_{10}$C$_{14}$**)[1], 5-bromo-1,3-benzenediol[2,3], 1-(3,5-dimethoxyphenyl)pyrene (**6**)[4] and 3,5-dimethoxyphenylboronic acid (**7b**)[5] were synthesized and purified according to reported procedures. All reactions were performed under an argon atmosphere. Column chromatography was performed using Kanto Chemical silica gel 60 N (spherical, neutral). Spectroscopic grade solvents, dichloromethane (DOJINDO) and *n*-hexane (DOJINDO) were used for all spectroscopic studies without further purification.

1.2 Synthesis and characterization

1.2.1 Synthesis of 1c, 2c and 4c

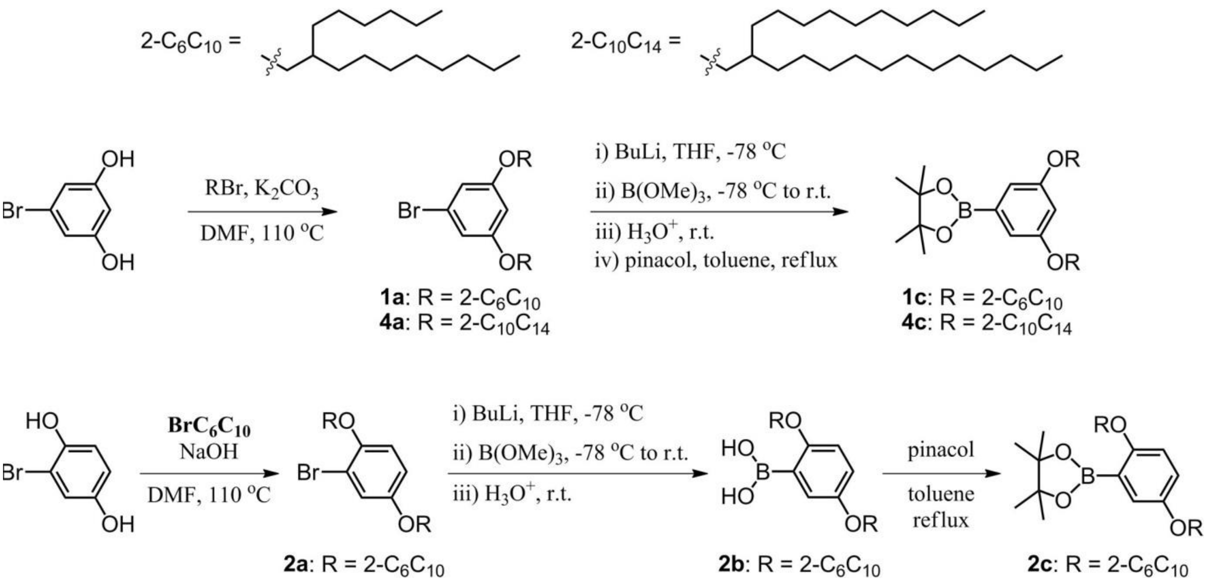

General synthetic procedure for 1a and 4a: A mixture of 5-bromo-1,3-benzenediol (1.90 g, 10.00 mmol, Tokyo Chemical Industry Co., Ltd. (TCI)), corresponding alkyl bromide (**BrC$_6$C$_{10}$** or **BrC$_{10}$C$_{14}$**, 40.00 mmol) and potassium carbonate (K_2CO_3, 11.06 g, 80.00 mmol) was stirred in dry *N*,*N*-dimethylformamide (DMF) (80.0 mL) at 110 °C under argon. After 24 h, the reaction mixture was filtered to remove undissolved solid and the filtrate was concentrated under reduced pressure to remove DMF. Water (50 mL) was added to the resulting residue and then extracted with dichloromethane (3 × 30 mL). The combined organic layer was washed with brine, dried over magnesium sulphate ($MgSO_4$), filtered and evaporated. The crude product was purified by column chromatography (SiO_2; *n*-hexane).

1a: colorless oil (yield, 90%); TLC (SiO_2; *n*-hexane): R_f = 0.69; ^{1}H NMR (400 MHz, $CDCl_3$) δ (ppm): 6.63 (d, J = 2.0 Hz, 2H), 6.36 (t, J = 2.4 Hz, 1H), 3.77 (d, J = 5.6 Hz, 4H), 1.75–1.72 (m, 2H), 1.44–1.27 (m, 48H), 0.88 (t, J = 6.8 Hz, 12H); ^{13}C NMR (100 MHz, $CDCl_3$) δ (ppm): 161.00, 122.78, 110.13, 100.55, 71.08, 37.91, 31.93, 31.87, 31.33, 30.02, 29.69, 29.61, 29.36, 26.82, 22.70, 14.13.
4a: colorless oil (yield, 89%); TLC (SiO_2; *n*-hexane): R_f = 0.79; ^{1}H NMR (400 MHz, $CDCl_3$) δ (ppm): 6.63 (d, J = 2.0 Hz, 2H), 6.36 (t, J = 2.4 Hz, 1H), 3.77 (d, J = 5.6 Hz, 4H), 1.75–1.71 (m, 2H), 1.44–1.22 (m, 80H), 0.88 (t, J = 7.2 Hz, 12H); ^{13}C NMR (100 MHz, $CDCl_3$) δ (ppm): 160.93, 122.68, 110.06, 100.47, 71.01, 37.81, 31.87, 31.24, 29.93, 29.61, 29.57, 29.30, 26.75, 22.64, 14.06.

General synthetic procedure for 1c and 4c: To a solution of **1a** or **4a** (6.00 mmol) in 50 mL dry tetrahydrofuran (THF) at –78 °C under argon was added *n*-butyllithium (*n*-BuLi) (2.54 mL, 6.60 mmol; 2.6 M in *n*-hexane, Kanto Chemical Co., Inc.) dropwise. After stirring at –78 °C for 1.5 h, trimethyl borate ($B(OMe)_3$, 2.41 mL, 21.60 mmol) was injected and the reaction mixture was stirred at –78 °C for 2 h and then at room temperature overnight. The reaction was quenched by hydrochloric acid (HCl) (2 M, a.q.) and extracted with ethyl acetate (3 × 40 mL). The combined organic layer was washed with brine and then dried over $MgSO_4$. After filtration and evaporation, the crude product was purified by column chromatography (SiO_2; *n*-hexane/ethyl acetate, 5:1, v/v) to give a colorless oil with R_f = 0.13 (from reaction of **1a**) or 0.18 (from reaction of **4a**) (TLC: SiO_2; *n*-hexane/ethyl acetate, 5:1, v/v). The oil was refluxed vigorously with pinacol (1.418 g, 12.00 mmol, Sigma-Aldrich) in toluene (60 mL) with Dean-Stark apparatus for 12 h. The resulting mixture was washed with water, brine and then dried over $MgSO_4$. The product was collected via evaporation under reduced pressure without further purification.
1c: yellow green oil (yield, 83%); TLC (SiO_2; *n*-hexane/ethyl acetate, 5:1, v/v): R_f = 0.58; ^{1}H NMR (400 MHz, $CDCl_3$) δ (ppm): 6.92 (d, J = 2.4 Hz, 2H), 6.55 (t, J = 2.4 Hz, 1H), 3.83 (d, J = 5.2 Hz, 4H), 1.73–1.71 (m, 2H), 1.44–1.27 (m, 60H), 0.88 (t, J = 6.4 Hz, 12H); ^{13}C NMR (100 MHz, $CDCl_3$) δ (ppm): 160.21, 112.11, 104.89, 83.79, 70.61, 38.07, 31.92, 31.89, 31.40, 30.04, 29.72, 29.62, 29.36, 26.88, 26.86, 24.84, 22.70, 14.14.
4c: yellow green oil (yield, 40%); TLC (SiO_2; *n*-hexane/ethyl acetate, 5:1, v/v): R_f = 0.73; ^{1}H NMR (400 MHz, $CDCl_3$) δ (ppm): 6.91 (d, J = 2.0 Hz, 2H), 6.55 (t, J = 2.4 Hz, 1H), 3.83 (d, J = 5.2 Hz, 4H), 1.72 (dd, J = 10.8, 5.2 Hz, 2H), 1.44–1.24 (m, 92H), 0.88 (t, J = 6.8 Hz, 12H); ^{13}C NMR (100 MHz, $CDCl_3$) δ (ppm): 160.23, 112.16, 104.94, 83.80, 70.68, 38.10, 31.94, 31.41, 30.05, 29.71, 29.67, 29.38, 26.91, 24.85, 22.71, 14.14.

Synthesis of 2a: A mixture of bromohydroquinone (1.25 g, 6.61 mmol, Wako Pure Chemical Industries, Ltd. (Wako)), $\mathbf{BrC_6C_{10}}$ (4.44 g, 14.54 mmol), sodium hydroxide (NaOH, 661 mg, 16.53 mmol) was stirred in dry DMF (10.0 mL) at 100 °C under argon. After 24 h, the reaction mixture was cooled to room temperature and water (50 mL) was added. The product was extracted with

dichloromethane (3 × 30 mL) and the combined organic layer was washed with brine, dried over $MgSO_4$. The solvent was evaporated and the crude product was purified by column chromatography (SiO_2; *n*-hexane) to give **2a** (2.44 g, 58%) as colorless oil.

2a: colorless oil (yield, 58%); TLC (SiO_2; *n*-hexane): R_f = 0.40; ^{1}H NMR (400 MHz, $CDCl_3$) δ (ppm): 7.11 (d, *J* = 2.8 Hz, 1H), 6.82-6.76 (m, 2H), 3.82 (d, *J* = 5.6 Hz, 2H), 3.75 (d, *J* = 6.0 Hz, 2H), 1.82-1.70 (m, 2H), 1.52-1.27 (m, 48H), 0.88 (t, *J* = 6.4 Hz, 12H); ^{13}C NMR (100 MHz, $CDCl_3$) δ (ppm): 153.74,149.92, 119.51, 114.32, 114.28, 112.72, 72.85, 71.72, 38.04, 37.99, 31.93, 31.87, 31.34, 30.02, 29.70, 29.61, 29.36, 26.83, 22.70, 14.14.

Synthesis of 2b: To a solution of **2a** (2.55 g, 4.00 mmol) in 33 mL dry THF at −78 °C under argon was added *n*-BuLi (1.70 mL, 4.40 mmol; 2.6 M in *n*-hexane) dropwise. After stirring at −78 °C for 1.5 h, $B(OMe)_3$ (0.90 mL, 8.00 mmol) was injected and the reaction mixture was stirred at −78 °C for 2 h and then at room temperature overnight. The reaction was quenched by HCl (2 M, a.q.) and extracted with ethyl acetate (3 × 40 mL). The combined organic layer was washed with brine and then dried over $MgSO_4$. After filtration and evaporation, the crude product was purified by column chromatography (SiO_2; *n*-hexane/ethyl acetate, 50:1, v/v) to give **2b** as yellowish oil (2.16 g, 90%).

2b: yellowish oil (yield, 90%); TLC (SiO_2; *n*-hexane/ethyl acetate, 50:1, v/v): R_f = 0.33; ^{1}H NMR (400 MHz, $CDCl_3$) δ (ppm): 7.36 (d, *J* = 3.6 Hz, 1H), 6.99 (dd, *J* = 8.4, 3.2 Hz, 1H), 6.83 (d, *J* = 8.8 Hz, 1H), 5.90 (s, 2H), 3.91 (d, *J* = 5.6 Hz, 2H), 3.81 (d, *J* = 5.6 Hz, 2H), 1.83–1.72 (m, 2H), 1.46–1.27 (m, 48H), 0.88 (t, *J* = 6.4 Hz, 12H); ^{13}C NMR (100 MHz, $CDCl_3$) δ (ppm): 158.30, 153.51, 121.44, 119.12, 111.84, 71.51, 71.41, 38.09, 38.07, 31.92, 31.88, 31.82, 31.51, 31.48, 31.36, 30.05, 29.94, 29.72, 29.61, 29.56, 29.36, 29.31, 26.83, 22.70, 14.13.

Synthesis of 2c: A mixture of **2b** (2.16 g, 3.59 mmol) and pinacol (945 mg, 8.00 mmol) was refluxed vigorously in toluene (45 mL) with Dean-Stark apparatus for 12 h. The resulting mixture was washed with water, brine and then dried over $MgSO_4$. Evaporation under reduced pressure provided **2c** (2.46 g, quant.) as yellowish oil.

2c: yellowish oil (yield, quant.); TLC (SiO_2; *n*-hexane/ethyl acetate, 10:1, v/v): R_f = 0.52; ^{1}H NMR (400 MHz, $CDCl_3$) δ (ppm): 7.17 (d, *J* = 2.8 Hz, 1H), 6.89 (dd, *J* = 9.2, 3.2 Hz, 1H), 6.75 (d, *J* = 8.8 Hz, 1H), 3.78 (dd, *J* = 6.0, 1.2 Hz, 4H), 1.78–1.69 (m, 2H), 1.55–1.27 (m, 60H), 0.88 (t, *J* = 6.8 Hz, 12H); ^{13}C NMR (100 MHz, $CDCl_3$) δ (ppm): 158.17, 152.91, 121.86, 118.29, 112.77, 83.33, 71.79, 71.40, 38.33, 38.18, 31.95, 31.92, 31.89, 31.40, 31.15, 30.18, 30.05, 29.84, 29.72, 29.68, 29.61, 29.39, 29.36, 26.95, 26.91, 26.87, 24.90, 22.72, 22.69, 14.13.

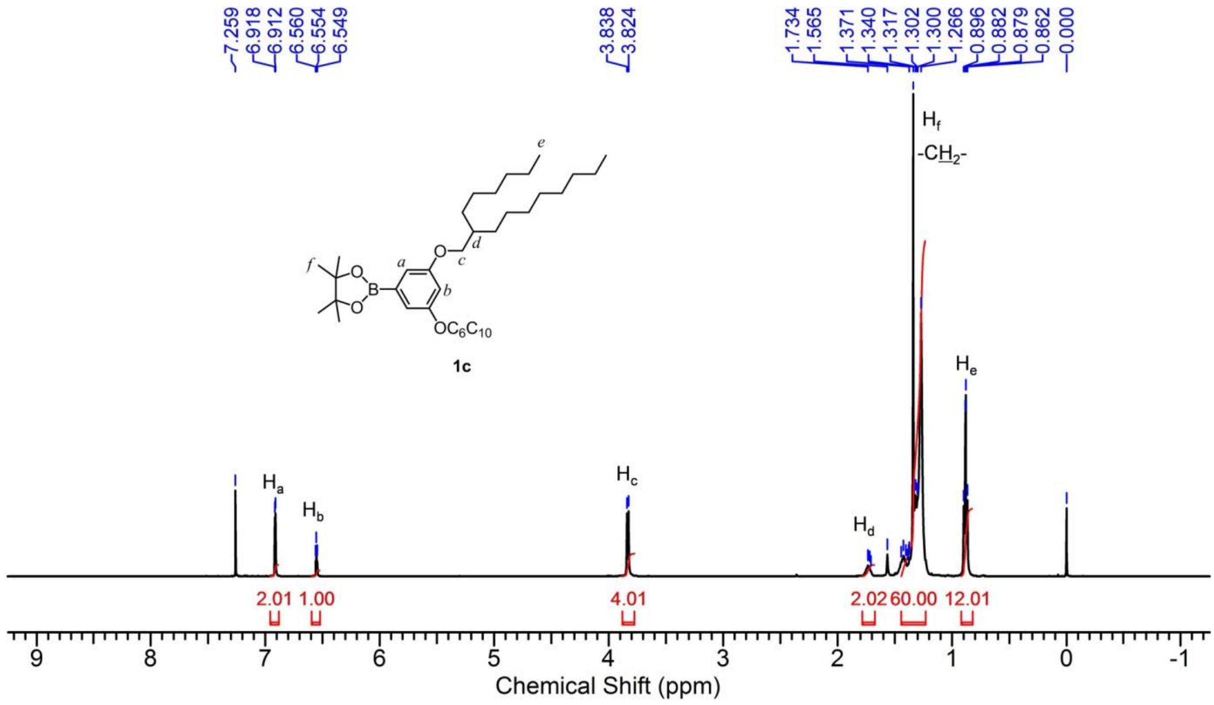

Supplementary Figure 1. ^{1}H NMR (400 MHz, $CDCl_3$) spectrum of **1c**.

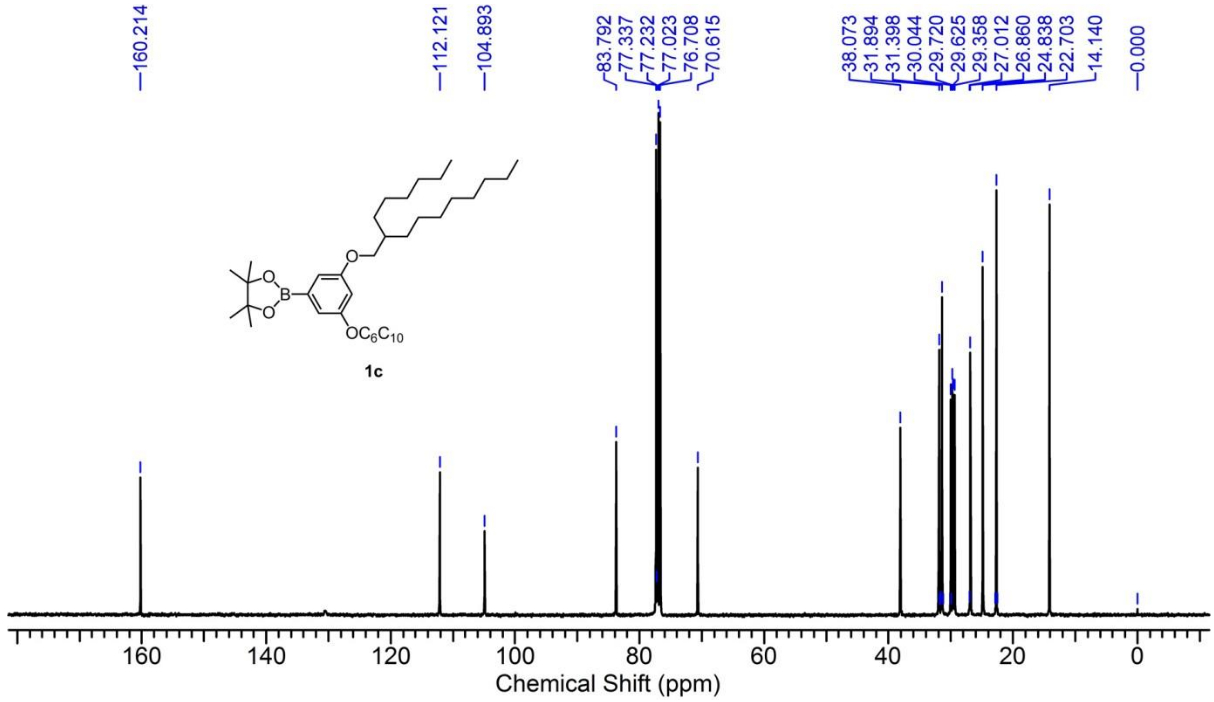

Supplementary Figure 2. ^{13}C NMR (100 MHz, $CDCl_3$) spectrum of **1c**.

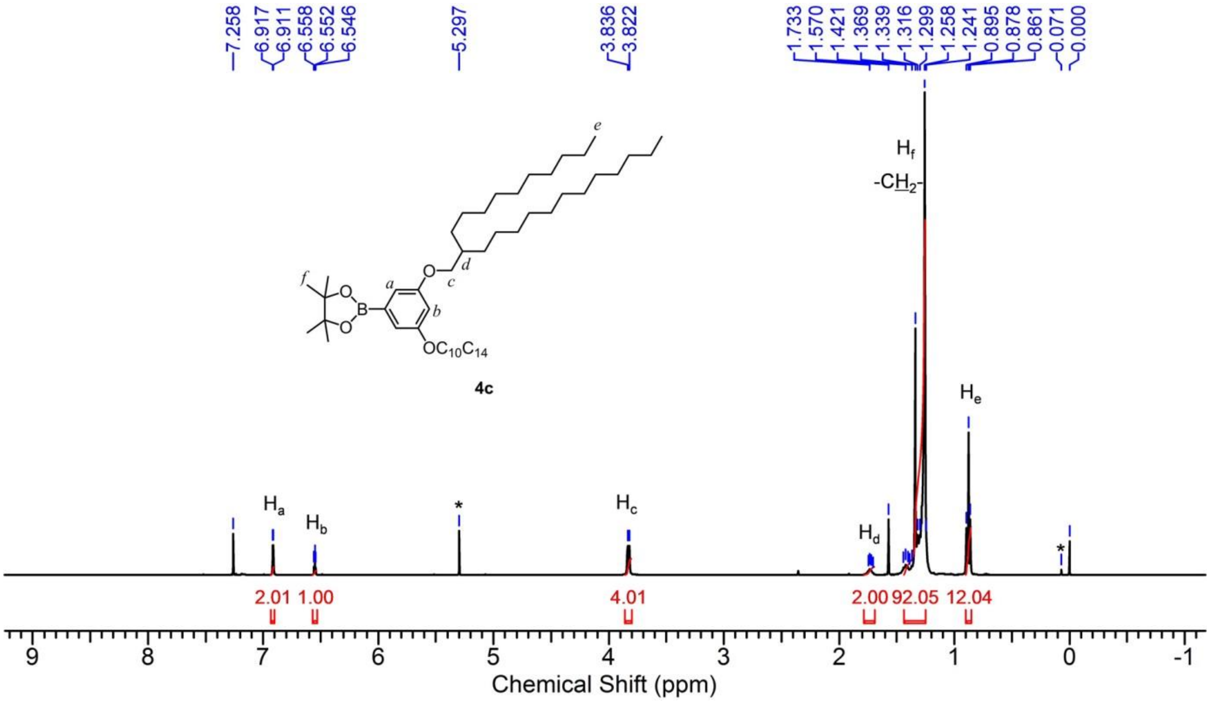

Supplementary Figure 3. ^{1}H NMR (400 MHz, $CDCl_3$) spectrum of **4c**. Asterisks denote residual solvent and impurity (5.30 ppm: dichloromethane, 0.07 ppm: grease) originated from purification procedures.

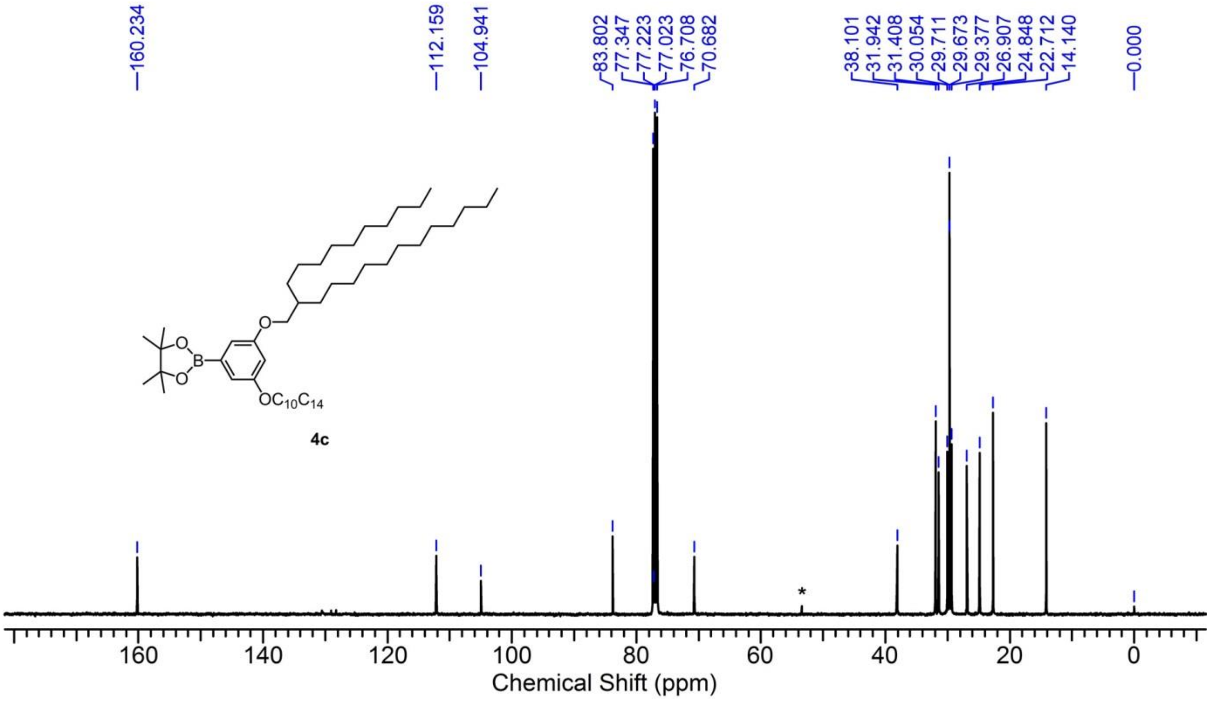

Supplementary Figure 4. ^{13}C NMR (100 MHz, $CDCl_3$) spectrum of **4c**. An asterisk denotes residual solvent (53.52 ppm: dichloromethane) originated from purification procedures.

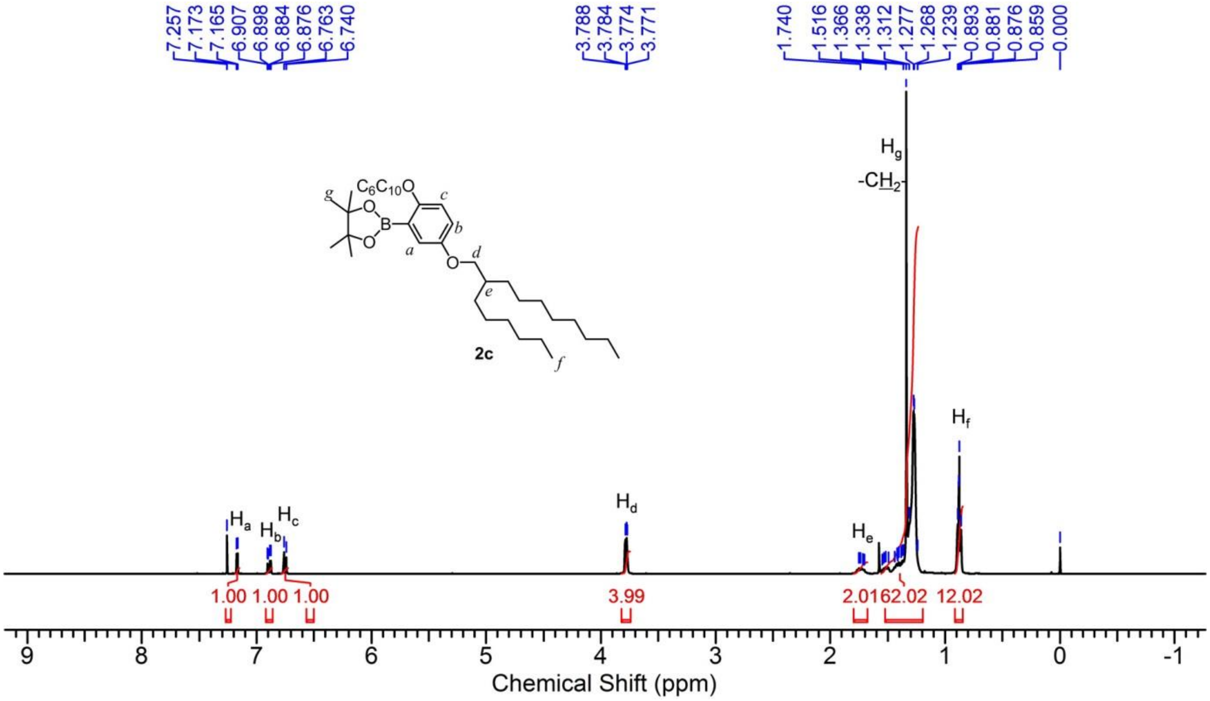

Supplementary Figure 5. ^{1}H NMR (400 MHz, $CDCl_3$) spectrum of **2c**.

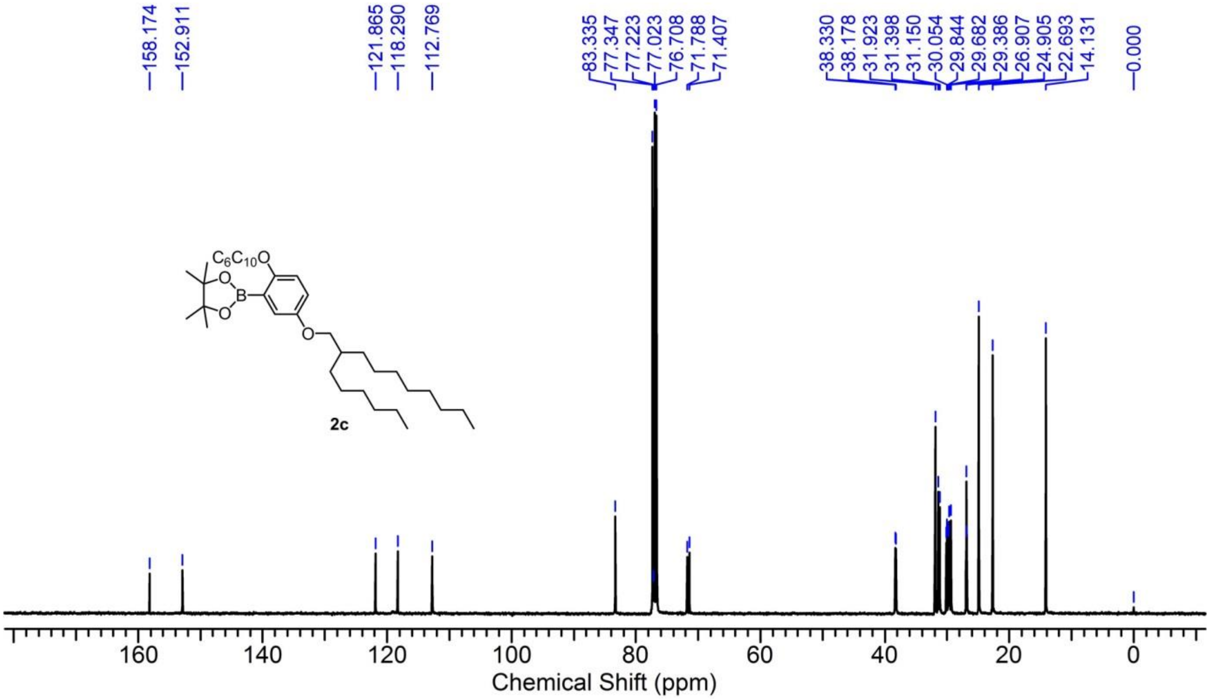

Supplementary Figure 6. ^{13}C NMR (100 MHz, $CDCl_3$) spectrum of **2c**.

1.2.2 Synthesis of 1 and 2

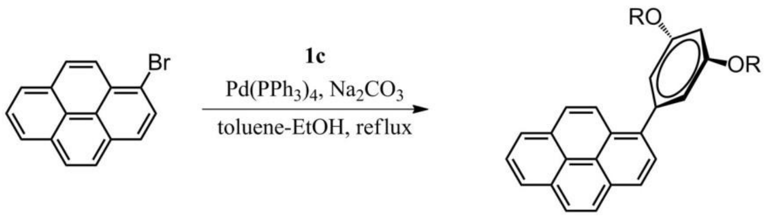

1: R = 2-C_6C_{10}

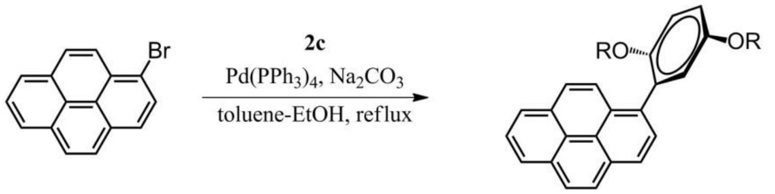

2: R = 2-C_6C_{10}

General synthetic procedure for 1 and 2: A mixture of 1-bromopyrene (384 mg, 1.36 mmol, Wako), corresponding boronic acid pinacol ester **1c** or **2c** (1.50 mmol), sodium carbonate (Na_2CO_3, 1.4 mL, 2 M a.q.), tetrakis(triphenylphosphine)palladium(0) ($Pd(PPh_3)_4$, 79 mg, 0.068 mmol, TCI) was refluxed in toluene-ethanol (54 mL-5.4 mL) under argon for 24–48 h until all starting material was consumed by TLC analysis. The reaction mixture was cooled, filtered and then evaporated to remove toluene. After evaporation, water (30 mL) was added and the aqueous layer was extracted with dichloromethane (3 × 30 mL). The combined organic layer was washed with brine (70 mL), dried over $MgSO_4$ and evaporated. The crude product was purified via column chromatography (SiO_2; dichloromethane/*n*-hexane, 1:5 v/v) and subsequent recycling HPLC with chloroform as the solvent to give the pure product.

1: pale green liquid (yield, 97%), T_g: –45.5 °C; TLC (SiO_2; dichloromethane/*n*-hexane, 1:20 v/v): R_f = 0.35; ^{1}H NMR (400 MHz, $CDCl_3$) δ (ppm): 8.26 (dd, J = 12.0, 2.8 Hz, 1H), 8.21–8.15 (m, 3H), 8.09 (s, 2H), 8.04–7.99 (m, 3H), 6.75 (t, J = 2.0 Hz, 2H), 3.89 (d, J = 3.2 Hz, 4H), 1.83–1.77 (m, 2H), 1.46–1.27 (m, 48H), 0.86 (t, J = 2.8 Hz, 12H); ^{13}C NMR (100 MHz, $CDCl_3$) δ (ppm): 160.41, 142.99, 137.97, 131.48, 130.99, 130.56, 128.47, 127.42, 127.37, 127.31, 125.98, 125.52, 125.05, 124.89, 124.80, 124.50, 109.18, 100.45, 71.13, 38.04, 31.92, 31.88, 31.42, 30.04, 29.72, 29.61, 29.35, 26.88, 26.87, 22.68, 14.12; MALDI-TOF MS (matrix: dithranol) calculated for $C_{54}H_{78}O_2$: 758.6, found: 758.3 $[M]^+$.

2: yellowish liquid (yield, 97%), T_g: –43.0 °C; TLC (SiO_2; dichloromethane/*n*-hexane, 1:5 v/v): R_f = 0.24; ^{1}H NMR (400 MHz, $CDCl_3$) δ (ppm): 8.20–8.06 (m, 5H), 8.00–7.91 (m, 4H), 7.04–6.97 (m, 3H), 3.84 (d, J = 5.6 Hz, 2H), 3.68–3.59 (m, 2H), 1.79–1.75 (m, 1H), 1.49–0.72 (m, 61H); ^{13}C NMR (100 MHz, $CDCl_3$) δ (ppm): 153.48, 151.22, 134.56, 131.71, 131.40, 131.04, 130.52, 129.26, 128.04, 127.44, 126.89, 126.26, 125.74, 124.83, 124.73, 124.64, 124.24, 118.54, 114.61, 72.63, 71.73, 38.11, 37.90, 31.89, 31.87, 31.83, 31.63, 31.41, 31.09, 30.04, 29.71, 29.61, 29.39, 29.34, 29.22, 26.85, 26.61,

26.58, 26.54, 22.67, 22.53, 14.11, 14.02; MALDI-TOF MS (matrix: dithranol) calculated for $C_{54}H_{78}O_2$: 758.6, found: 758.5 $[M]^+$.

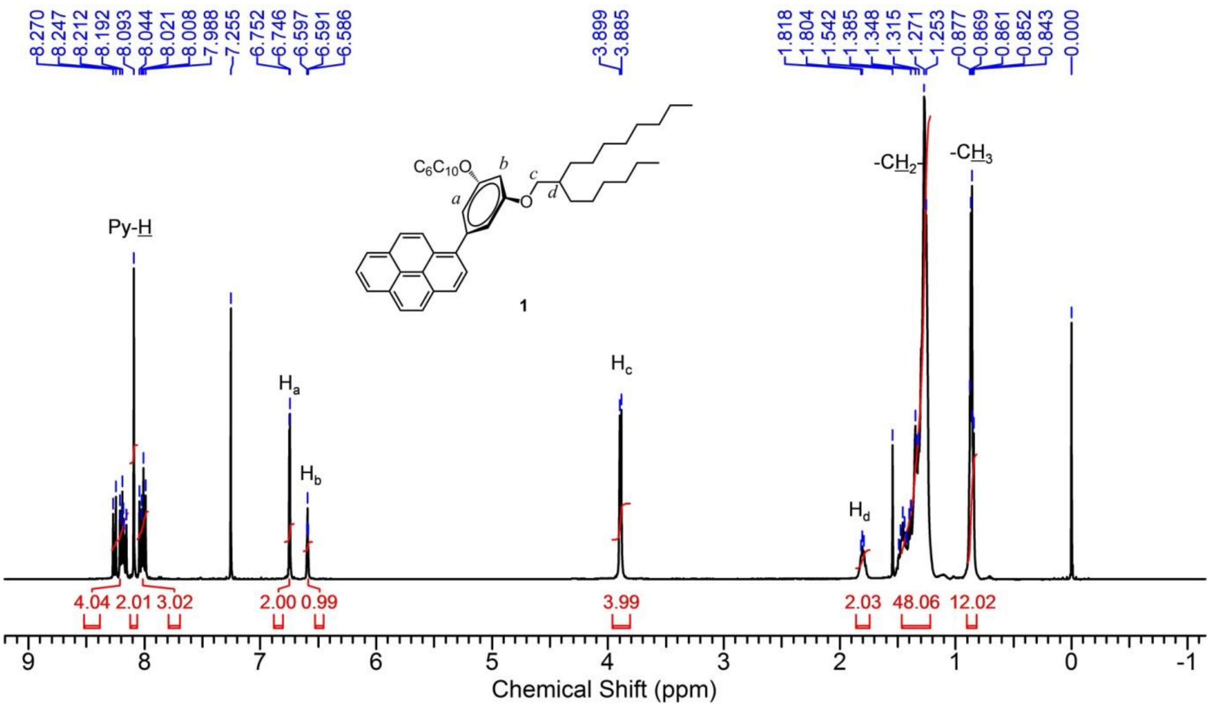

Supplementary Figure 7. ^{1}H NMR (400 MHz, $CDCl_3$) spectrum of **1**.

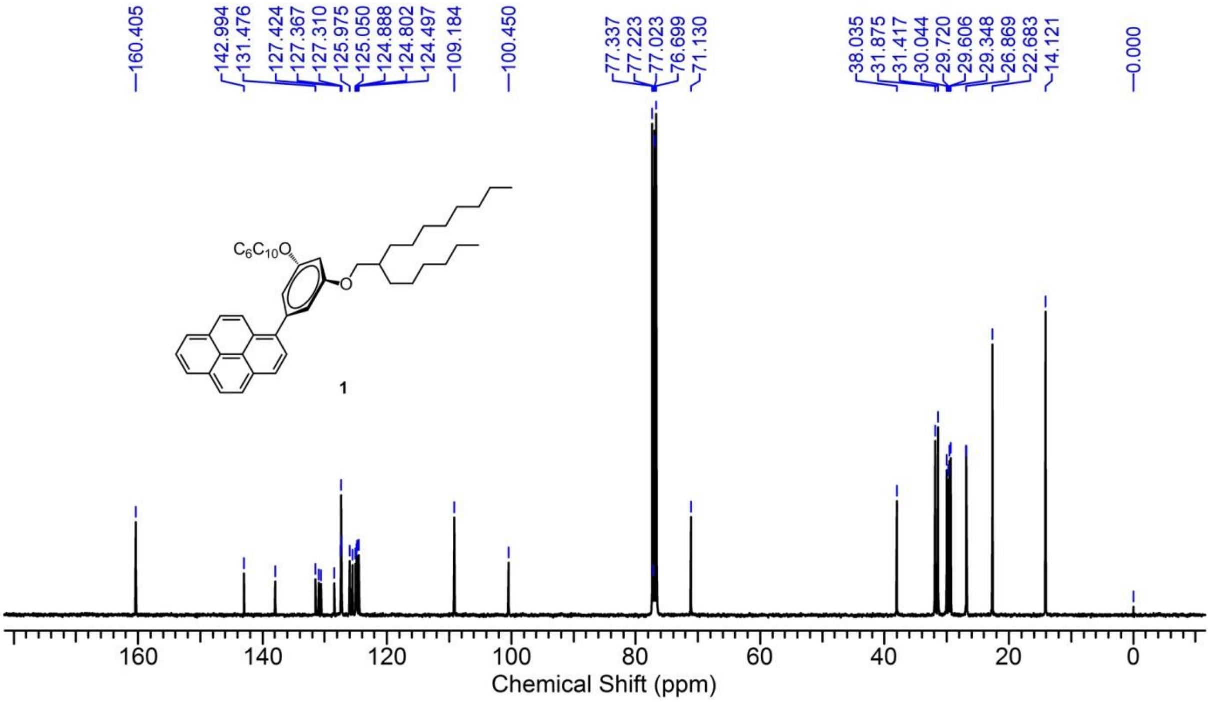

Supplementary Figure 8. ^{13}C NMR (100 MHz, $CDCl_3$) spectrum of **1**.

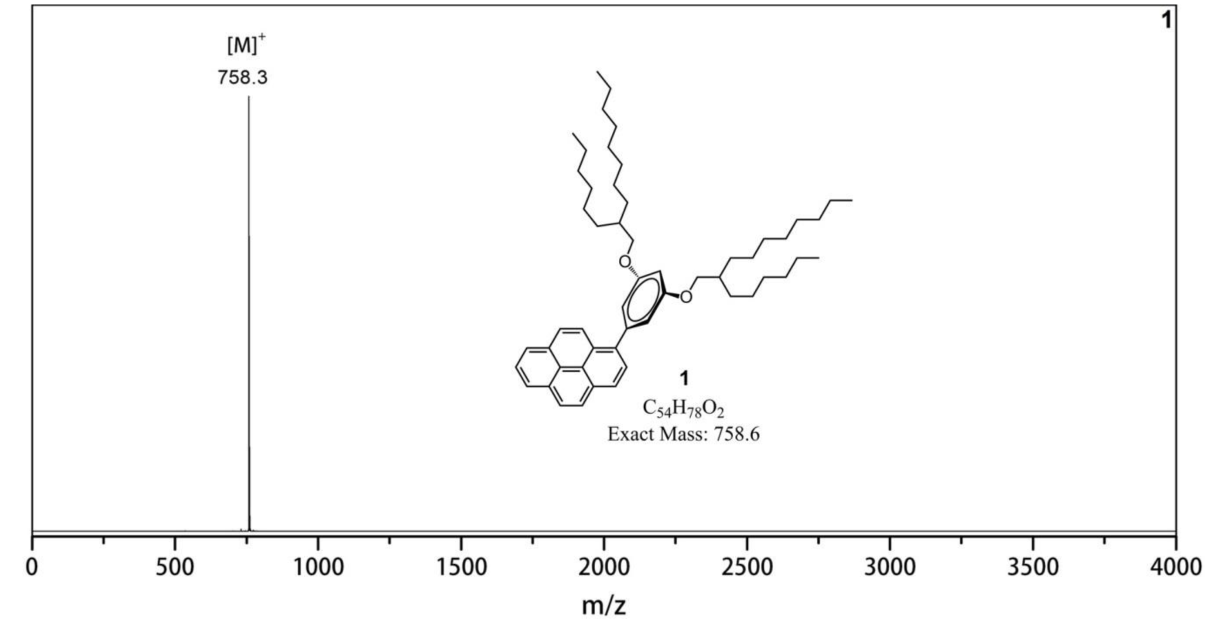

Supplementary Figure 9. MALDI-TOF MS of **1**. Matrix: dithranol.

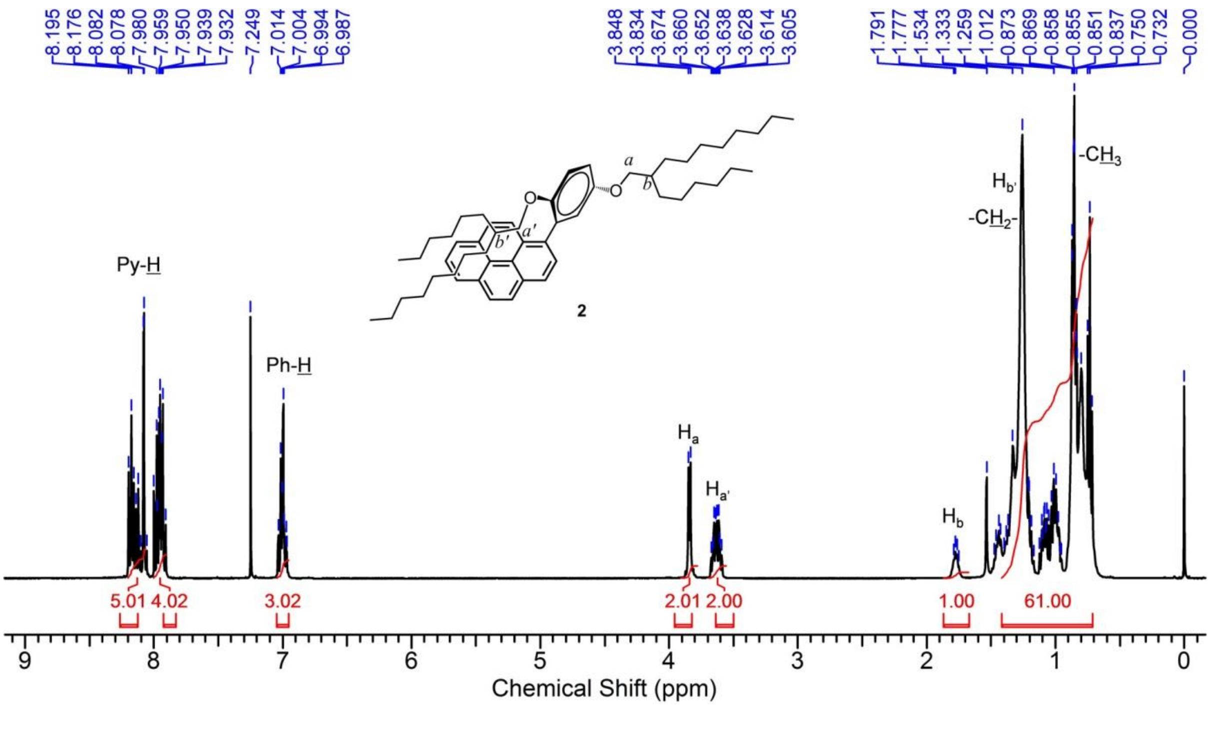

Supplementary Figure 10. ^{1}H NMR (400 MHz, $CDCl_3$) spectrum of **2**.

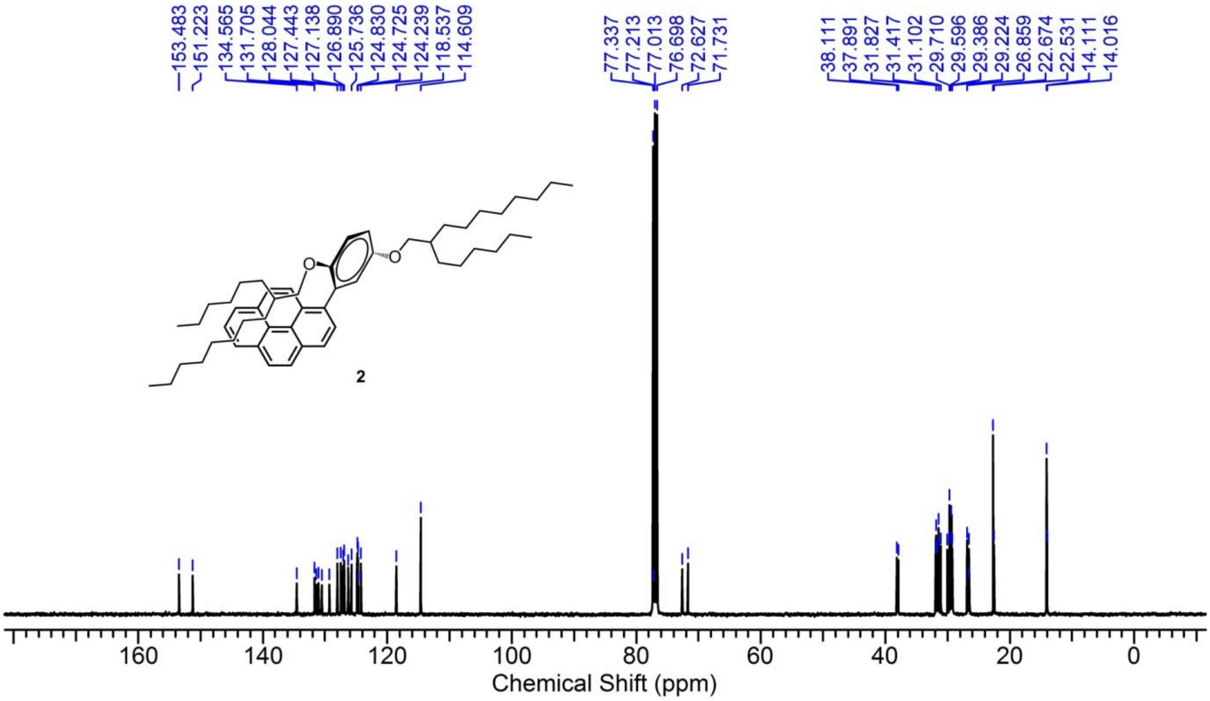

Supplementary Figure 11. ^{13}C NMR (100 MHz, $CDCl_3$) spectrum of **2**.

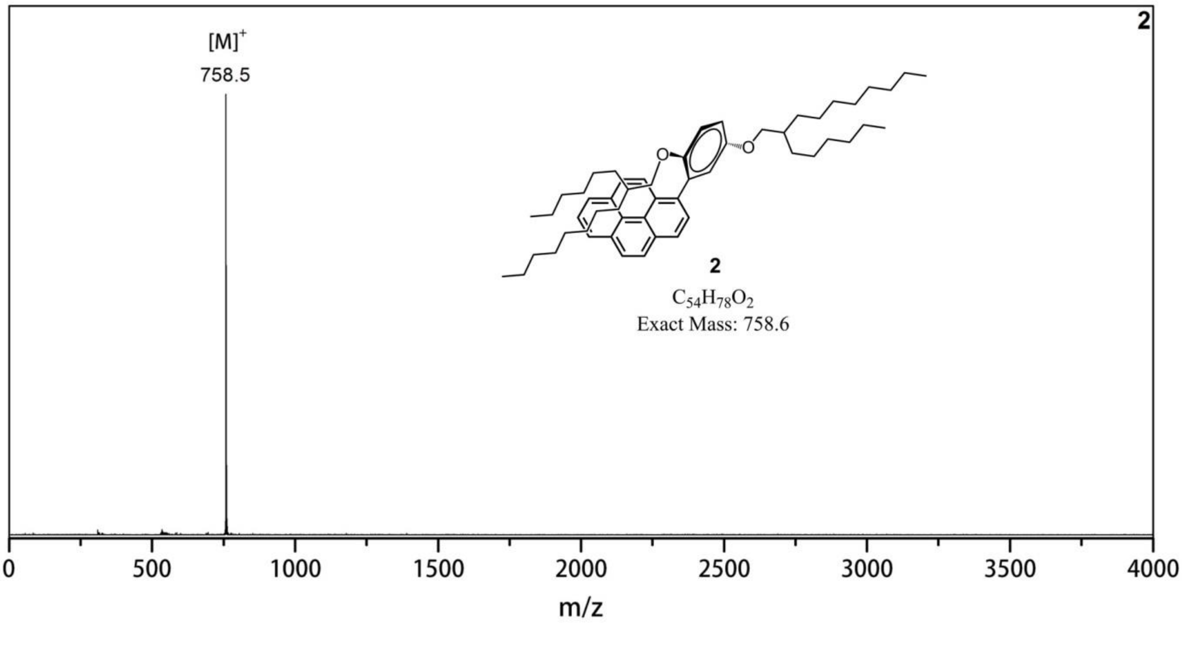

Supplementary Figure 12. MALDI-TOF MS of **2**. Matrix: dithranol.

1.2.3 Synthesis of 3, 4, 5 and 7

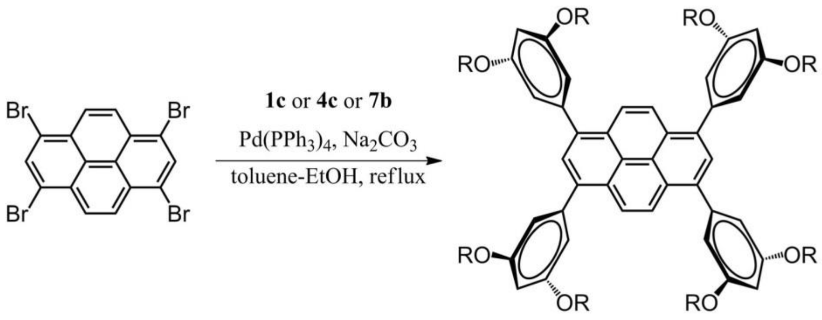

3: R = 2-C_6C_{10}
4: R = 2-$C_{10}C_{14}$
7: R = CH_3

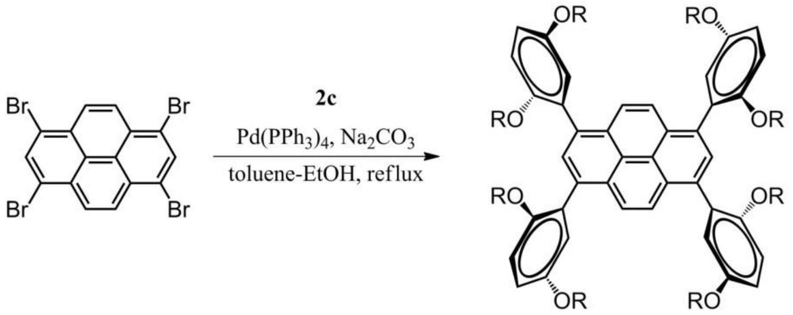

5: R = 2-C_6C_{10}

General synthetic procedure for 3, 4, 5, 7: A mixture of 1,3,6,8-tetrabromopyrene (570 mg, 1.10 mmol, TCI), corresponding boronic acid pinacol ester **1c** or **4c** or **2c** or 3,5-dimethoxyphenylboronic acid (**6b**) (6.00 mmol), Na_2CO_3 (4.4 mL, 2 M a.q.), $Pd(PPh_3)_4$ (254 mg, 0.22 mmol) was refluxed in toluene-ethanol (45 mL-4.5 mL) under argon for 48 h until all starting material was consumed by TLC analysis. The reaction mixture was cooled, filtered and then evaporated to remove toluene. After evaporation, water (30 mL) was added and the aqueous layer was extracted with dichloromethane (3 × 30 mL). The combined organic layer was washed with brine (70 mL), dried over $MgSO_4$ and evaporated. The crude product was purified via column chromatography (SiO_2; dichloromethane/*n*-hexane, 1:5 v/v) and subsequent recycling HPLC with chloroform as the solvent.

3: yellow-green liquid (yield, 90%), T_g: –63.5 °C; TLC (SiO_2; dichloromethane/*n*-hexane, 1:20 v/v): R_f = 0.40; ^{1}H NMR (400 MHz, $CDCl_3$) δ (ppm): 8.24 (s, 4H), 8.02 (s, 2H), 6.77 (d, J = 2.0 Hz, 8H), 6.57 (t, J = 2.4 Hz, 4H), 3.87 (d, J = 5.6 Hz, 16H), 1.80–1.75 (m, 8H), 1.48–1.24 (m, 192H), 0.85 (t, J = 6.4 Hz, 48H); ^{13}C NMR (100 MHz, $CDCl_3$) δ (ppm): 160.43, 142.93, 137.25, 128.91, 128.01, 125.75, 125.36, 109.18, 100.38, 71.07, 37.06, 31.88, 31.86, 31.40, 30.02, 29.69, 29.59, 29.33, 26.88, 26.85, 22.66, 14.10; MALDI-TOF MS (matrix: dithranol) calculated for $C_{168}H_{282}O_8$: 2428.2, found: 2428.5 $[M]^+$.

4: yellow-green liquid (yield, 56%), m.p. –51.9 °C; TLC (SiO_2; *n*-hexane): R_f = 0.43; ^{1}H NMR (400 MHz, $CDCl_3$) δ (ppm): 8.23 (s, 4H), 8.01 (s, 2H), 6.76 (d, J = 2.4 Hz, 8H), 6.55 (t, J = 2.4 Hz, 4H), 3.86 (d, J = 5.2 Hz, 16H), 1.79–1.75 (m, 8H), 1.47–1.22 (m, 320H), 0.85 (td, J = 6.4, 2.4 Hz, 48H); ^{13}C NMR (100 MHz, $CDCl_3$) δ (ppm): 160.41, 142.92, 137.21, 128.91, 128.01, 125.77, 125.36,

109.21, 100.39, 71.11, 38.08, 31.92, 31.39, 30.04, 29.70, 29.65, 29.36, 26.90, 22.69, 14.11; MALDI-TOF MS (matrix: 2-(4'-hydroxybenzene-azo)benzoic acid) calculated for $C_{232}H_{410}O_8$: 3325.2, found: 3326.8 $[M+H]^+$.

5: greenish liquid (yield, 82%), T_g: –57.6 °C; TLC (SiO_2; dichloromethane/*n*-hexane, 1:5 v/v): R_f = 0.42; ^{1}H NMR (400 MHz, $CDCl_3$) δ (ppm): 7.92–7.83 (m, 6H), 7.00-6.89 (m, 12H), 3.78 (d, J = 4.4 Hz, 8H), 3.68–3.53 (m, 8H) , 1.77–1.71 (m, 4H), 1.44–0.64 (m, 244H); ^{13}C NMR (100 MHz, $CDCl_3$) δ (ppm): 153.46, 153.38, 153.27, 153.15, 153.04, 151.29, 151.24, 133.29, 133.05, 131.92, 131.72, 131.53, 131.39, 130.50, 130.30, 129.96, 128.68, 128.60, 125.53, 125.37, 125.28, 125.09, 119.34, 119.09, 118.85, 118.70, 115.38, 114.37, 114.10, 72.64, 72.70, 71.88, 71.64, 71.52, 38.27, 38.22, 38.06, 37.92, 37.78, 37.63, 31.92, 31.80, 31.74, 31.60, 31.42, 31.06, 31.00, 30.09, 29.75, 29.63, 29.53, 29.38, 29.23, 29.18, 29.12, 26.89, 26.63, 26.58, 26.51, 26.39, 26.24, 22.69, 22.52, 22.47, 14.11; MALDI-TOF MS (matrix: dithranol) calculated for $C_{168}H_{282}O_8$: 2428.2, found: 2428.7 $[M]^+$.

7: yellow powder (yield, 82%), m.p. 316–319 °C; TLC (SiO_2; dichloromethane/*n*-hexane, 1:2 v/v): R_f = 0.32; ^{1}H NMR (400 MHz, $CDCl_3$) δ (ppm): 8.22 (s, 4H), 8.02 (s, 2H), 6.81 (d, J = 2.4 Hz, 8H), 6.59 (t, J = 2.0 Hz, 4H) , 3.87 (s, 24H); ^{13}C NMR (100 MHz, $CDCl_3$) δ (ppm): 160.67, 143.02, 137.14, 128.84, 128.15, 125.76, 125.40, 108.88, 99.56, 55.53; MALDI-TOF MS (matrix: dithranol) calculated for $C_{48}H_{42}O_8$: 746.3, found: 747.6 $[M+H]^+$.

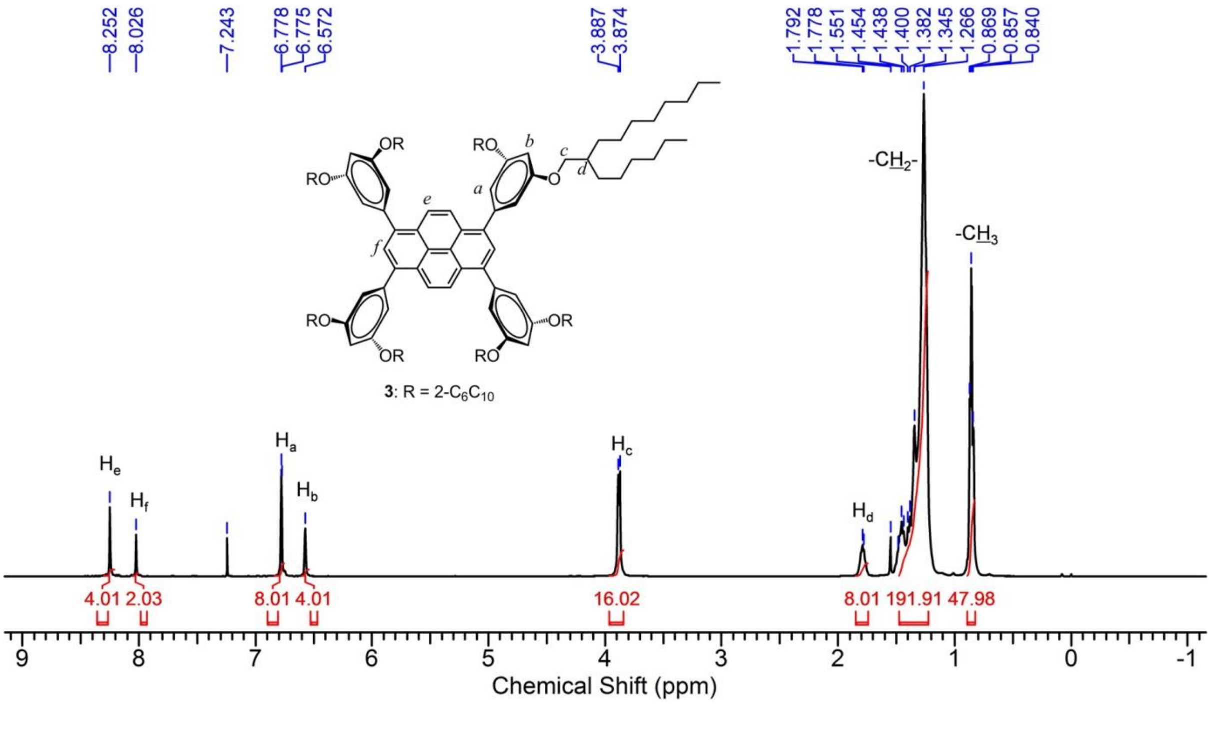

Supplementary Figure 13. ^{1}H NMR (400 MHz, $CDCl_3$) spectrum of **3**.

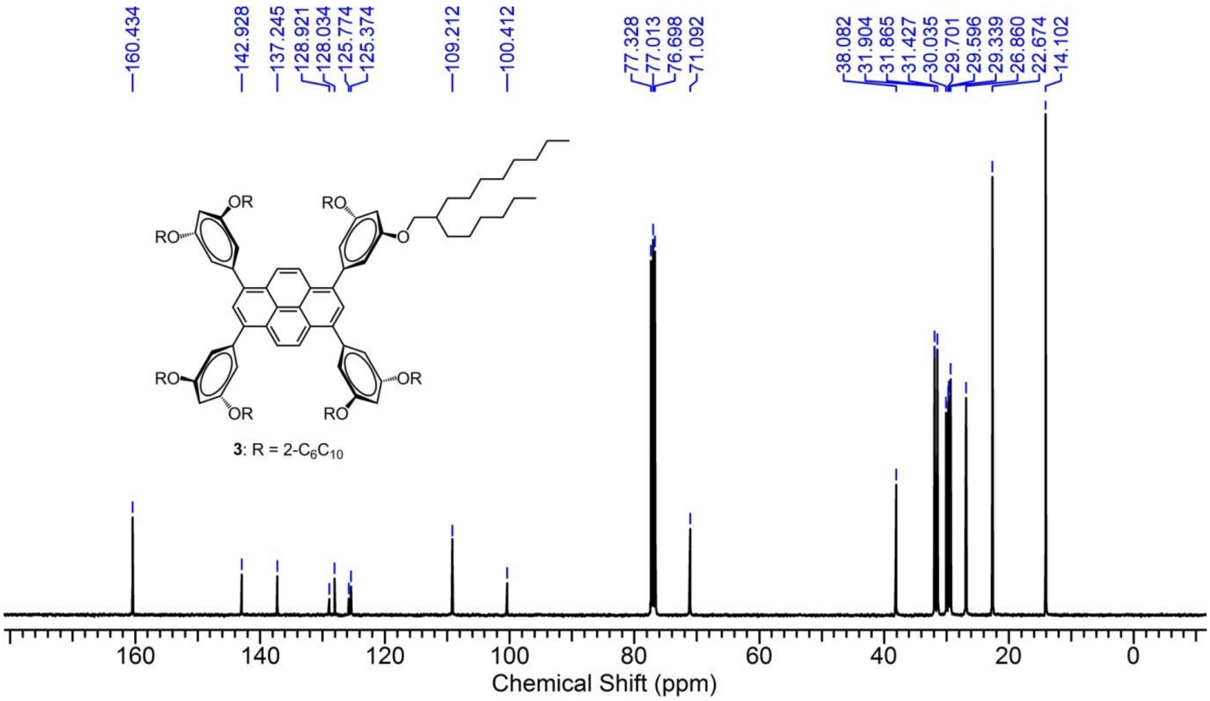

Supplementary Figure 14. ^{13}C NMR (100 MHz, $CDCl_3$) spectrum of **3**.

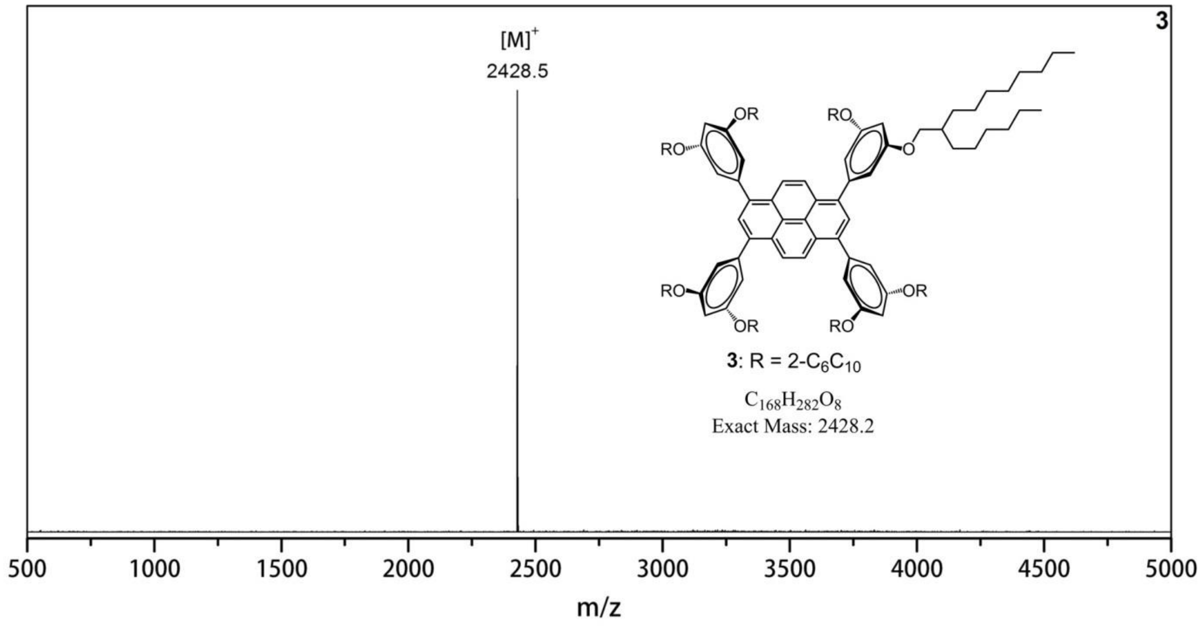

Supplementary Figure 15. MALDI-TOF MS of **3**. Matrix: dithranol.

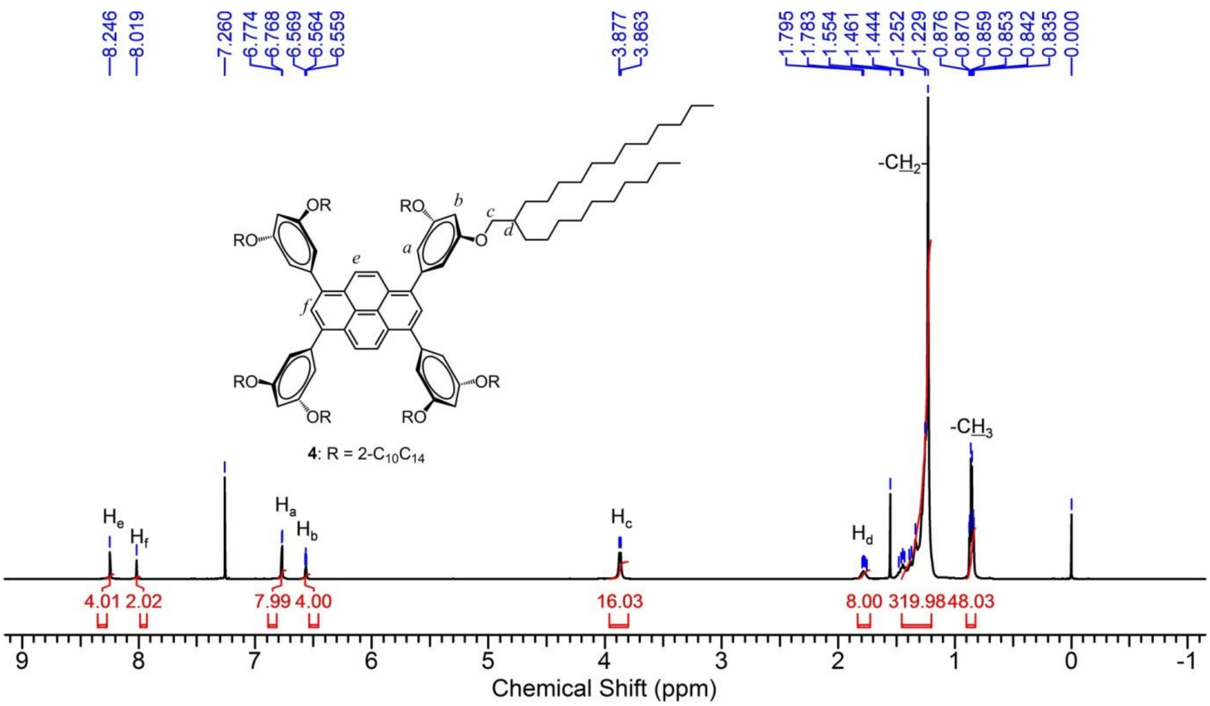

Supplementary Figure 16. ^{1}H NMR (400 MHz, $CDCl_3$) spectrum of **4**.

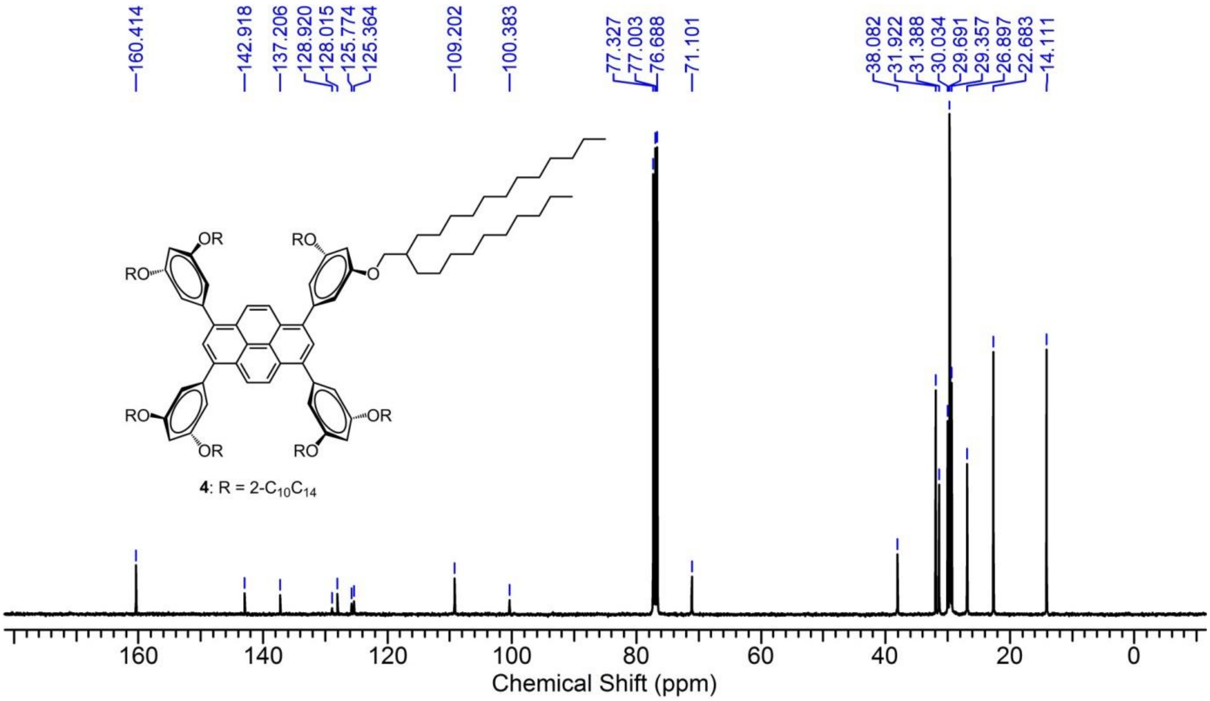

Supplementary Figure 17. ^{13}C NMR (100 MHz, $CDCl_3$) spectrum of **4**.

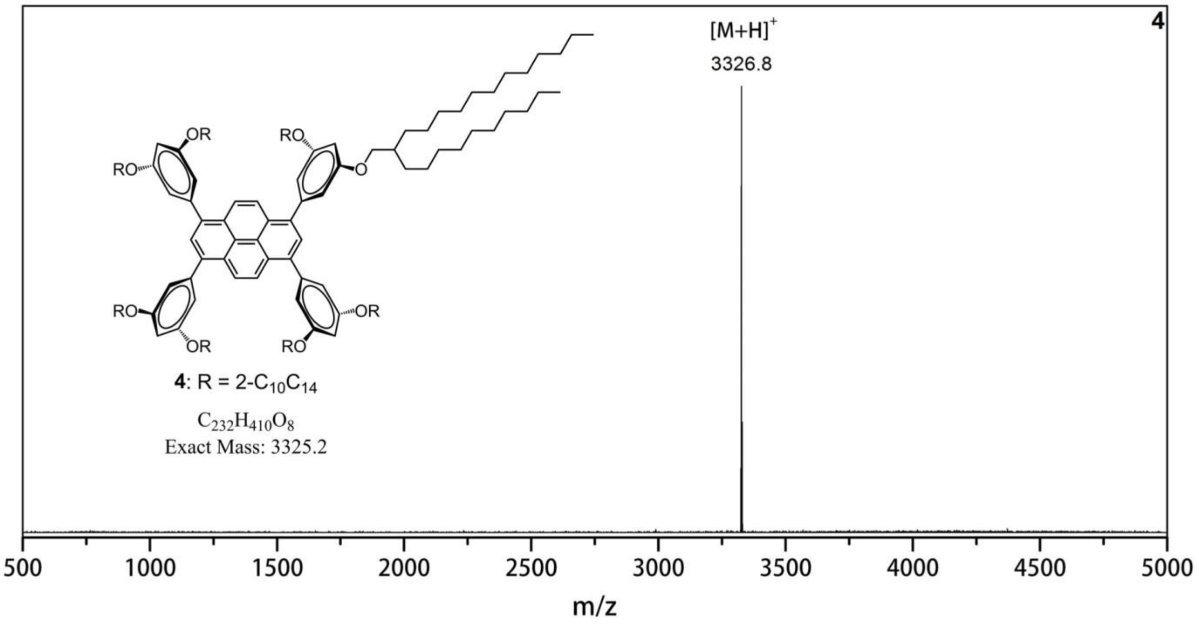

Supplementary Figure 18. MALDI-TOF MS of **4**. Matrix: 2-(4'-hydroxybenzene-azo)benzoic acid.

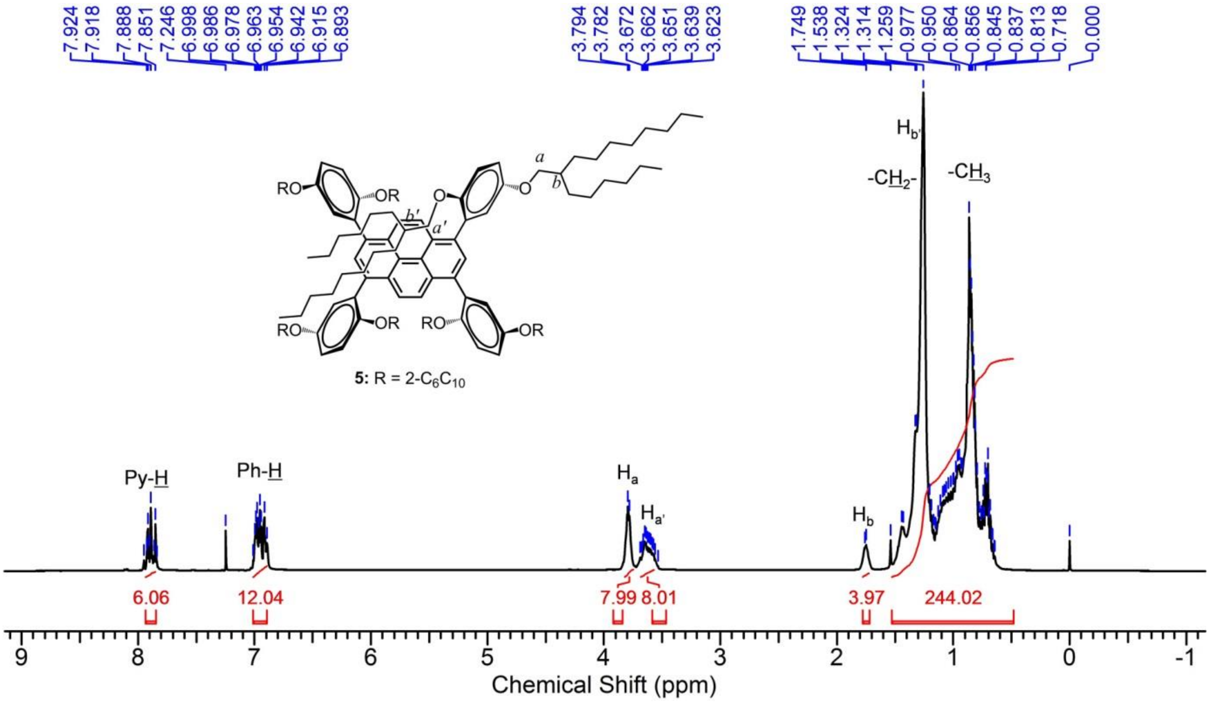

Supplementary Figure 19. ^{1}H NMR (400 MHz, $CDCl_3$) spectrum of **5**.

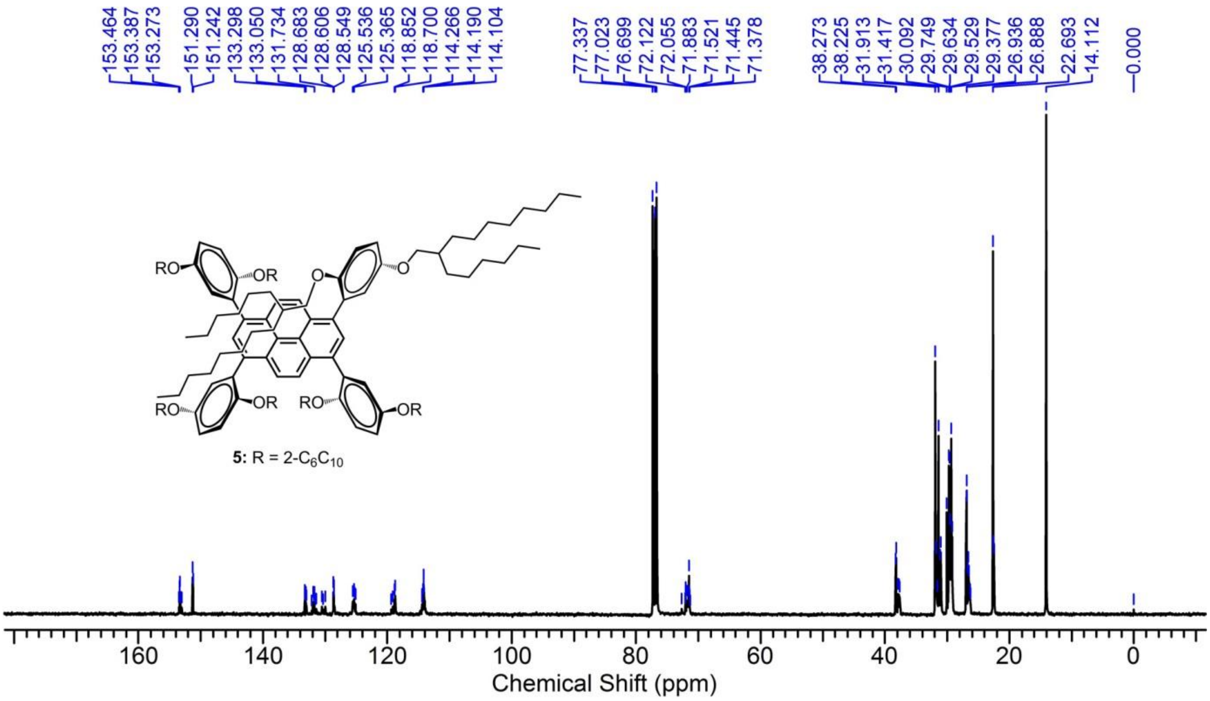

Supplementary Figure 20. ^{13}C NMR (100 MHz, $CDCl_3$) spectrum of **5**.

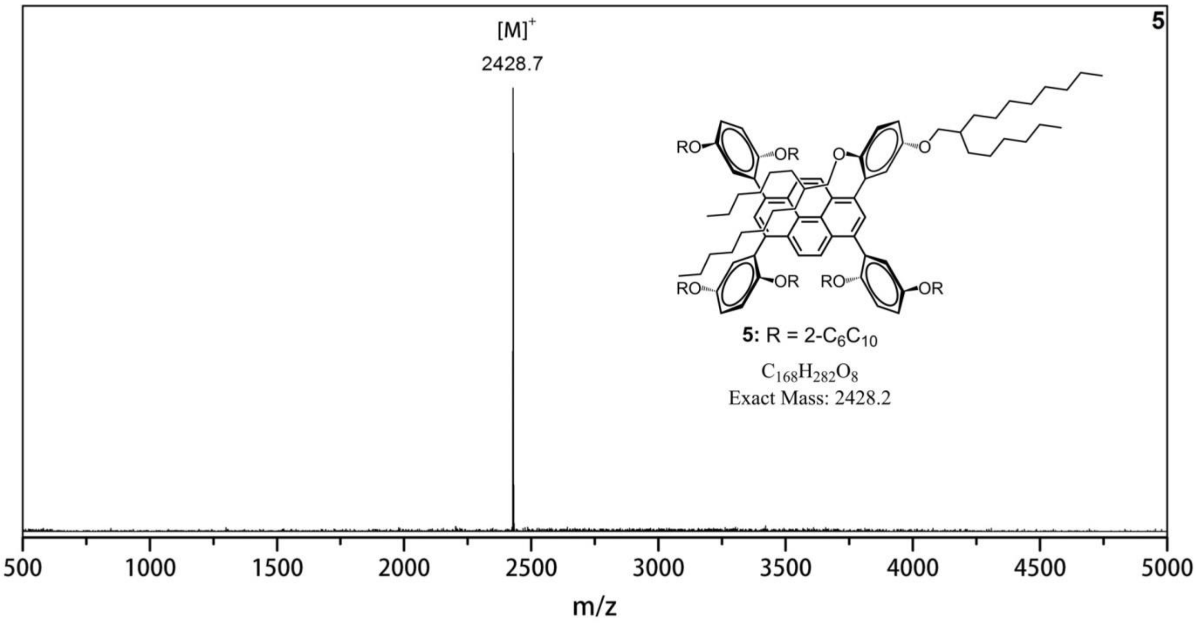

Supplementary Figure 21. MALDI-TOF MS of **5**. Matrix: dithranol.

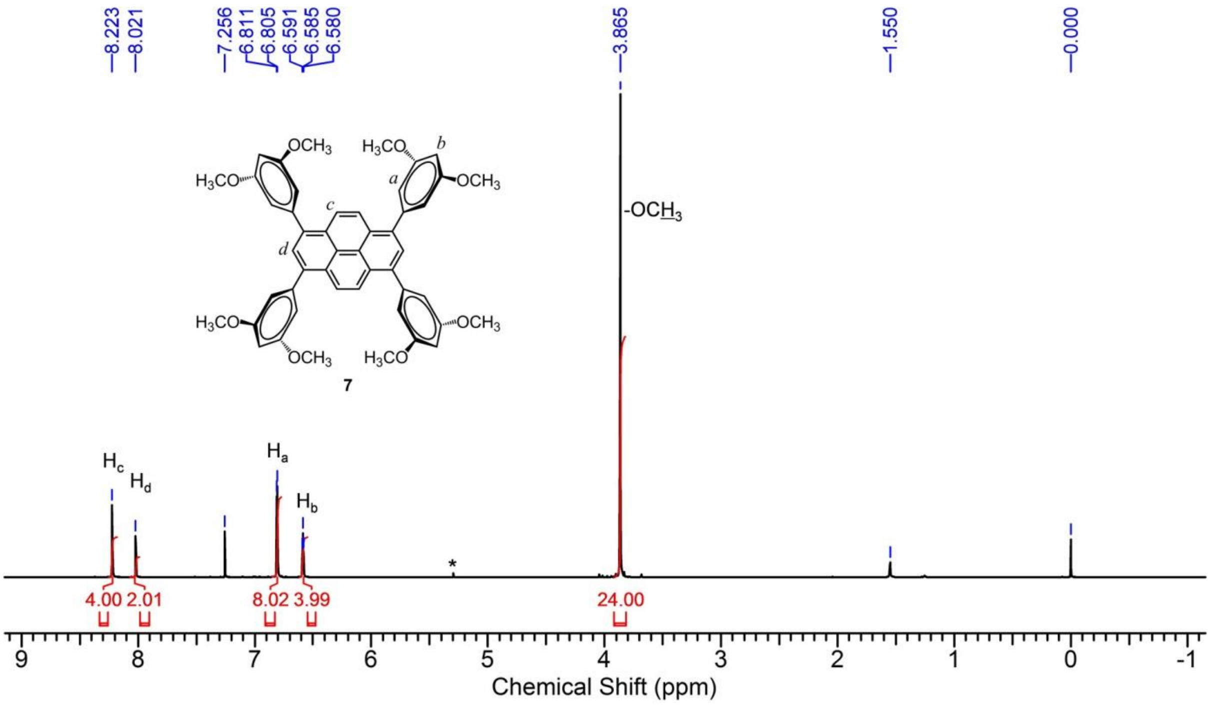

Supplementary Figure 22. ^{1}H NMR (400 MHz, $CDCl_3$) spectrum of **7**. An asterisk denotes residual solvent (53.52 ppm: dichloromethane) originated from purification procedures.

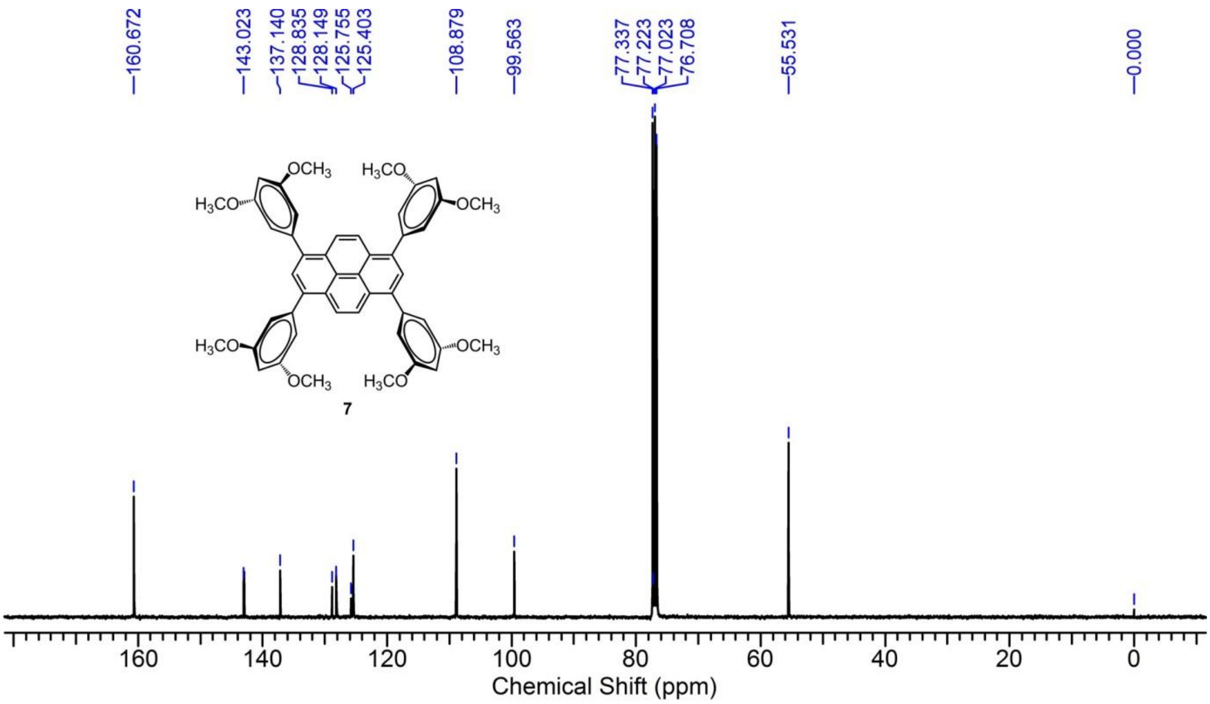

Supplementary Figure 23. ^{13}C NMR (100 MHz, $CDCl_3$) spectrum of **7**.

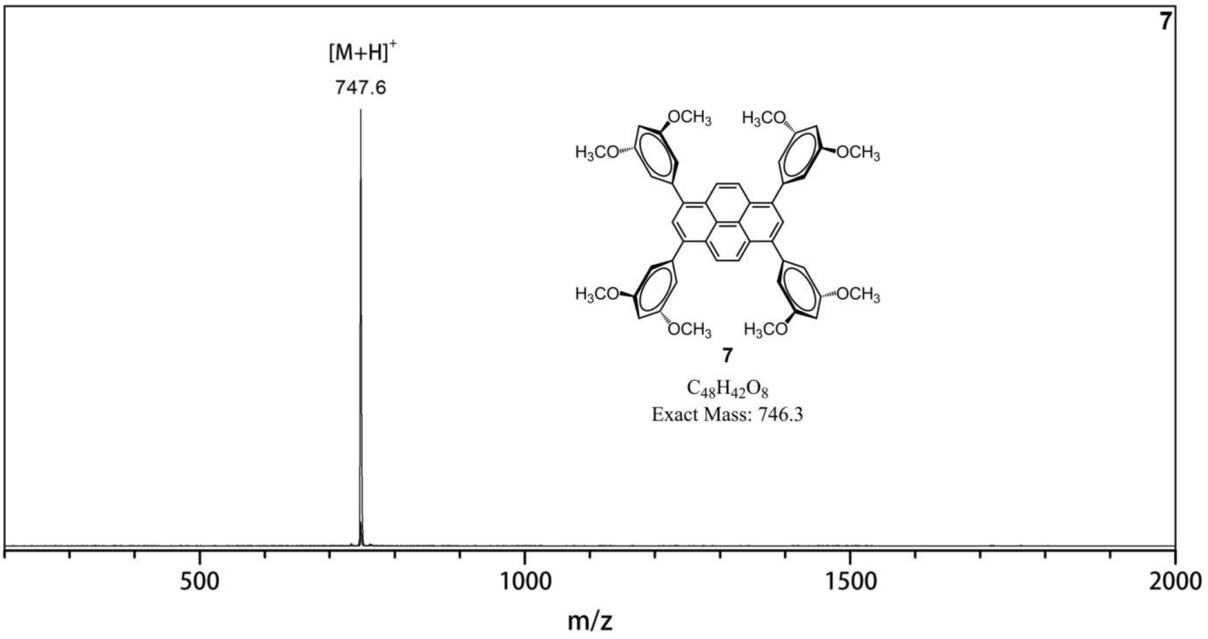

Supplementary Figure 24. MALDI-TOF MS of **7**. Matrix: dithranol.

1.2.4 NMR discussion

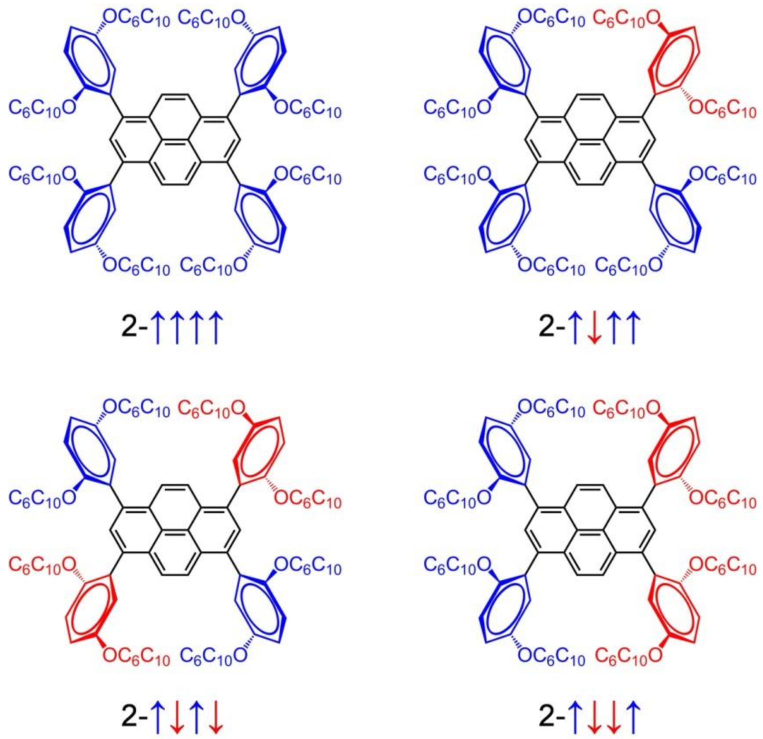

Supplementary Figure 25. Possible conformational isomers of **5** indicated by the different orientations of the 2-substitution alkyl chains on the phenyl unit. Arrows denote orientation of the four alkyl chains substituted at 2-position on phenyl unit relative to pyrene surface (blue, up; red, down). In contrast to the well-resolved and sharp NMR signals of **1**–**4** (Supplementary Figs 7, 8, 10, 11, 13, 14, 16, 17), pyrene **5** with four asymmetric (2,5)-alkyl chain substituted phenyl units exhibited complex and broad signals in both ^{1}H (Supplementary Fig. 19) and ^{13}C (Supplementary Fig. 20) NMR spectra which were probably due to the coexistence of several conformational isomers. These isomer mixtures originated from the rotation barrier of the single bond between pyrene core and the phenyl unit with steric *ortho* substitution. In spite of the complicated NMR spectra, MALDI-TOF MS of **5** clearly showed a single molecular ion peak which was in good agreement with the exact molecular weight (Supplementary Fig. 21), eliminating the existence of impurities. It should be noted that pyrene **2** appended with one phenyl unit, with similar (2,5)-alkyl chain substitution motif to **5** though, possessed only one conformational structure, which accounted for its well-defined and sharp NMR signals (Supplementary Figs 10 and 11).

1.3 Techniques

Recycling high performance liquid chromatography (HPLC) was performed at room temperature using a GPC column (YMC-GPC T30000 ϕ20 × 600 mm) on a JAI LC-9225NEXT system, equipped with RI and UV-Vis detectors. Melting points were determined on a Yanaco melting point apparatus MP-500P. ^{1}H NMR and ^{13}C NMR spectra were recorded on a JEOL ECS-400 spectrometer at 400 MHz and 100 MHz, respectively, with tetramethylsilane as the internal standard. Before ^{1}H and ^{13}C NMR measurements, all samples were dried up from their dichloromethane solution to check whether residual solvent remained or not. Matrix-assisted laser desorption ionization time-of-flight mass spectra (MALDI-TOF MS) were obtained by a Shimadzu AXIMA-CFR Plus station. Thermogravimetric analysis (TGA) was performed with a Hitachi High-Tech Science TG/DTA 6200 under nitrogen flow at a heating rate of 10 °C min^{-1}.

Differential scanning calorimetry (DSC) was measured using Hitachi High-Tech Science DSC7000X with liquid nitrogen cooling accessory under nitrogen gas flow at different scan rates, where noted. Optical microscopy images were obtained under with or without polarized light conditions using an Olympus BX51 optical microscopy, with thin solvent-free liquid layer sandwiched between two glass plates for the measurement. Small and wide angle X-ray scattering (SWAXS) measurements were performed using Anton Paar SAXSess mc^2 instrument. Attenuated reflection infrared (ATR-IR) spectra were recorded on a Thermo Scientific Nicolet iS5 FT-IR spectrometer equipped with iD5 ATR accessory (diamond prism). The flash-photolysis time resolved microwave conductivity (FP-TRMC) measurement was carried out using an X-band (9.1 GHz) microwave circuit and a nanosecond laser irradiation at 355 nm with photon density of 9.1 x 10^{15} photons cm^{-2} $pulse^{-1}$. FP-TRMC samples were prepared by pasting the samples on a quartz plate.

Rheology experiments were carried out using an Anton Paar Physica MCR301 rheometer, using the parallel plate geometry (25 mm diameter) and a measuring sample thickness of 0.25 mm. For all samples, strain amplitude scans were firstly performed to determine the linear-viscoelastic region. For all samples the strain amplitude of 0.25 was within this linear-viscoelastic region.

For all optical absorption and fluorescence spectroscopy measurements, thin solvent-free liquid samples were sandwiched by two quartz plates and solid film samples were prepared by drop casting a dichloromethane solution on a quartz substrate. UV-Vis absorption and fluorescence spectra in both solution and solvent-free liquid state were recorded on a JASCO V-670 spectrophotometer and a JASCO FP-8300 spectrophotometer, respectively. Absolute quantum yields were determined on a Hamamatsu Photonics absolute PL quantum yield spectrometer C11347. Refractive index (RI) was measured using a Kyoto Electronics RA-600 Refractometer with LED Na-D 589.3 nm at 20 °C. The temperature-dependent luminescence at high temperatures was measured using an intensified multichannel spectrometer MCPD-7000 (Otsuka Electronics, Japan). The samples were positioned on a heater stage THMS600 (Linkam Scientific Instruments). An xyz stage was used to cancel the

shift resulting from the sample movement caused by the temperature. Each sample was heated from 30 to 100 °C at a heating rate of 10 °C min^{-1} and held at each temperature for 5 min for thermal equilibrium. The temperature-dependent luminescence at low temperatures was measured from 10 to –200 °C by a monochromator with a 1200 lines/mm diffraction grating (JASCO CT-25S) and a CCD (TOSHIBA Corporation IK-627).

All theoretical calculations were performed using Sparton’14 program[6] with default thresholds and algorithm. The geometry optimizations for simplified models of **1**, **2**, **3** and **5**, with 2-hexyl-decyl chains replaced by methyl groups, in the ground state were performed at the B3LYP/6-31G* level of theory in the gas phase. The C_1 symmetry for 1 and 2, C_{2h} symmetry for 3, and C_2 symmetry for 5 were employed for the geometry optimization. The Cartesian coordinates of optimized structures are summarized in Supplementary Tables 3–6.

2. Supplementary Tables

Supplementary Table 1. Summarized SWAXS data of **1–5**.

**Compound**	***d*** (Å)		***FWHM***[a] (Å)	**Domain Size**[c] (nm)
	Aromatic Halo	Aliphatic Halo	Aromatic Halo	
**1**	17.7	4.4	25.8	1.50
**2**	16.7	4.4	14.2	2.02
**3**	20.4	4.5	6.7[b]	5.65
**4**	22.3	4.5	7.3[b]	6.18
**5**	18.5	4.5	8.7	3.69

[a] FWHM: full width of half maximum.
[b] The symmetric alkyl chain substitution pattern in **3** and **4** makes their molecular structure of more globular shape, resulting in sharper aromatic halos.
[c] Determined by Scherrer equation[7]. The domain sizes estimated here are much smaller than those of reported locally ordered clusters in isotropic liquid[8], which further confirms the absence of long-range ordered structures.

Supplementary Table 2. Photophysical and optical parameters (λ: wavelength; ε: molar absorption coefficient; Φ_{FL}: fluorescence quantum yield) for **1–7** in solution and solvent-free state.

Compound	Absorption Feature λ_{abs}, nm (ε, 10^4 dm^3 mol^{-1} cm^{-1})		Fluorescence λ_{max}, nm (Φ_{FL})		Refractive Index[c] nD (20 °C)
	Solution[a] (10 μM)	Solvent-free[b]	Solution[a] (1 μM)	Solvent-free[b]	Liquid
**1**	242 (4.61)<br>279 (3.62)<br>345 (3.23)	239<br>280<br>346	382<br>(0.20)	472<br>(0.72)	1.571
**2**	243 (4.84)<br>276 (3.68)<br>341 (3.13)	245<br>279<br>347	387<br>(0.34)	467<br>(0.66)	1.569
**3**	257 (3.93)<br>297 (4.48)<br>382 (4.18)	262<br>296<br>373	424<br>(0.84)	456<br>(0.67)	1.534
**4**	261 (4.50)<br>298 (4.96)<br>385 (4.47)	263<br>296<br>372	424<br>(0.88)	456<br>(0.85)	1.516
**5**	247 (5.39)<br>291 (5.27)<br>377 (4.42)	247<br>292<br>380	419<br>(0.63)	441<br>(0.57)	1.531
**6**	244 (3.88)<br>280 (3.42)<br>344 (2.88)	240<br>282<br>350	381<br>(0.18)	475<br>(0.69)	—
**7**	261 (3.49)<br>298 (4.00)<br>383 (3.54)	263<br>302<br>389	423<br>(0.85)	468<br>(0.32)	—

[a] Prepared in *n*-hexane for **2** and in dichloromethane for all other compounds.
[b] Prepared by sandwiching the liquid between two quartz plates for **1**–**5** and by drop casting the dichloromethane solution on a quartz substrate for **6** and **7**.
[c] Determined at 20 °C with a LED Na-D 589.3 nm light. The order of RI values for pyrene liquids was **1** (1.571) ≈ **2** (1.569) > **3** (1.534) ≈ **5** (1.531) > **4** (1.516), which, basically, is in good accordance with enhanced pyrene/chain ratio, *i.e.*, increased pyrene density.

Supplementary Table 3. Cartesian coordinates [Å] of the optimized structure of simplified model for **1**, with 2-hexyl-decyl chains replaced by methyl groups.

atom	x	y	z	atom	x	y	z
C	0.721298528	0.068510972	0.090525701	C	-1.794400123	0.498036237	0.13580075
C	1.160319597	2.859105117	0.128776227	C	-4.489354715	-0.298281356	0.094732514
C	2.051929237	0.594630924	0.033983769	C	-2.659503511	0.896913189	1.15288808
C	-0.377907027	0.96466472	0.13816341	C	-2.276690237	-0.302479391	-0.920543777
C	-0.129529795	2.344555022	0.175108557	C	-3.61610204	-0.690482889	-0.933000646
C	2.270260894	2.006287498	0.044738958	C	-4.005097767	0.493555199	1.133293014
H	-0.977563464	3.022301318	0.214225552	H	-2.309421795	1.502494867	1.982091739
C	3.173978275	-0.288482671	-0.029282754	H	-5.519450113	-0.627276482	0.040547025
C	5.382052761	-2.029585582	-0.161889189	O	-4.761892024	0.924952202	2.184066477
C	2.976982742	-1.702474472	-0.014669268	C	-6.121469095	0.52403776	2.238602373
C	4.500995583	0.237642794	-0.103506488	H	-6.68930722	0.902661378	1.378376434
C	5.58614319	-0.650908135	-0.171693447	H	-6.524684405	0.956805834	3.156053572
C	4.093094045	-2.552175651	-0.08171652	H	-6.216999296	-0.56901105	2.278829009
H	6.234678057	-2.701430544	-0.213980776	H	3.939613369	-3.628607859	-0.068442762
C	0.564464841	-1.362115914	0.136498246	H	6.594917621	-0.248962397	-0.22987954
C	1.635507509	-2.201357449	0.084431654	H	1.315907304	3.935202682	0.143715564
H	-0.436136563	-1.768721037	0.228080651	H	-1.603051585	-0.583399257	-1.720264079
H	1.487895458	-3.278027842	0.128334451	O	-4.190344555	-1.452221188	-1.911062112
C	3.614772521	2.506395055	-0.027644115	C	-3.37383541	-1.892838093	-2.984717079
C	4.680324553	1.66315024	-0.101996544	H	-4.026605949	-2.477372609	-3.635829996
H	3.762625535	3.583726929	-0.021767695	H	-2.54991786	-2.527088313	-2.631811957
H	5.692889655	2.055725338	-0.156970218	H	-2.959111685	-1.048248647	-3.550255402

Supplementary Table 4. Cartesian coordinates [Å] of the optimized structure of simplified model for **2**, with 2-hexyl-decyl chains replaced by methyl groups.

atom	x	y	z	atom	x	y	z
C	0.50899669	-0.072690629	-0.003246474	H	-2.033698735	0.183854437	0.080086065
C	0.748148714	2.256464278	1.582071004	C	-4.694122331	-0.611920375	-0.451617403
C	1.798863763	0.416406686	0.380002308	C	-2.921079981	-0.115297745	1.118204843
C	-0.649309027	0.63021327	0.414796403	C	-2.507071983	0.092043775	-1.247393311
C	-0.502008985	1.776902182	1.209716529	C	-3.818231122	-0.304685501	-1.495955325
C	1.916730154	1.596599552	1.176753658	C	-4.241474764	-0.510115238	0.867296445
H	-1.395895427	2.306777755	1.527339299	H	-2.587479302	-0.063535922	2.149974797
C	2.981021947	-0.276823721	-0.025023693	H	-4.148766494	-0.374069048	-2.52822653
C	5.307485685	-1.644095452	-0.825998303	H	-5.707558259	-0.922831564	-0.678009996
C	2.881743008	-1.467021031	-0.806656458	O	-1.677679884	0.354011176	-2.321352935
C	4.269196345	0.213891584	0.352474136	O	-4.996329967	-0.778486177	1.975275016

C	5.415352244	-0.484660887	-0.060077851	C	-6.339047084	-1.190168775	1.783278472
C	4.055928411	-2.132238191	-1.195436713	H	-6.931100024	-0.416987579	1.274775982
H	6.205586333	-2.171666496	-1.136144478	H	-6.747544566	-1.356901689	2.781814175
C	0.447517689	-1.284361708	-0.776700714	H	-6.397863678	-2.123731952	1.207681543
C	1.574921774	-1.944372222	-1.158217086	C	-1.564835941	1.734602243	-2.664001679
H	-0.524587738	-1.673714738	-1.054694206	H	-0.882499653	1.785814627	-3.515742315
H	1.499895014	-2.858913577	-1.741872499	H	-1.153548615	2.322582147	-1.834108755
C	3.224220416	2.067461815	1.540072106	H	-2.540265172	2.151150665	-2.951526533
C	4.347915866	1.40912926	1.145899289	H	3.977271073	-3.039084532	-1.790443654
H	3.295993775	2.969362471	2.143420255	H	6.3946515	-0.109383366	0.227450715
H	5.331223913	1.777399954	1.428907044	H	0.826236175	3.15429648	2.190663647

Supplementary Table 5. Cartesian coordinates [Å] of the optimized structure of simplified model for **3**, with 2-hexyl-decyl chains replaced by methyl groups.

atom	x	y	z	atom	x	y	z
C	1.428312118	0.139113436	1.240908158	C	3.657994169	0.35055669	-2.472248797
C	2.836686011	0.297057531	-1.227402397	C	5.291757564	0.438006365	-4.757210899
C	0.715546472	0.069476548	0	C	3.646697563	-0.72227247	-3.38744232
C	2.836686011	0.297057531	1.227402397	C	4.485888833	1.447614908	-2.701639776
C	3.49728994	0.392346274	0	C	5.300512533	1.490264828	-3.845047549
C	1.428312118	0.139113436	-1.240908158	C	4.460340962	-0.668633184	-4.519009387
H	4.578484383	0.494376786	0	H	3.022021789	-1.583078452	-3.184505322
C	-0.715546472	-0.069476548	0	H	4.50528751	2.287385257	-2.015505915
C	-3.49728994	-0.392346274	0	H	5.903759548	0.433199532	-5.650371556
C	-1.428312118	-0.139113436	1.240908158	O	-4.528739364	1.650120521	5.467209691
C	-1.428312118	-0.139113436	-1.240908158	O	-6.065564253	-2.612954527	3.978279924
C	-2.836686011	-0.297057531	-1.227402397	O	6.065564253	2.612954527	3.978279924
C	-2.836686011	-0.297057531	1.227402397	O	4.528739364	-1.650120521	5.467209691
H	-4.578484383	-0.494376786	0	O	6.065564253	2.612954527	-3.978279924
C	0.676248253	0.072685954	2.460651671	O	4.528739364	-1.650120521	-5.467209691
C	-0.676248253	-0.072685954	2.460651671	O	-4.528739364	1.650120521	-5.467209691
H	1.208219413	0.13981412	3.402659122	O	-6.065564253	-2.612954527	-3.978279924
H	-1.208219413	-0.13981412	3.402659122	C	-6.906178428	-2.720324699	5.115260206
C	0.676248253	0.072685954	-2.460651671	H	-6.327044738	-2.706302196	6.048239422
C	-0.676248253	-0.072685954	-2.460651671	H	-7.416160379	-3.680874406	5.020911017
H	1.208219413	0.13981412	-3.402659122	H	-7.651911008	-1.914654833	5.144502474
H	-1.208219413	-0.13981412	-3.402659122	C	-3.716343236	2.801229565	5.30078199
C	-3.657994169	-0.35055669	-2.472248797	H	-3.917409963	3.436338434	6.165686394
C	-5.291757564	-0.438006365	-4.757210899	H	-3.97096601	3.347287734	4.382834788
C	-3.646697563	0.72227247	-3.38744232	H	-2.649265541	2.543276521	5.279716564

C	-4.485888833	-1.447614908	-2.701639776	C	3.716343236	-2.801229565	5.30078199
C	-5.300512533	-1.490264828	-3.845047549	H	2.649265541	-2.543276521	5.279716564
C	-4.460340962	0.668633184	-4.519009387	H	3.917409963	-3.436338434	6.165686394
H	-3.022021789	1.583078452	-3.184505322	H	3.97096601	-3.347287734	4.382834788
H	-4.50528751	-2.287385257	-2.015505915	C	6.906178428	2.720324699	5.115260206
H	-5.903759548	-0.433199532	-5.650371556	H	7.416160379	3.680874406	5.020911017
C	-3.657994169	-0.35055669	2.472248797	H	7.651911008	1.914654833	5.144502474
C	-5.291757564	-0.438006365	4.757210899	H	6.327044738	2.706302196	6.048239422
C	-4.485888833	-1.447614908	2.701639776	C	3.716343236	-2.801229565	-5.30078199
C	-3.646697563	0.72227247	3.38744232	H	3.97096601	-3.347287734	-4.382834788
C	-4.460340962	0.668633184	4.519009387	H	3.917409963	-3.436338434	-6.165686394
C	-5.300512533	-1.490264828	3.845047549	H	2.649265541	-2.543276521	-5.279716564
H	-4.50528751	-2.287385257	2.015505915	C	6.906178428	2.720324699	-5.115260206
H	-3.022021789	1.583078452	3.184505322	H	7.416160379	3.680874406	-5.020911017
H	-5.903759548	-0.433199532	5.650371556	H	6.327044738	2.706302196	-6.048239422
C	3.657994169	0.35055669	2.472248797	H	7.651911008	1.914654833	-5.144502474
C	5.291757564	0.438006365	4.757210899	C	-6.906178428	-2.720324699	-5.115260206
C	4.485888833	1.447614908	2.701639776	H	-7.651911008	-1.914654833	-5.144502474
C	3.646697563	-0.72227247	3.38744232	H	-7.416160379	-3.680874406	-5.020911017
C	4.460340962	-0.668633184	4.519009387	H	-6.327044738	-2.706302196	-6.048239422
C	5.300512533	1.490264828	3.845047549	C	-3.716343236	2.801229565	-5.30078199
H	4.50528751	2.287385257	2.015505915	H	-3.917409963	3.436338434	-6.165686394
H	3.022021789	-1.583078452	3.184505322	H	-2.649265541	2.543276521	-5.279716564
H	5.903759548	0.433199532	5.650371556	H	-3.97096601	3.347287734	-4.382834788

Supplementary Table 6. Cartesian coordinates [Å] of the optimized structure of simplified model for **5**, with 2-hexyl-decyl chains replaced by methyl groups.

atom	x	y	z	atom	x	y	z
C	1.537830114	-1.106467831	-0.198169378	C	3.876502835	-2.131697997	-0.180548313
C	2.727618927	1.479257046	-0.14900857	C	5.70514381	-4.286478006	-0.241598564
C	0.71555769	0.064149852	-0.194114242	C	4.798430189	-2.269041071	-1.222407443
C	2.947145201	-0.963760648	-0.177969422	C	3.897033686	-3.090190634	0.857332517
C	3.503372301	0.317254312	-0.163792929	C	4.798948306	-4.14899888	0.813120149
C	1.31669964	1.364675197	-0.187211763	C	5.710060076	-3.332250852	-1.262904988
H	4.584040848	0.413385928	-0.133884162	H	4.807592095	-1.550062358	-2.035889601
C	-0.71555769	-0.064149852	-0.194114242	H	4.783085802	-4.874449164	1.621425104
C	-3.503372301	-0.317254312	-0.163792929	H	6.38940121	-5.127066039	-0.248237037
C	-1.31669964	-1.364675197	-0.187211763	O	-4.559865873	-2.440730962	-2.047263996
C	-1.537830114	1.106467831	-0.198169378	O	-3.601533099	-5.643436638	2.382740783
C	-2.947145201	0.963760648	-0.177969422	O	3.008901374	-3.02333854	1.915522789

C	-2.727618927	-1.479257046	-0.14900857
H	-4.584040848	-0.413385928	-0.133884162
C	0.898825709	-2.388307619	-0.261131704
C	-0.455362947	-2.510921793	-0.254562025
H	1.51887293	-3.274867957	-0.325154501
H	-0.906313563	-3.494903034	-0.315080506
C	0.455362947	2.510921793	-0.254562025
C	-0.898825709	2.388307619	-0.261131704
H	0.906313563	3.494903034	-0.315080506
H	-1.51887293	3.274867957	-0.325154501
C	-3.410091837	-2.798869806	-0.012868262
C	-4.728713183	-5.252835907	0.357500604
C	-3.15294981	-3.594842503	1.114600223
C	-4.348658579	-3.250817921	-0.961805497
C	-4.998611445	-4.476663955	-0.766255016
C	-3.801435215	-4.817776635	1.30751191
H	-2.438155034	-3.226301256	1.840996834
H	-5.719624156	-4.840264118	-1.489311833
H	-5.231909603	-6.202394132	0.511081157
C	-3.876502835	2.131697997	-0.180548313
C	-5.70514381	4.286478006	-0.241598564
C	-4.798430189	2.269041071	-1.222407443
C	-3.897033686	3.090190634	0.857332517
C	-4.798948306	4.14899888	0.813120149
C	-5.710060076	3.332250852	-1.262904988
H	-4.807592095	1.550062358	-2.035889601
H	-4.783085802	4.874449164	1.621425104
H	-6.38940121	5.127066039	-0.248237037
C	3.410091837	2.798869806	-0.012868262
C	4.728713183	5.252835907	0.357500604
C	4.348658579	3.250817921	-0.961805497
C	3.15294981	3.594842503	1.114600223
C	3.801435215	4.817776635	1.30751191
C	4.998611445	4.476663955	-0.766255016
H	2.438155034	3.226301256	1.840996834
H	5.719624156	4.840264118	-1.489311833
H	5.231909603	6.202394132	0.511081157

O	6.552678455	-3.348879869	-2.340762229
O	4.559865873	2.440730962	-2.047263996
O	3.601533099	5.643436638	2.382740783
O	-3.008901374	3.02333854	1.915522789
O	-6.552678455	3.348879869	-2.340762229
C	-2.660679836	-5.239912823	3.362739332
H	-1.654376268	-5.125675415	2.937646527
H	-2.647235248	-6.034492462	4.111558496
H	-2.954720899	-4.296262013	3.842435108
C	-5.471088823	-2.874188203	-3.040444133
H	-5.475973655	-2.09269617	-3.802859494
H	-6.486537363	-2.992870962	-2.638016085
H	-5.153208865	-3.822031471	-3.495882488
C	7.48899228	-4.407971171	-2.436388958
H	8.183065278	-4.414641077	-1.584748958
H	8.050296356	-4.230512458	-3.355888289
H	6.991174097	-5.385203978	-2.499886547
C	3.381217876	-2.108506656	2.944874344
H	2.599966459	-2.166946106	3.706940501
H	3.442428044	-1.080794544	2.566982857
H	4.346628339	-2.387347956	3.389643009
C	5.471088823	2.874188203	-3.040444133
H	5.475973655	2.09269617	-3.802859494
H	6.486537363	2.992870962	-2.638016085
H	5.153208865	3.822031471	-3.495882488
C	2.660679836	5.239912823	3.362739332
H	1.654376268	5.125675415	2.937646527
H	2.647235248	6.034492462	4.111558496
H	2.954720899	4.296262013	3.842435108
C	-3.381217876	2.108506656	2.944874344
H	-2.599966459	2.166946106	3.706940501
H	-3.442428044	1.080794544	2.566982857
H	-4.346628339	2.387347956	3.389643009
C	-7.48899228	4.407971171	-2.436388958
H	-8.183065278	4.414641077	-1.584748958
H	-8.050296356	4.230512458	-3.355888289
H	-6.991174097	5.385203978	-2.499886547

3. Supplementary Figures

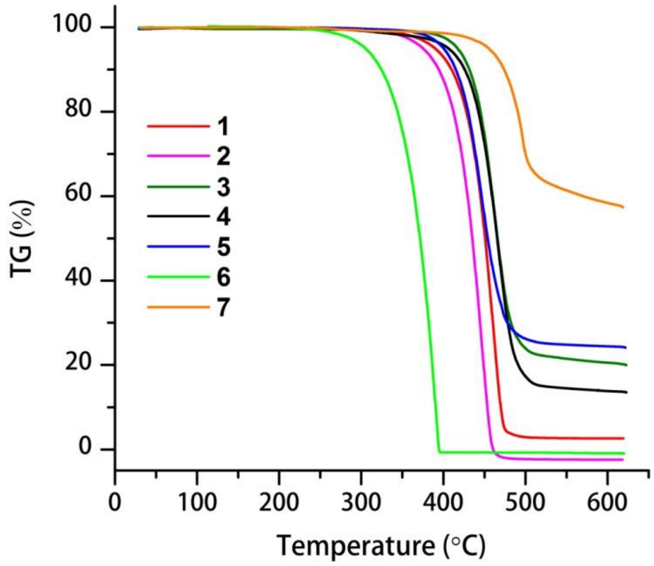

Supplementary Figure 26. Thermogravimetric analysis of **1**–**7**. The mass loss% at 300 °C were calculated as 0.52% (**1**), 0.33% (**2**), 0.62% (**3**), 0.83% (**4**), 0.33% (**5**), 4.17% (**6**) and 0.73% (**7**).

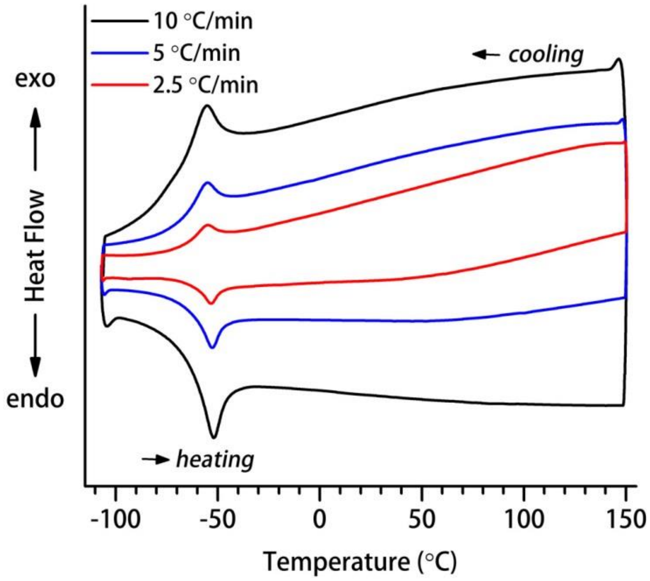

Supplementary Figure 27. DSC thermograms of **4** showing its *solid*-to-*liquid* melting (solidification) phase transition processes at different scan rates as noted in the figure.

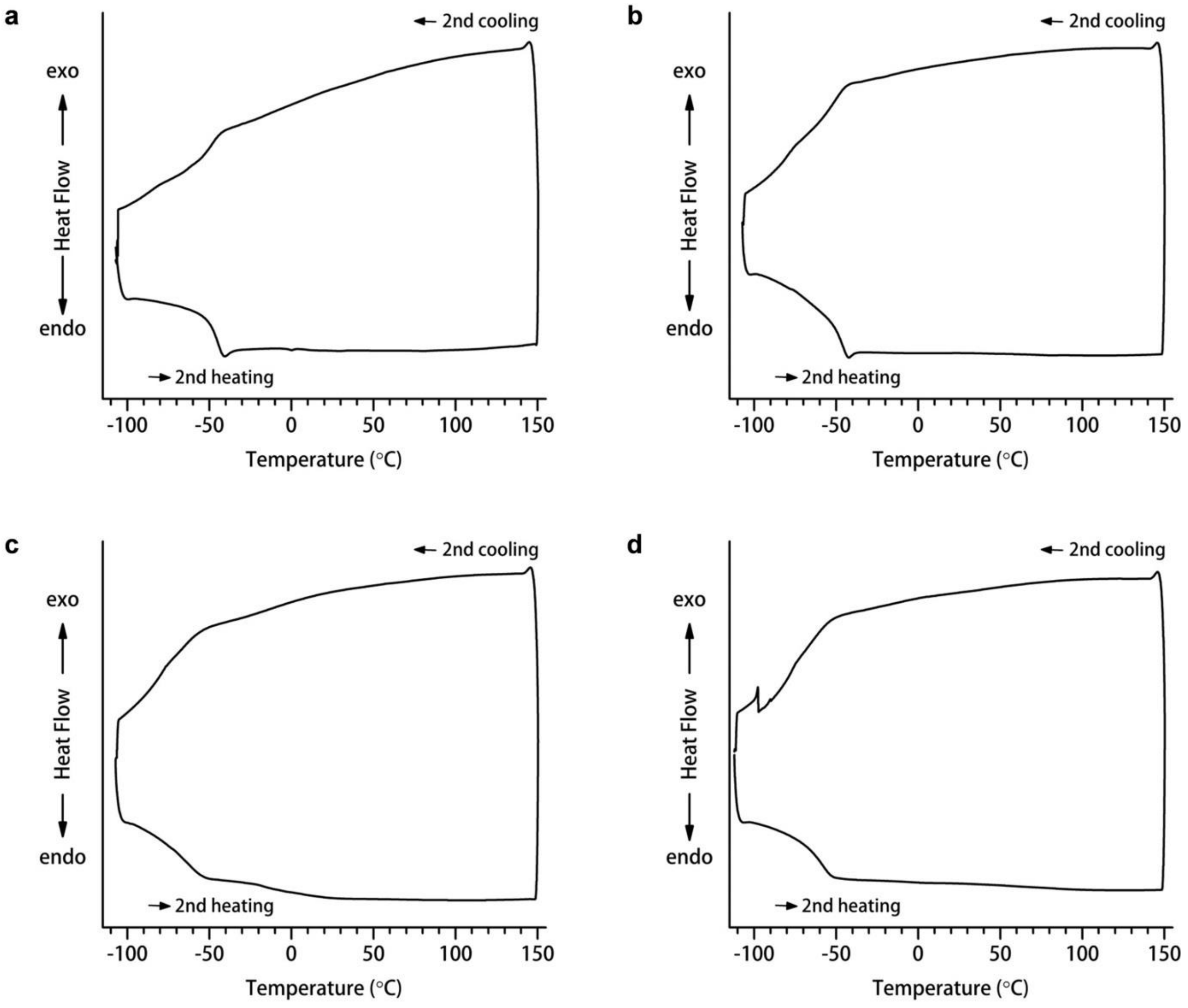

Supplementary Figure 28. DSC thermograms of (**a**) **1**, (**b**) **2**, (**c**) **3** and (**d**) **5** in the second heating and cooling trace at a scanning rate of 10 °C min^{-1}. During cooling, all samples show stepwise *isotropic*-to-*glass* transition, indicating that the molecular motion frozen part by part. During heating, sample **3** (**c**) also exhibits stepwise *glass*-to-*isotropic* transitions which can be attributed to its high viscosity. Under highly viscous local environment, relaxation of molecular motion during *glass*-to-*isotropic* transition is trapped, leading to stepwise phase transition and requiring more energy consuming which is reflected in its relatively high heat capacity (see Table 1 in the main manuscript) compared with that of other molecules.

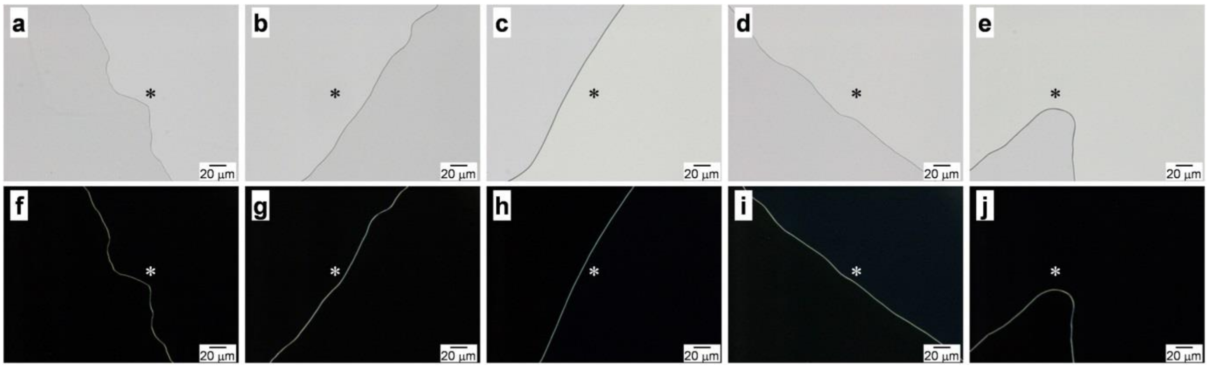

Supplementary Figure 29. Optical microscopy (OM) (up) and polarized optical microscopy (POM) (below) images of **1** (**a** and **f**), **2** (**b** and **g**), **3** (**c** and **h**), **4** (**d** and **i**), and **5** (**e** and **j**) taken at room temperature. The sample region was denoted with asterisk (∗).

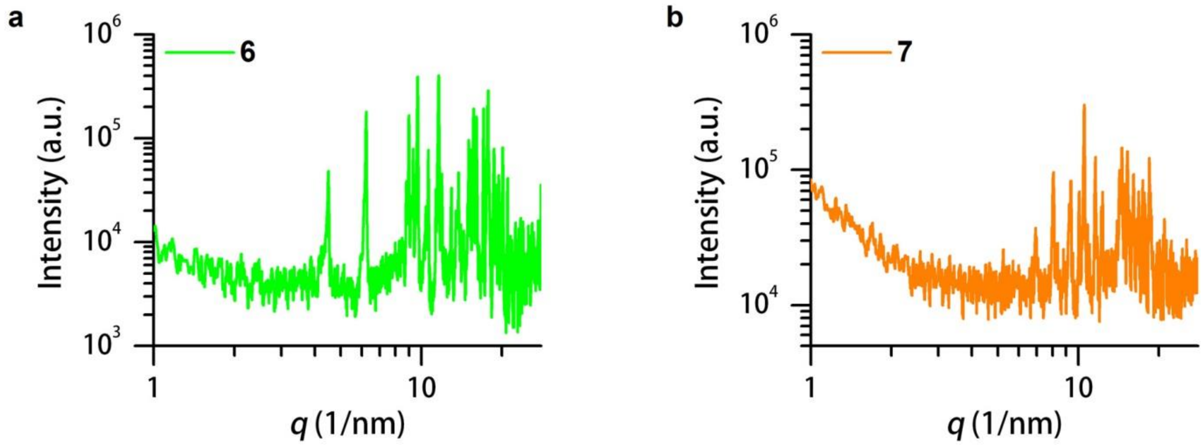

Supplementary Figure 30. Small- and wide-angle X-ray scattering (SWAXS) profiles of (**a**) **6** and (**b**) **7** as solid powder at 25 °C.

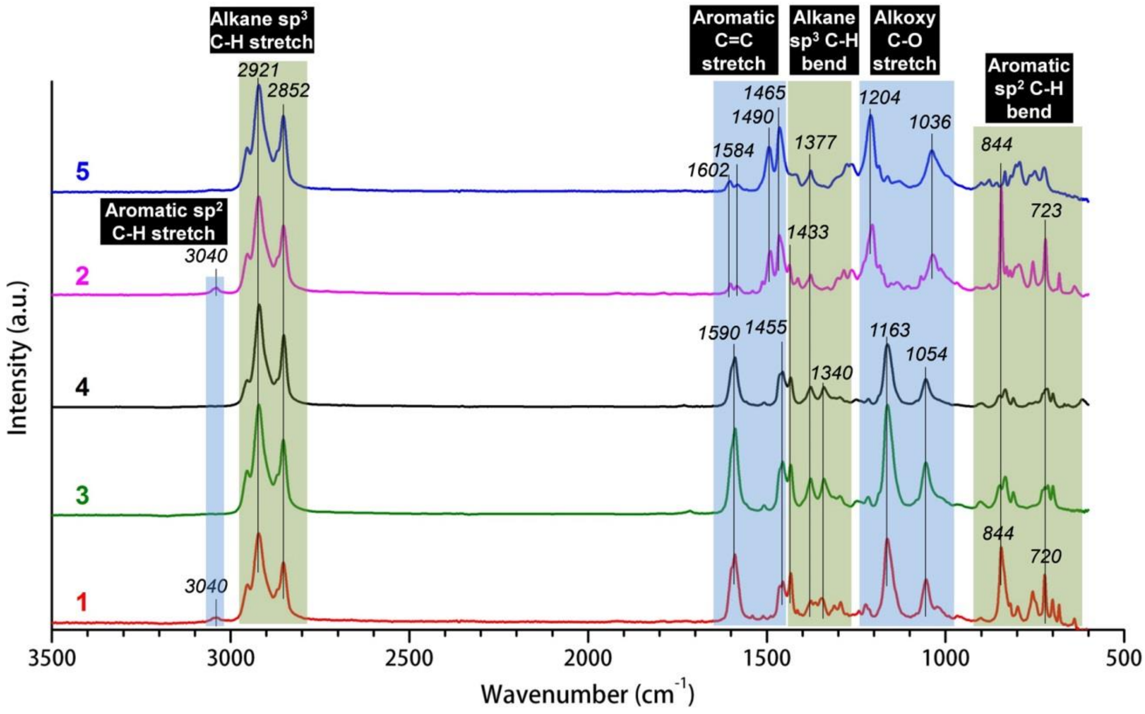

Supplementary Figure 31. ATR-FTIR spectra of **1**–**5** as neat. Peaks of typical functional groups are denoted in the figure. All fluids exhibit asymmetric and symmetric methylene stretching bands at 2921 and 2852 cm^{-1}, respectively, both of which are broader and higher wavenumber-shifted compared to those of all-*trans* conformation of linear alkyl chains residing around 2918 and 2849 cm^{-1} (refs 9,10). Therefore, the alkyl chains in **1**–**5** are in a molten state.

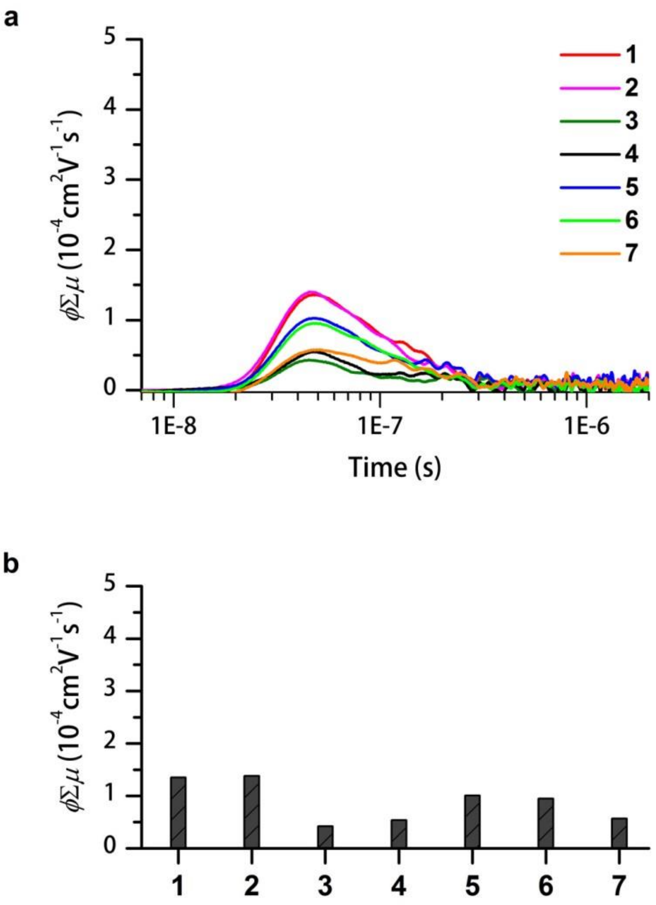

Supplementary Figure 32. (**a**) FP-TRMC transient decay profiles of **1**–**5** at solvent-free neat state as well as **6** and **7** at solid state. (**b**) Maximum $\phi\Sigma\mu$ values (ϕ: charge carrier generation yield, $\Sigma\mu$: sum of hole and electron mobilities) of **1**–**7** under an excitation at λ = 355 nm. All compounds exhibit very low $\phi\Sigma\mu$ values in the order of 10^{-5}–10^{-4} cm^2 V^{-1} s^{-1}. Even though solid **6** and **7** possessed better organization than fluids **1**–**5** based on SWAXS analysis, their crystallinity is comparatively poor, as suggested by the low intensity in their SWAXS profiles (see Supplementary Fig. 30). Therefore, the crystals in **6** and **7** are randomly oriented and fairly amorphous, leading to poor capability for long range charge carrier transport as in **1**–**5**.

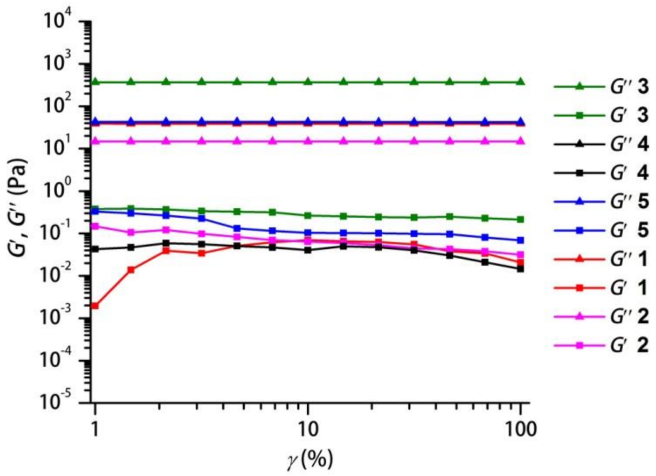

Supplementary Figure 33. Storage elastic modulus (*G*′, square) and viscous loss modulus (*G*″, triangle) of **1**–**5** as a function of strain amplitude (γ) at 25 °C.

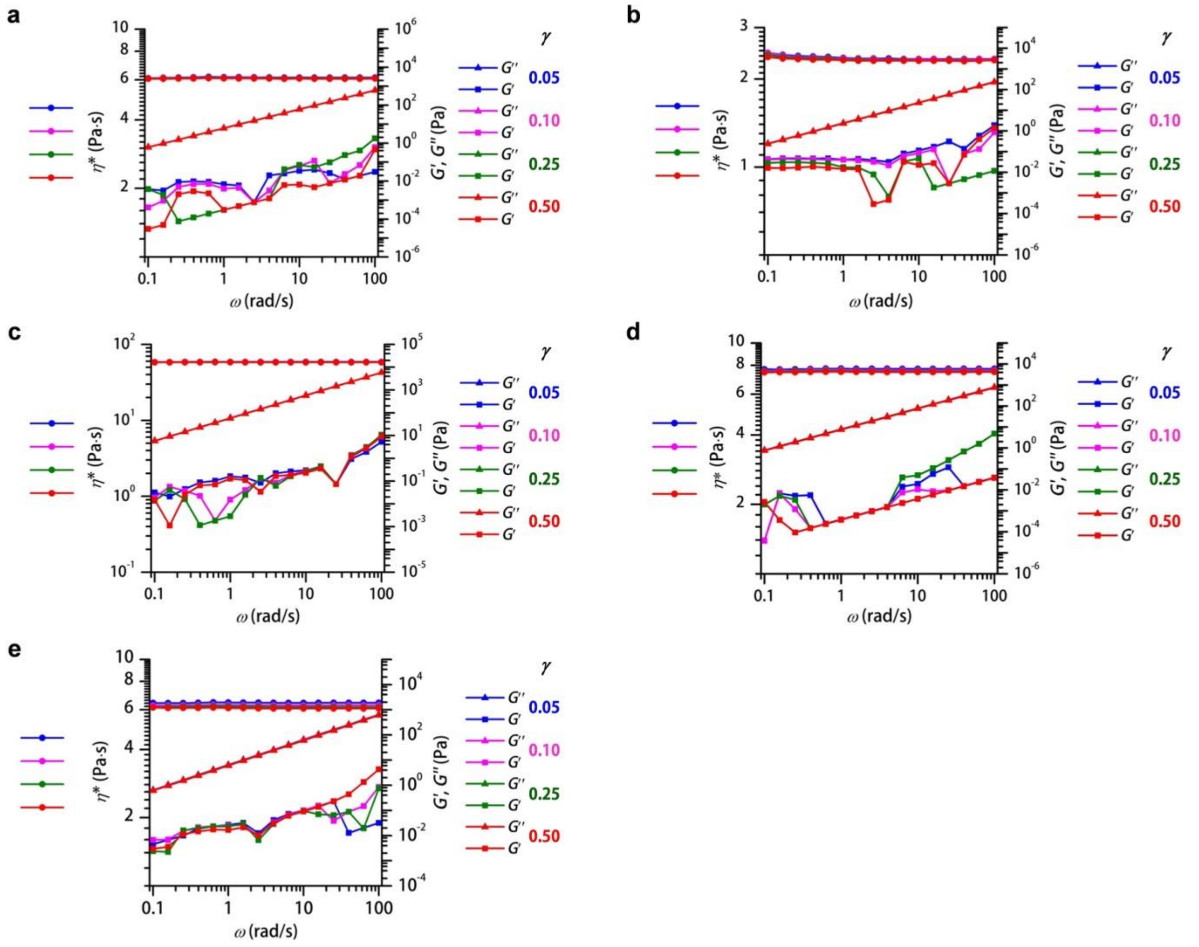

Supplementary Figure 34. Complex viscosity (*η**, circles), storage elastic modulus (*G*′, squares) and viscous loss modulus (*G*″, triangles) of (**a**) **1**, (**b**) **2**, (**c**) **3**, (**d**) **4** and (**e**) **5** as a function of angular frequency (*ω*) with strain amplitude (*γ*) of 0.05 (blue), 0.10 (pink), 0.25 (green) and 0.50 (red) at 25 °C.

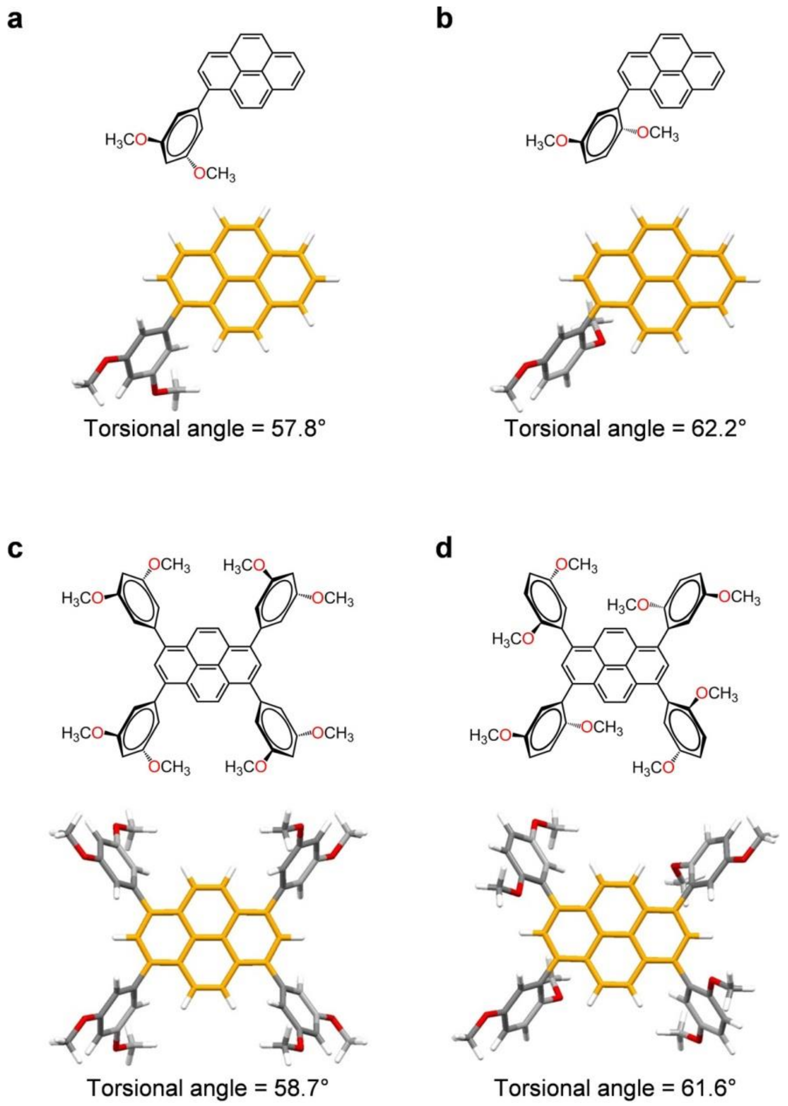

Supplementary Figure 35. Optimized structures of the simplified models for (**a**) **1**, (**b**) **2**, (**c**) **3** and (**d**) **5**, with 2-hexyldecyl chains replaced by methyl groups, in the ground state. The optimized structures suggest larger torsional angle between phenyl unit and pyrene core for (2,5)-substitution motif than for (3,5)-motif, leading to less π-conjugation in **2** (or **5**) than **1** (or **3**).

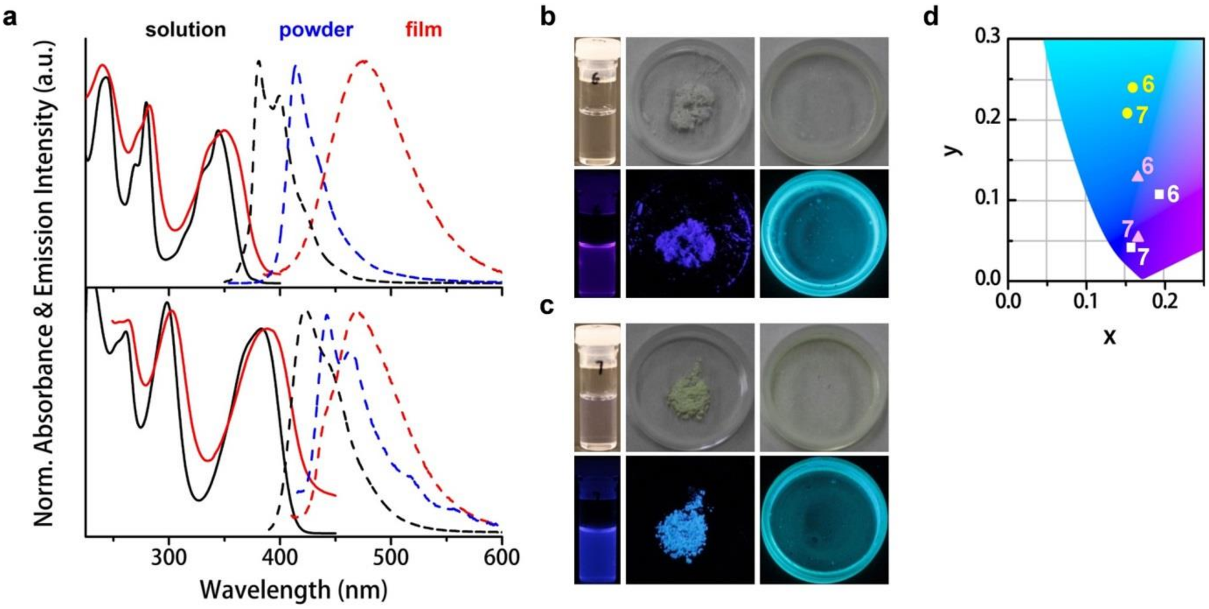

Supplementary Figure 36. (**a**) Normalized UV-vis absorption (solid lines) and fluorescence (dashed lines) spectra of **6** (up) and **7** (below) in dichloromethane solution (black) (UV-vis: 10 μM; FL: 1 μM), powder (blue) and cast film (red) states. λ_{ex}: **6**, 344 nm; **7**, 378 nm. Photo images of solution and solvent-free powder or film samples of (**b**) **6** and (**c**) **7** under visible light (up) and handy UV lamp irradiation at 365 nm (below). (**d**) CIE 1931 chromaticity diagram of **6** and **7** in solution (white squares), powder (pink triangles) and film (yellow circles) states at room temperature. The solid-state fluorescence of **6** and **7** were closely related to sample conditions. Different emission colors were observed in a randomly aggregated powder and a thin cast film.

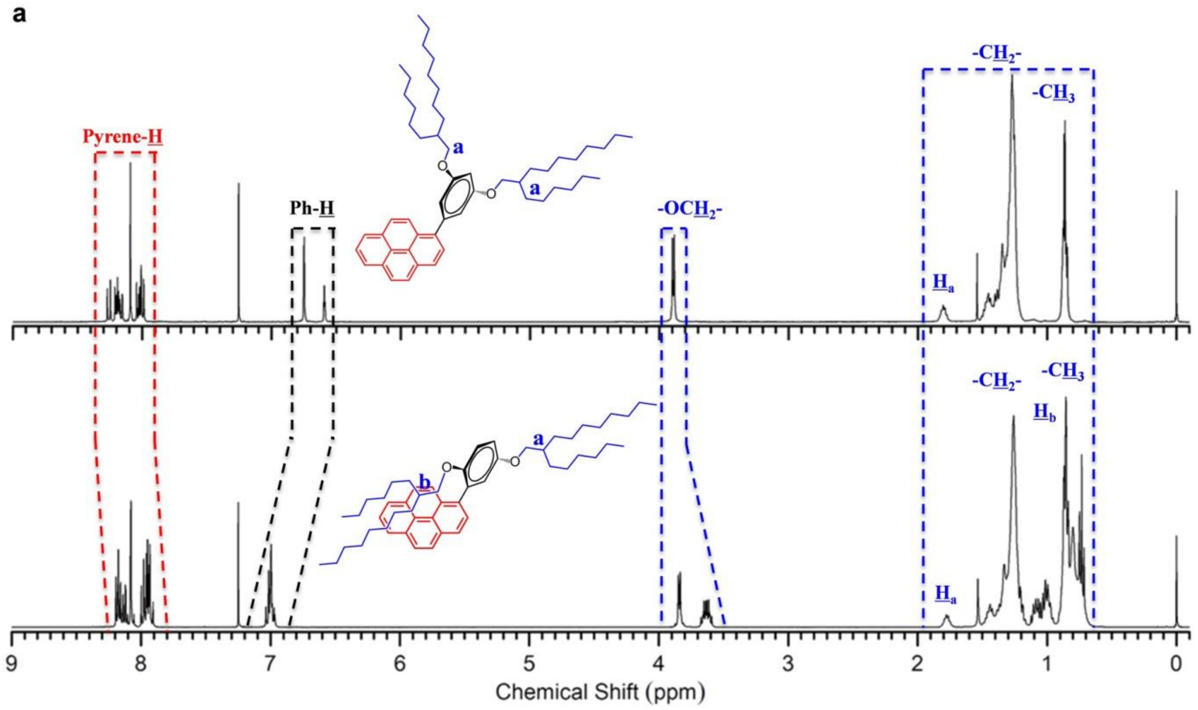

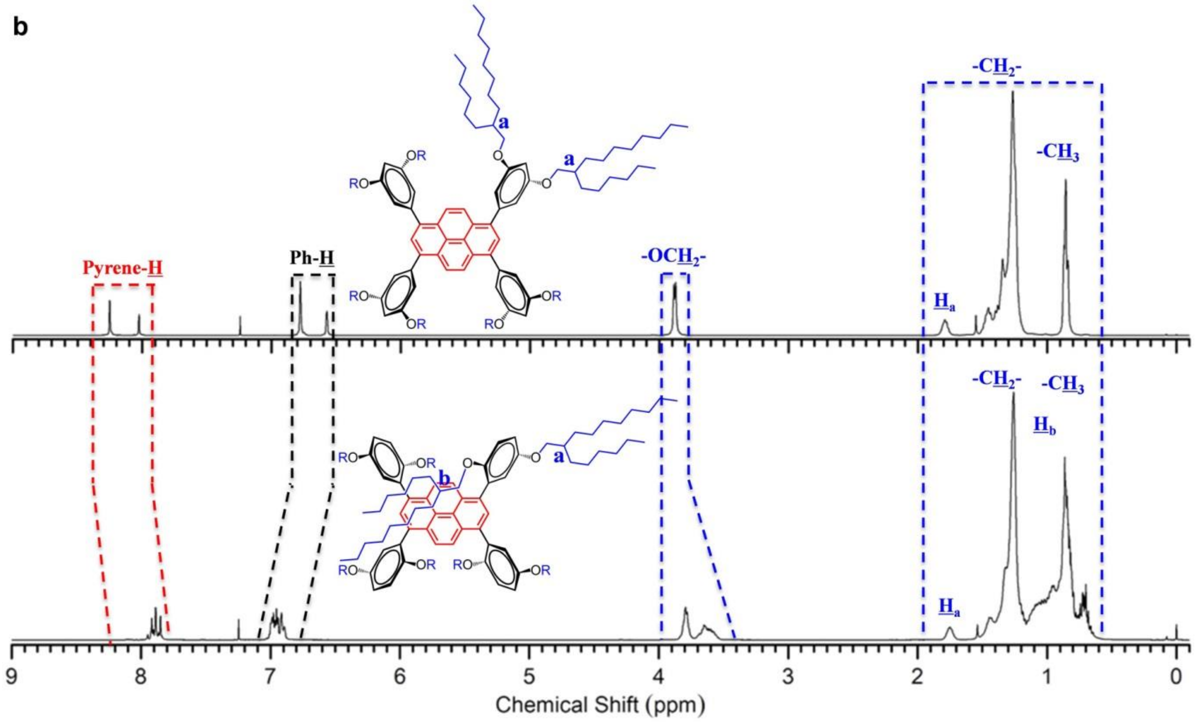

Supplementary Figure 37. ^{1}H NMR spectra of (**a**) **1**(up), **2**(below) and (**b**) **3** (up), **5** (below) in $CDCl_3$ (400 MHz). Compared to the chemical shifts of **1** and **3**, both **2** and **5** exhibit up-field shift of pyrene and alkyl chain protons (H_a, H_b, -CH_2- and -CH_3), down-field shift of phenyl protons and splitting of -OCH_2- on the 2-position substituted alkyl chain. The results strongly confirm that the protons on the alkyl chains at 2-position of the phenyl unit are well shielded by the ring current of pyrene unit.

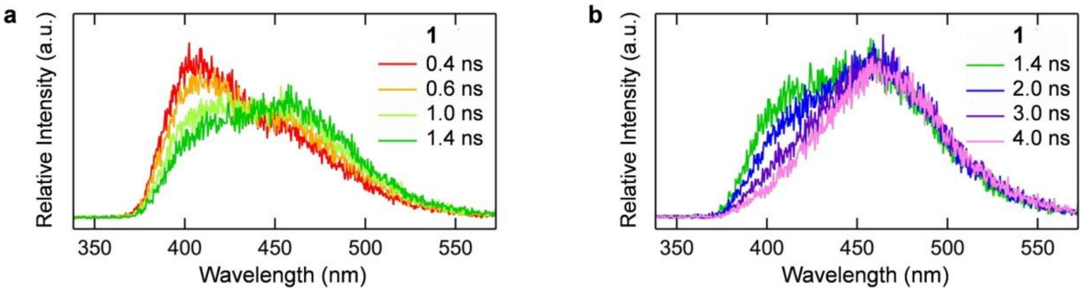

Supplementary Figure 38. (**a**) Picosecond time-resolved fluorescence spectra of liquid **1** acquired at 0.4, 0.6, 1.0 and 1.4 ns. The spectral evolution represents a process during which excimers (~472 nm) formed from excited monomers (~400 nm). (**b**) Picosecond time-resolved fluorescence spectra of liquid **1** acquired at 1.4, 2.0, 3.0 and 4.0 ns. During this spectral evolution, the excited monomers (~400 nm) decay without contributing to excimer formation, since the excimer band (~472 nm) remains unchanged. λ_{ex} = 345 nm.

$$I(\lambda,t)=I_1(\lambda)\exp\left(-\frac{t}{T_1}\right)+I_2(\lambda)\exp\left(-\frac{t}{T_2}\right)+I_3(\lambda)\exp\left(-\frac{t}{T_3}\right)$$

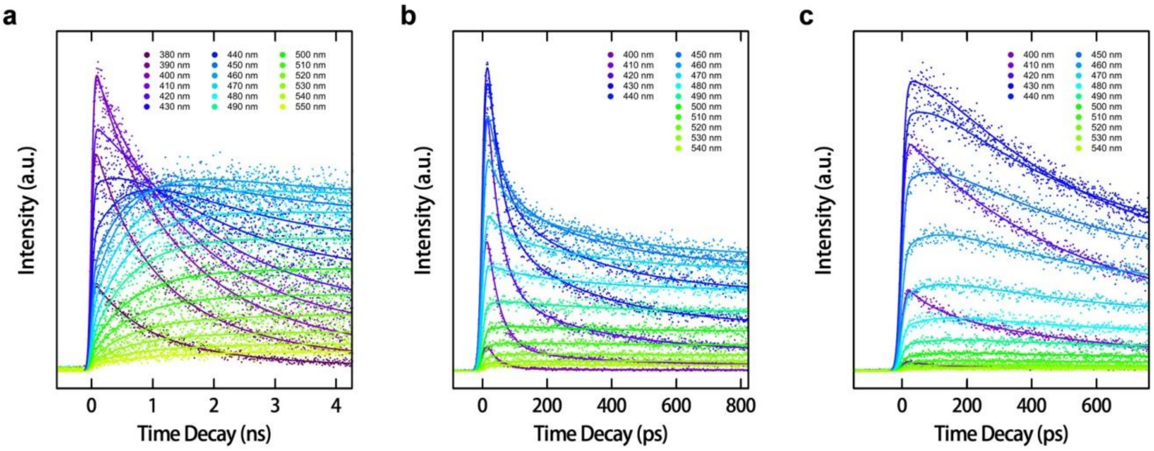

Supplementary Figure 39. Decay traces of (**a**) **1** at 18 different wavelengths, (**b**) **3** at 15 different wavelengths and (**c**) **5** at 15 different wavelengths are simultaneously fitted using three exponential functions with common lifetimes.

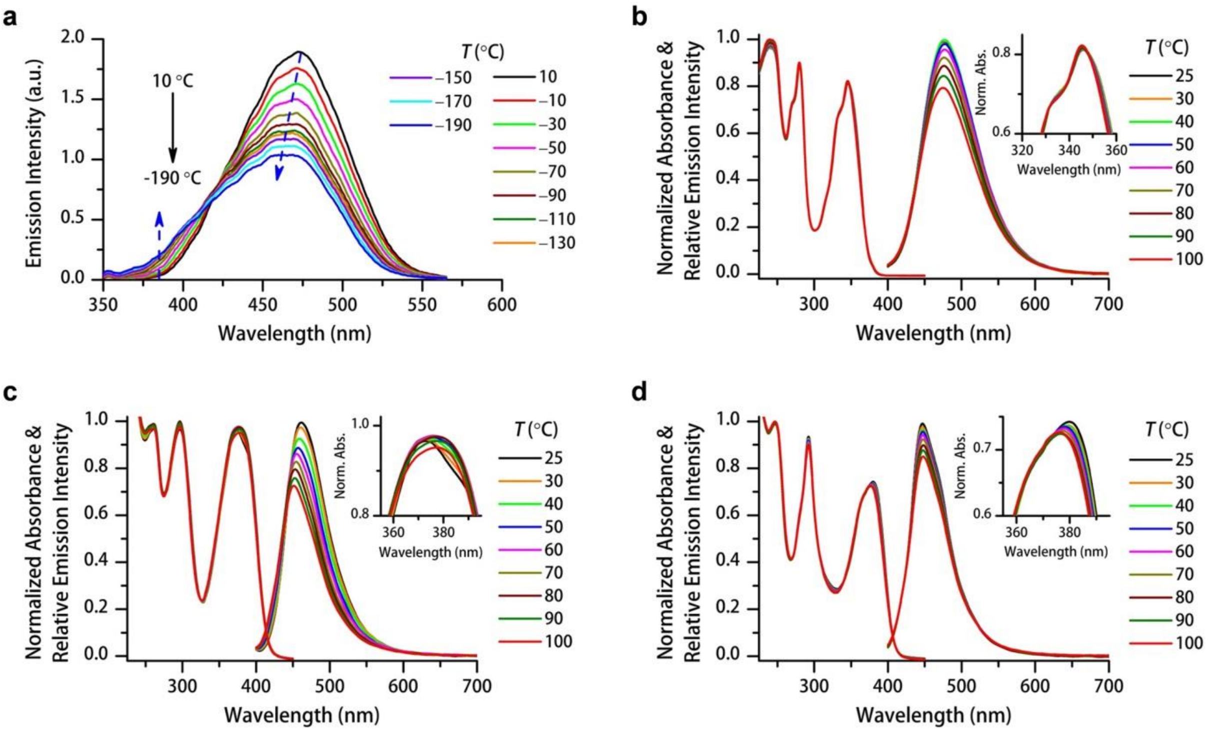

Supplementary Figure 40. (**a**) Fluorescence spectra of **1** as a solvent-free sample measured at decreased temperatures from 10 to –190 °C under vacuum condition. Liquid-state UV-vis (left) and fluorescence (right) spectra of (**b**) **1**, (**c**) **3** and (**d**) **5** measured at increased temperatures from 25 to 100 °C. Enlarged view of the absorption bands at 330–360 nm for **1** (inset of (**b**)) and at 355–395 nm for **3** (inset of (**c**)) and **5** (inset of (**d**)).The sample was sandwiched between two quartz plates. **1**: λ_{ex} = 344 nm; **3**, **5**: λ_{ex} = 378 nm. The decrease in emission intensity originates from accelerated non-radiative decay processes upon heating. The heating induced shape change in absorption band as well as blue-shift in emission band of **3** confirms low stability of its ground-state-dimers.

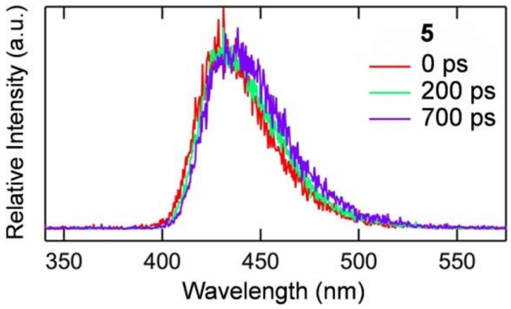

Supplementary Figure 41. Picosecond time-resolved fluorescence spectra of liquid **5** acquired at 0, 200 and 700 ps as denoted inset. The spectra undergo a very small red shift, accompanied by slight broadening of the band, which could be rationalized by intramolecular conformational relaxation and intermolecular reorientation in the excited state. Neither excimer nor excited ground-state-dimer is formed.

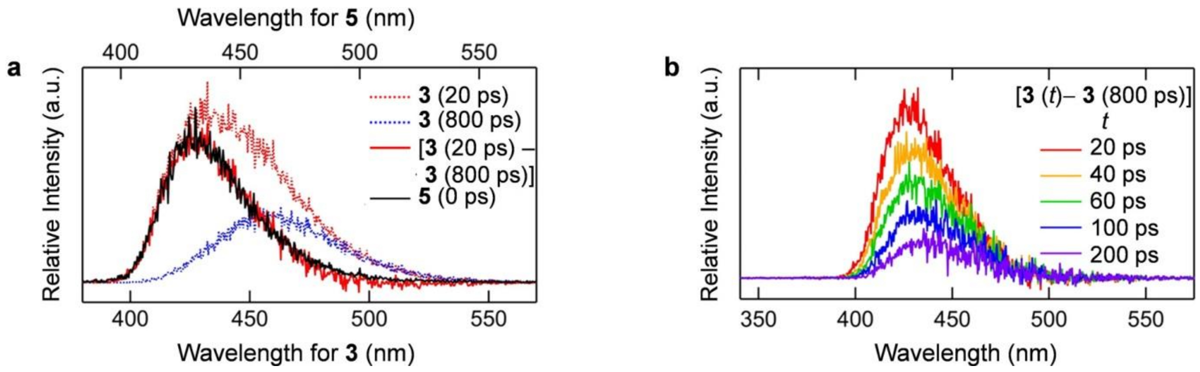

Supplementary Figure 42. Picosecond time-resolved fluorescence spectra of liquid **3** acquired at 20 ps: **3** (20 ps) (red dotted line) and 800 ps: **3** (800 ps) (blue dotted line) and liquid **5** acquired at 0 ps: **5** (0 ps) (black line) with artificial blue-shift of 4 nm (see the actual horizontal axes: up for **5** and below for **3**) as well as an emission spectrum derived by subtracting the spectrum of **3** (800 ps) from the spectrum of **3** (20 ps): [**3** (20 ps) – **3** (800 ps)] (red line). By subtracting the excited ground-state-dimers' spectrum of **3** (800 ps) from the spectrum of **3** (20 ps), the resulting spectrum of [**3** (20 ps) – **3** (800 ps)] can be regarded as monomeric due to its similarity to the monomer spectrum of **5** (0 ps). (**b**) Temporal monomer spectral evolution of **3** by subtracting the spectrum of **3** (800 ps) from the picosecond time-resolved fluorescence spectra of **3** acquired at 20, 40, 60, 100 and 200 ps as denoted inset.

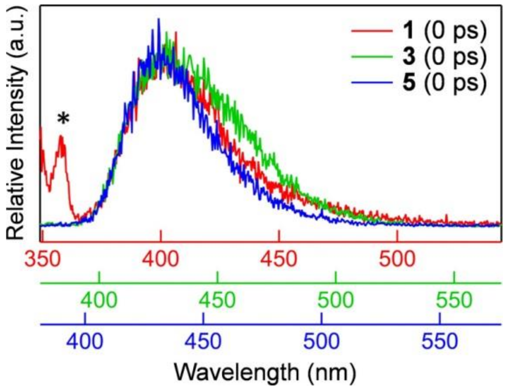

Supplementary Figure 43. Picosecond time-resolved fluorescence spectra of liquids **1** (red line), **3** (green line) and **5** (blue line) acquired at 0 ps. To overlap their spectra, the spectrum of **3** was artificially blue shifted by 27 nm and the spectrum of **5** was artificially blue shifted by 31 nm (see the actual horizontal axes: red for **1**, green for **3** and blue for **5**). Asterisk denotes the stray light signal caused by the excitation pulse.

4. Supplementary References

1. Babu, S. S. *et al.* Nonvolatile liquid anthracenes for facile full-colour luminescence tuning at single blue-light excitation. *Nature Commun.* **4,** 1969 (2013).
2. Chen, J., Kampf, J. W. & McNeil, A. J. Comparing molecular gelators and nongelators based on solubilities and solid-state interactions. *Langmuir* **26,** 13076-13080 (2010).
3. Ellinger, S. *et al.* Donor–acceptor–donor-based π-conjugated oligomers for nonlinear optics and near-IR emission. *Chem. Mater.* **23,** 3805-3817 (2011).
4. Kang, P., Brookhart, M., Meyer, Thomas. & Zhang, S. Production of formate from carbon dioxide with immobilized iridium princer complex catalysts. US Pat. 20150218717 (2015).
5. Xing, C. & Doshi, J. Preparation of substituted chromene derivatives for use as Bcl-2 protein antagonists. PCT Int. 2008118802 (2008).
6. Shao, Y., Molnar, L. F., Jung, Y., Kussmann, J., Ochsenfeld, C., Brown, S. T., Gilbert, A. T. B., Slipchenko, L. V., Levchenko, S. V., O'Neill, D. P., DiStasio Jr., R. A., Lochan, R. C., Wang, T., Beran, G. J. O., Besley, N. A., Herbert, J. M., Lin, C. Y., Van Voorhis, T., Chien, S. H., Sodt, A., Steele, R. P., Rassolov, V. A., Maslen, P. E., Korambath, P. P., Adamson, R. D., Austin, B., Baker, J., Byrd, E. F. C., Dachsel, H., Doerksen, R. J., Dreuw, A., Dunietz, B. D., Dutoi, A. D., Furlani, T. R., Gwaltney, S. R., Heyden, A., Hirata, S., Hsu, C-P., Kedziora, G., Khalliulin, R. Z., Klunzinger, P., Lee, A. M., Lee, M. S., Liang, W. Z., Lotan, I., Nair, N., Peters, B., Proynov, E. I., Pieniazek, P. A., Rhee, Y. M., Ritchie, J., Rosta, E., Sherrill, C. D., Simmonett, A. C., Subotnik, J. E., Woodcock III, H. L., Zhang, W., Bell, A. T., Chakraborty, A. K., Chipman, D. M., Keil, F. J., Warshel, A., Hehre, W. J., Schaefer, H. F., Kong, J., Krylov, A. I., Gill, P. M. W. & Head-Gordon, M. *Phys. Chem. Chem. Phys.* **8,** 3172-3191 (2006).
7. Guinier, A. *X-ray Diffraction* (Freeman, San Francisco, 1963).
8. Dressel, C., Reppe, T., Prehm, M., Brautzsch, M. & Tschierske, C. Chiral self-sorting and amplification in isotropic liquids of achiral molecules. *Nature Chem.* **6,** 971-977 (2014).
9. Nakashima, N., Yamada, N. & Kunitake, T. A Fourier transform infrared study of bilayer membranes of double-chain ammonium amphiphiles. *J. Phys. Chem.* **90,** 3374-3377 (1986).
10. Yamaguchi, Y. & Nakashima, N. Phase transition dependent fourier transform infrared spectra of polyion-complexed lipid films in Air. *Anal. Sci.* **10,** 863-866 (1994).
